# Use of the Complementarity Principle in Docking Procedures: A New Approach for Evaluating the Correctness of Binding Poses

**DOI:** 10.1021/acs.jcim.0c01382

**Published:** 2021-04-02

**Authors:** Hrvoje Rimac, Maria Grishina, Vladimir Potemkin

**Affiliations:** † Department of Medicinal Chemistry, University of Zagreb Faculty of Pharmacy and Biochemistry, Ante Kovačića 1, 10000 Zagreb, Croatia; § Laboratory of Computational Modeling of Drugs, Higher Medical and Biological School, South Ural State University, Chaikovskogo 20A, Chelyabinsk 454008, Russia

## Abstract

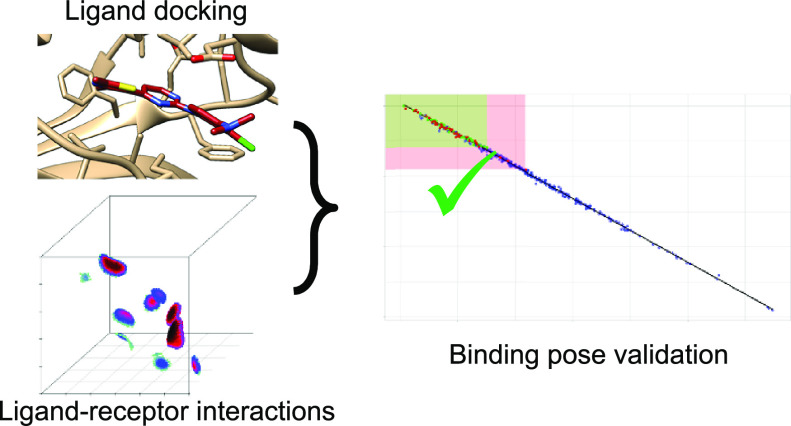

Even though the first docking procedures were developed almost 40 years ago, they are still under intense development, alongside with their validation. In this article, we are proposing the use of the quantum free-orbital AlteQ method in evaluating the correctness of ligand binding poses and their ranking. The AlteQ method calculates the electron density in the interspace between the ligand and the receptor, and since their interactions follow the maximum complementarity principle, an equation can be obtained, which describes these interactions. In this way, the AlteQ method evaluates the quality of contacts between the ligand and the receptor, bypasses the drawbacks of using ligand RMSD as a measure of docking quality, and can be considered as an improvement of the “fraction of recovered ligand–receptor contacts” method. Free Windows and Linux versions of the AlteQ program for assessing complementarity between the ligand and the receptor are available for download at www.chemosophia.com.

## Introduction

Molecular docking is a computational process in which we are trying to determine if a small molecule (ligand) binds to a macromolecule (receptor). Molecular docking can be used in predicting conformations and affinities of not yet synthesized molecules for the receptor of interest or performing a virtual screening of a database of ligands for a specific target. Additionally, it can also provide insights into the mechanism of ligand binding, identify key receptor residues responsible for ligand activity, and enable further ligand optimization to obtain a compound with optimal characteristics.^1−3^ Since this approach allows examination of a large number of potential ligands in a short time, without the need to conduct physical experiments, it is very often employed as an initial step in many drug discovery programs.^4−7^

Even though it was pioneered nearly 40 years ago,^8^ docking approaches are still under intense development, as can be seen by the use of different approaches in finding (search algorithms, divided into systematic and stochastic search methods) and evaluating (scoring functions, divided into empirical, force field-based, and knowledge-based functions) potential ligand–receptor complexes.^9^ This stems from the fact that no approach is perfect and suitable for all cases. For this reason, docking programs are constantly being updated and evaluated.^10−12^ An additional problem is validation of docking programs: if a docking program cannot reproduce a binding pose of the reference ligand, there is no guarantee that the results for other potential ligands are of any use.

A common way to assess the performance of a docking program is to compute the root-mean-square deviation (RMSD) between the docked and the reference ligand poses, i.e. the average distance between corresponding atoms in both poses. A typical RMSD cut-off for determining the ability of a docking program to reproduce the correct, most often crystallographic, pose is 2 Å. However, apart from the performance of the docking program itself, this ability can be affected by the quality of crystallographic data (e.g., if the reference structure is of bad quality, has steric clashes, missing side-chains, etc.), type of RMSD algorithm used (standard RMSD method, minimum-distance RMSD,^13^ and symmetry-corrected RMSD,^14^ to name a few), and a presence of flexible substituent groups protruding outside of the ligand pocket and not forming any significant interactions with the receptor molecule. The latter can significantly increase RMSD without having a noticeable influence on the actual binding affinity.

Another approach of validating docking results is to calculate the fraction of recovered ligand–receptor contacts^15,16^ where these drawbacks of RMSD methods are avoided. However, in this case, length cut-offs for the “ligand–receptor contacts” and “recovered ligand–receptor contacts” have to be defined. Another approach of determining ligand–receptor contacts is the use of the quantum free-orbital AlteQ method.^17^ This approach is based on the use of Slater’s type atomic contributions, and it calculates electron density in the interspace between the ligand and the receptor.^18,19^ Since all ligand–receptor interactions are determined by the overlaps of electron clouds, these interactions follow the principle of maximum complementarity and, as was recently established, can be expressed analytically (eq 1):^20^
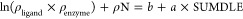
1where *b* and *a* are the parameters of the equation, the *b* coefficient is dimensionless, the *a* coefficient is measured in Å^–1^, ρ_ligand_ represents the ligand’s contribution to electron density in the *m*th point in the molecular space, ρ_enzyme_ represents the receptor’s contribution to electron density in the same point, and ρN and SUMDLE are defined in a following manner (eqs 2 and 3, respectively):
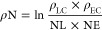
2
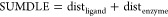
3where ρLC represents electron density in the center of the highest-contributing ligand atom, ρEC is the electron density in the center of the highest-contributing enzyme atom, NL is the atomic number of the highest-contributing ligand atom, NE is the atomic number of the highest-contributing enzyme atom, dist_ligand_ represents distance between the *m*th point and the ligand’s atom having the highest contribution to ρ_ligand_ at that point, and dist_enzyme_ represents the distance between the same *m*th point and the enzyme’s atom having the highest contribution to ρ_enzyme_ at that point. In other words, the AlteQ complementarity principle can be expressed as follows:
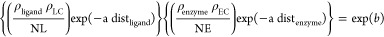
4Then, designating σ_ligand_ and σ_enzyme_ as the ligand and the enzyme contributors, respectively:
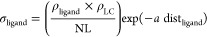
5
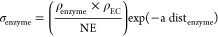
6we can obtain:
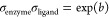
7In this sense, the AlteQ method can be used to calculate ligand–receptor contacts without having to impose any arbitrary cut-offs but solely relying on the size of atom electron clouds. By identifying ligand and receptor atoms, which contribute to electron density in the *m*th point the most as well as their distances from that point (SUMDLE), it is possible to identify and quantify the most important ligand–receptor interactions. This in turn can give valuable information about the affinity of ligand–receptor binding.

Therefore, in this paper, we propose the use of the AlteQ method for determining the correctness of docking poses with a possibility of identifying the strongest ligand–receptor interactions. First, the use of eq 1 is demonstrated on the crystallographic and minimized complexes. These results are then compared with the results of the docked complexes and the presence or absence of the most important ligand–receptor interactions in the docked conformations is identified. These results are also discussed in the context of drawbacks of the RMSD and “fraction of recovered ligand–receptor contacts” methods. Finally, apart from using eq 1 for determining and visualizing the most important ligand–receptor interactions, it was also determined that the *a* coefficient reflects the binding efficiency and can have further use in quantifying the binding affinity. The method was further checked for its robustness by testing on 200 randomly selected complexes from the PDBbind core collection (http://www.pdbbind.org.cn/).

## Materials and Methods

PDB files of various ligands bound to three different receptors were obtained from the Research Collaboratory for Structural Bioinformatics (http://www.rcsb.org/): CDK2–ligand complexes (42 different crystal structures, in total 59 different conformations, which were treated as separate complexes), HIV-1 protease–ligand complexes (38 different crystal structures, 56 different conformations), and mouse acetylcholinesterase–ligand complexes (16 different crystal structures, 25 different conformations). Additional 200 complexes were selected randomly from the PDBbind core collection database (http://www.pdbbind.org.cn/) to test the robustness of the method. The complete list of all complexes can be found in the Supporting Information. Docking and minimization preparation of all complexes, their visual inspection, and distance measurements were conducted using UCSF Chimera 1.14 (University of California, USA).^21^

Docking studies were performed using AutoDock Vina^13^ on a personal computer with Intel Core i7-6700K CPU @ 4.00GHz × 32 GB RAM, and the Windows 10 operating system. AutoDock Vina uses dispersion, hydrogen bonds, and electrostatic and desolvation components for the determination of the most probable complex conformation. For the three systems used, all corresponding complexes were aligned with the first complex of that system (namely, 1B39, 1AJV, and 1J07, for CDK2, HIV-1 protease, and mouse acetylcholinesterase, respectively). This was done to facilitate the docking procedure and to use the same box coordinates for all complexes. For all complexes, ligand and water molecules were omitted from the structure and necessary hydrogen atoms were added. All Lys, Arg, and His side chains were protonated, all Asp and Glu side chains were deprotonated, and both amino and carboxyl ends were charged using the UCSF Chimera 1.14 program. A grid was generated by the AutoGrid program^22^ and centered at the center of the binding pocket. In the case of the CDK2 complexes, the grid map was of size 25 × 25 × 25 Å with the center at 0, 30, 13; for the HIV-1 protease complexes, the corresponding values were 25 × 25 × 25 Å and 12, 22, 5; and for the acetylcholinesterase, the values were 30 × 30 × 30 Å with the center at 40, 20, 10. For the 200 PDBbind core collection complexes, the grid map size varied in size (from 25 × 25 × 25 Å to 40 × 40 × 40 Å), depending on the size and flexibility of the ligand, and for each complex, it was centered at the center of mass of the crystallographic ligand. The receptor molecules were regarded as rigid, while all ligand single bonds could rotate freely during the docking procedure. The number of modes was set to 100, exhaustiveness to 20, and energy range to 4. For each complex, five ligand conformations with the lowest free binding energies were taken into further investigation.

A simple minimization procedure was also performed in Chimera 1.14 for all the crystallographic complexes in order to remove bad contacts present in the crystallographic structures. First, the structures underwent 10 steps of the steepest descent method with a step size of 0.02 Å, after which 10 steps of the conjugate gradient method followed, with a step size of also 0.02 Å. For the protein, the AMBER ff14SB force field^23^ was used, and for the ligand AM1-BCC^24^ charges were used. In total, seven conformations for all complexes (the crystallographic conformation, five docked conformations, and the minimized conformation) underwent further analysis.

Complexes obtained in such manner (413 in total for the CDK2 complexes, 392 for the HIV-1 protease complexes, 175 for the acetylcholinesterase complexes, and 1400 for the PDBbind core collection complexes) were then subjected to the electron density analysis using the in-house developed quantum free-orbital AlteQ method.^17^ For all complexes, a linear regression model was used (eqs 1, 2, and 3) to establish a correlation between the electron cloud overlap and the distance between ligand and receptor atoms. The data acquisition time for the 1400 PDBbind conformations ranged from 9.88 to 198.36 s, with a median acquisition time of 52.36 s for the Windows 10 operating system, and from 5.46 to 92.66 s, with a media acquisition time of 27.02 s for the Ubuntu 20.04 LTS operating system. More detailed data is available in Supporting Information.

The obtained data was statistically processed in R 3.6.2.^25^ using RStudio 1.2.5033 and “tidyverse”, “ggplot2”, “scatterplot3d”, “rgl”, and “RColorBrewer” libraries. For each complex, points were clustered using hierarchical clustering based on their 3D coordinates, with clusters representing locations where ligand and enzyme electron clouds partially overlap, i.e., where there is an interaction between the ligand and the enzyme. Clustering was performed using the “single” linkage method of the “hclust” command. This method is a variation of the “minimal spanning tree” method^26^ and adopts the “friends of friends” (FOF) clustering strategy. The FOF relation is defined between two points if they are friends or if they are contained in the transitive closure of the friend relation (e.g., A and C are a friend-of-friend pair via B). Since electron density was calculated every 0.1 Å (1000 points in 1 Å^3^), the clustering threshold was also a distance in the Cartesian coordinate system of 0.1 Å. RMSD calculations were performed manually for all atoms to account for molecular symmetry, analogously to the Hungarian symmetry-corrected RMSD method implemented by Allen and Rizzo.^14^

Since transcendental functions, such as logarithm and exponentiation functions in this article, act upon and deliver dimensionless numerical values, arguments of these functions have to be rendered dimensionless as well, i.e., they have to be divided by the measure function. In this article, it was done implicitly (a more thorough insight into this topic can be found in Matta et al^27^).

## Results

For each of the studied complexes for all three systems, seven ligand conformations were obtained (the crystallographic, five docked, and the minimized conformation) and for each complex, the minimized conformation was taken as the reference conformation. RMSD values for all CDK2, HIV-1 protease, and acetylcholinesterase complexes are summarized in Table 1 (a more detailed RMSD information can be found in the Supporting Information). It must be pointed out here that the docking exhaustiveness was purposefully not set very high to limit the amount of search and the energy range was set to 4. This was done to ensure that other ligand conformations, besides the global minimum, would be found, and these conformations would be kept, so the performance of the algorithm could be tested.

**Table 1:** RMSD Values for the Crystallographic and the Docked Ligand–Receptor Conformations to their Corresponding Minimized Conformations (All Values Are in Å)

	crystallographic		docked	
	mean ± s.d.	range	mean ± s.d.	range
CDK2	0.238 ± 0.093 (*N* = 59)	0.099 to 0.503 (*N* = 59)	4.397 ± 3.163 (*N* = 295)	0.099 to 13.477 (*N* = 295)
HIV-1 protease	0.185 ± 0.041 (*N* = 56)	0.120 to 0.261 (*N* = 56)	2.746 ± 1.348 (*N* = 280)	0.275 to 9.300 (*N* = 280)
acetylcholinesterase	0.217 ± 0.051 (*N* = 25)	0.137 to 0.313 (*N* = 25)	5.182 ± 3.126 (*N* = 125)	0.494 to 11.900 (*N* = 125)

### Comparison of the Crystallographic and Minimized Complexes

**Figure 1:**
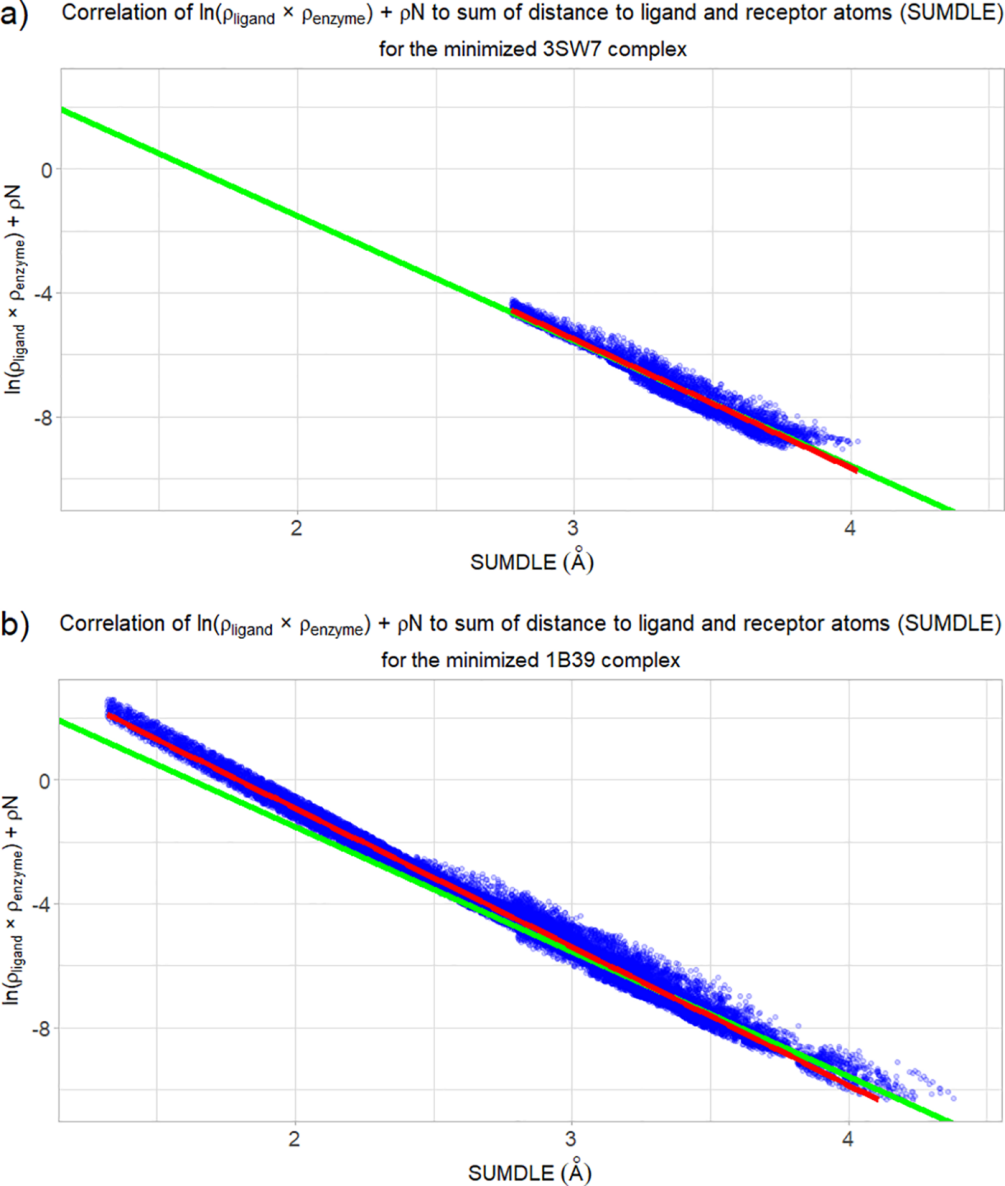
Correlation of the sum of ln(ρ_ligand_ × ρ_enzyme_) and ρN to the sum (SUMDLE) of the distances of a point to the highest-contributing ligand and enzyme atoms for the minimized complexes. The averaged regression line of all minimized complexes is shown in green, and the regression line for the selected complex is shown in red: (a) the minimized 3SW7 complex and (b) the minimized 1B39 complex.

**Figure 2:**
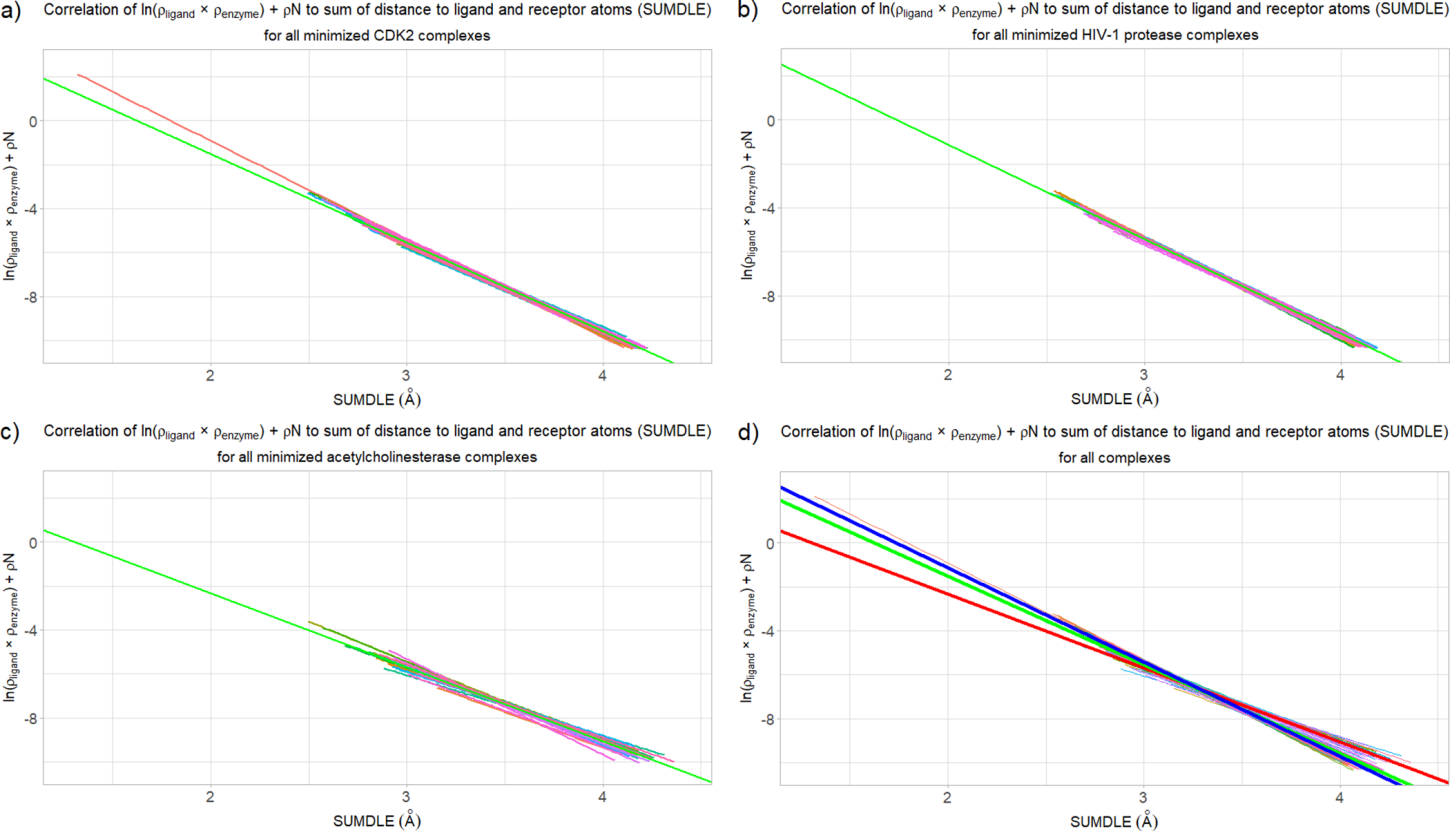
Correlation of the sum of ln(ρ_ligand_ × ρ_enzyme_) and ρN to the sum (SUMDLE) of the distances of a point to the highest-contributing ligand and enzyme atoms for the minimized complexes with the corresponding averaged regression line shown in green (for panels a, b, and c): (a) CDK2 complexes, (b) HIV-1 protease complexes, (c) acetylcholinesterase complexes, and (d) all complexes (the averaged regression lines are shown in green, blue, and red, for the CDK2, HIV-1 protease, and acetylcholinesterase complexes, respectively).

Based on the data obtained using the AlteQ method, a regression line for all complexes was obtained using eq 1 (Table 2). An example for the minimized complex 3SW7 can be seen in Figure 1a. The red regression line (for the minimized 3SW7 complex) and the green regression line (the average of all minimized CDK2 complexes) practically overlap. This is true for all minimized complexes, regardless of the ligand structure, indicating that they form very similar interactions with the binding site, which is represented by the green regression line with an intercept of 6.52 ± 0.56 (coefficient *b* in eq 1) and a slope of −4.03 ± 0.16 (coefficient *a* in eq 1). The minimized complex with the highest difference in absolute values of both coefficients was the 1B39 complex (the complex with the native ATP ligand), with coefficients *b* and *a* being equal to 8.02 and −4.47, respectively (Figure 1b). What is immediately noticeable when comparing the minimized 3SW7 and 1B39 complexes is that the 1B39 complex achieves much lower SUMDLE values. This can be attributed to the fact that ATP is a native ligand and a donor of a phosphate group, so it has to interact covalently with the enzyme. Therefore, the distance between some of the ligand and receptor atoms is very low (less than 1.5 Å). Even when the 1B39 complex is included in the calculation of the average coefficients, the values of the *b* and *a* coefficients are well preserved among all minimized ligand poses, as can be seen in Figure 2a. Also, it can be seen that the 1B39 complex is the only complex with SUMDLE values lower than 2.5 Å. As a comparison, for the crystallographic complexes, the values of the coefficients are 6.59 ± 0.67 and – 4.06 ± 0.19 for the *b* and *a* coefficients, respectively. It can be seen that the coefficients’ values are practically the same for the crystallographic and minimized complexes, with crystallographic complexes having a higher standard deviation.

**Table 2:** Values of the *b* and *a* Coefficients for the Crystallographic and the Minimized Ligand– Receptor Conformations

	crystallographic		minimized	
	intercept (coefficient *b*) mean ± s.d.	slope (coefficient *a*) mean ± s.d.	intercept (coefficient *b*) mean ± s.d.	slope (coefficient *a*) mean ± s.d.
CDK2	6.59 ± 0.67	–4.06 ± 0.19	6.52 ± 0.56	–4.03 ± 0.16
HIV-1 protease	7.82 ± 0.73	–4.40 ± 0.22	7.41 ± 0.59	–4.28 ± 0.17
acetylcholinesterase	4.68 ± 1.46	–3.45 ± 0.43	4.37 ± 1.20	–3.36 ± 0.34
PDBbind complexes	6.25 ± 1.63	–3.94 ± 0.47	6.31 ± 1.51	–3.95 ± 0.44

For the HIV-1 protease complexes, the values of the *b* and *a* regression line coefficients were 7.41 ± 0.59 and −4.28 ± 0.17 for the minimized and 7.82 ± 0.73 and −4.40 ± 0.22 for the crystallographic complexes. These results partially overlap with the results for the CDK2 complexes. However, the values of the *b* and *a* coefficients for the acetylcholinesterase complexes are significantly different, with the *b* coefficient being 4.37 ± 1.20 and the *a* coefficient being −3.36 ± 0.34 in the case of the minimized and 4.68 ± 1.46 and – 3.45 ± 0.43 in the case of the crystallographic complexes. Graphs for the minimized HIV-1 protease and acetylcholinesterase complexes are analogous to the CDK2 complexes and can be seen in Figure 2b,c, respectively.

### Comparison of the Docked and Minimized Complexes

**Figure 3:**
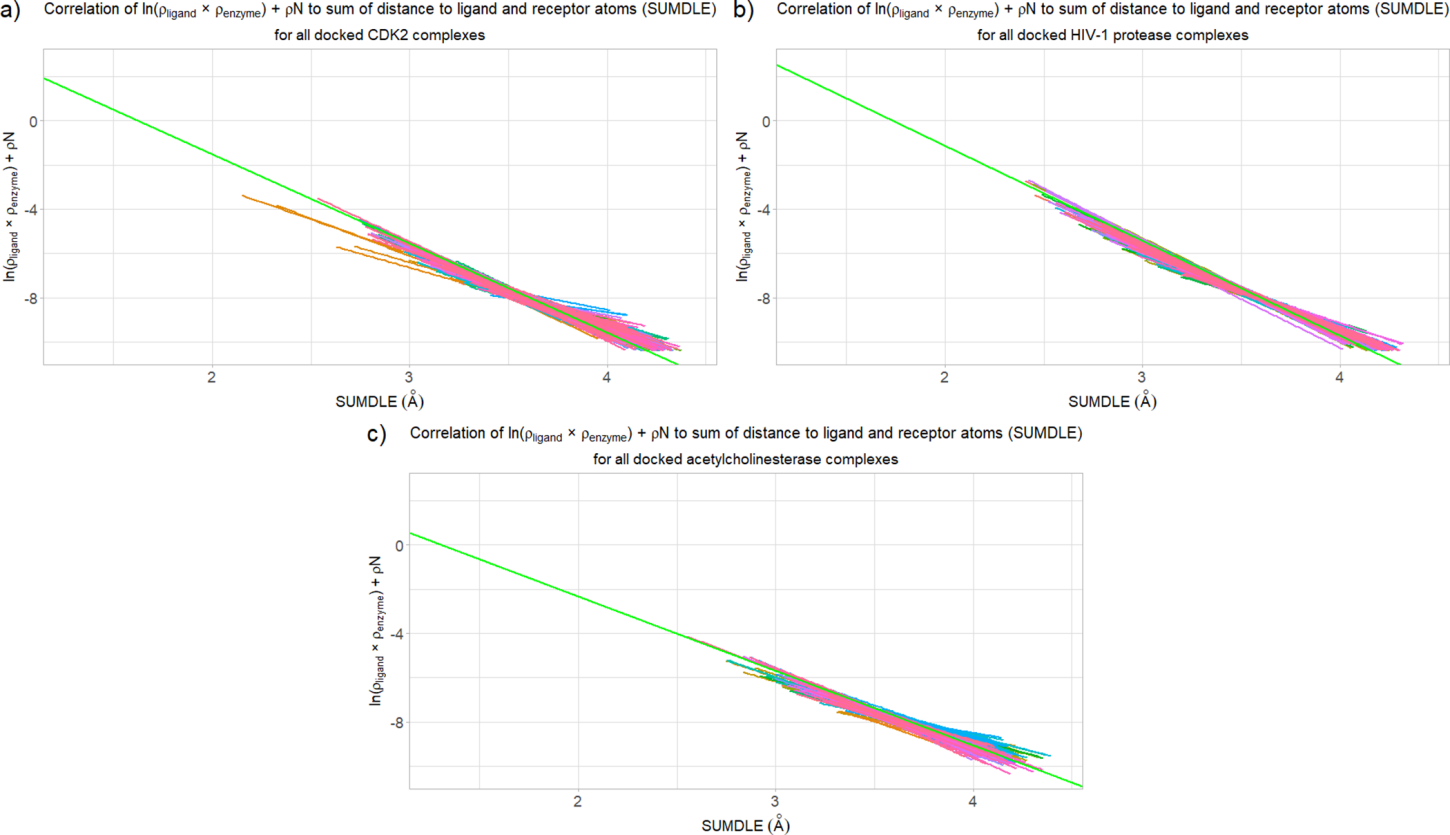
Correlation of the sum of ln(ρ_ligand_ × ρ_enzyme_) and ρN to the sum (SUMDLE) of distances of a point to the highest-contributing ligand and enzyme atoms for the docked complexes with the corresponding averaged regression line for the corresponding minimized complexes shown in green: (a) CDK2 complexes, (b) HIV-1 protease complexes, and (c) acetylcholinesterase complexes.

**Figure 4:**
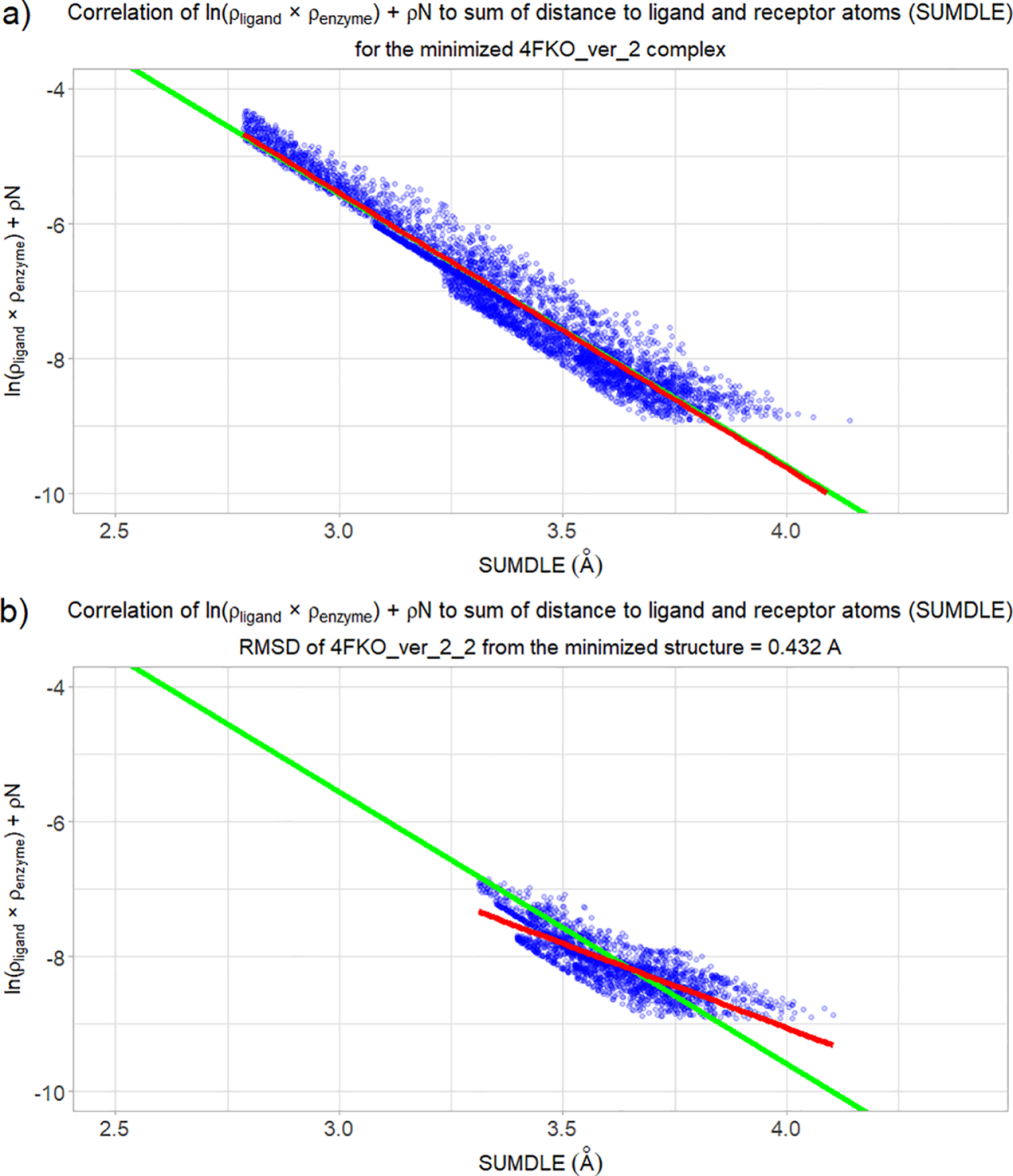
Correlation of the sum of ln(ρ_ligand_ × ρ_enzyme_) and ρN to the sum (SUMDLE) of distances of a point to the highest-contributing ligand and enzyme atoms for (a) the minimized 4FKO_ver_2 complex, (b) the best docked 4FKO_ver_2 complex. The regression line of the studied complex is shown in red, while the averaged regression line for all minimized CDK2 complexes is shown in green.

For the docked complexes, the situation is completely different (Figure 3a–c). There are some complexes that have similar values of the *b* and *a* coefficients to the averaged ones, but the regression lines for the majority of complexes are significantly different. In Figure 4a,b we can see a comparison between the minimized 4FKO_ver_2 complex and the best docked 4FKO_ver_2 complex (as a side note, this complex with the symmetry-corrected RMSD of 0.432 Å is the complex with the lowest RMSD among all docked CDK2 complexes). The regression line of the minimized 4FKO_ver_2 complex is practically identical to the “correct” regression line, while the regression line of the best docked complex is very different. Another point to notice is that the docked complex is lacking all ligand–receptor interactions shorter than 3.3 Å.

**Figure 5:**
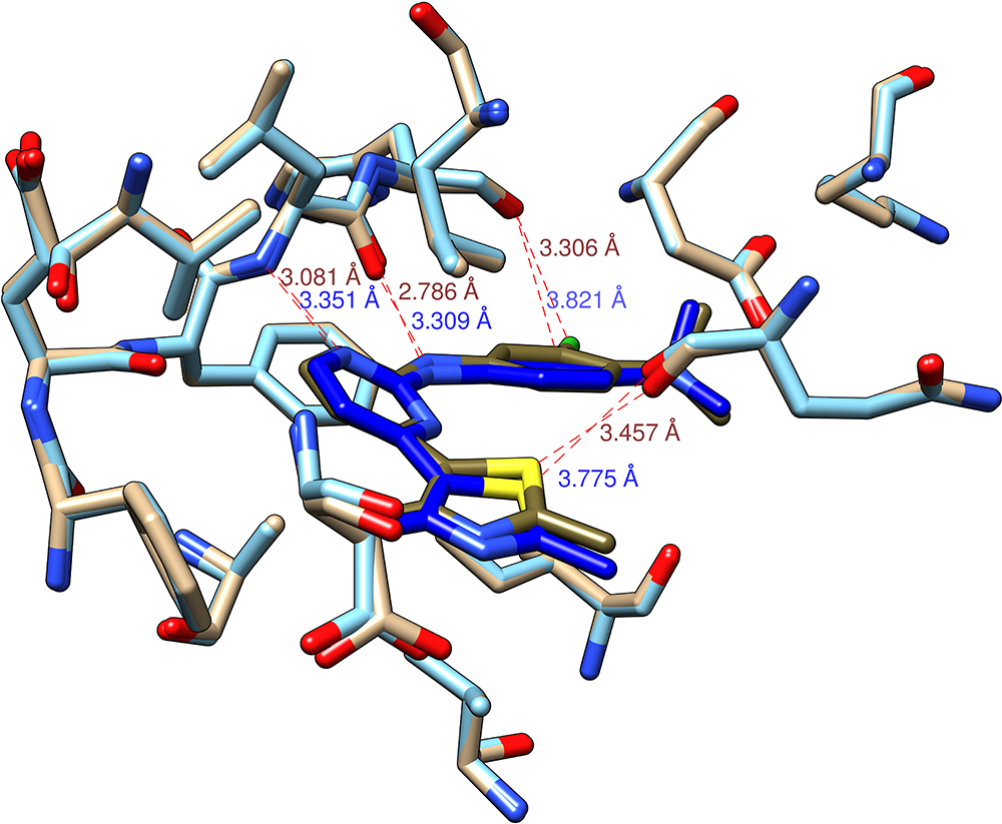
Superimposition of the minimized (brown and tan) and best docked (dark and light blue) 4FKO_ver_2 complexes with certain distances to the receptor shown.

If we superimpose the minimized and the best docked 4FKO_ver_2 complex (Figure 5), we can see that they have practically the same conformation inside the binding pocket. However, the distances between the same ligand and receptor atoms can differ significantly. In such a way, estimating docking accuracy solely on RMSD scores can be misleading, as the strongest ligand–receptor interactions can be neglected. Likewise, basing the ligand–receptor interaction analysis entirely on the docking results can also bring about wrong conclusions. One way to increase the accuracy of the docking results can be to perform geometry optimization of the complex, as was done in the case of CDK2 inhibitors by Bagheri et al.^15^ This allows a recovery of some ligand–receptor interactions but comes at the expense of computation time. However, it has to be said that poses obtained by docking in this manner are still good starting structures for molecular dynamics (MD) simulations, as the first step of MD simulations is geometry optimization, which “corrects” these initial poses.

**Figure 6:**
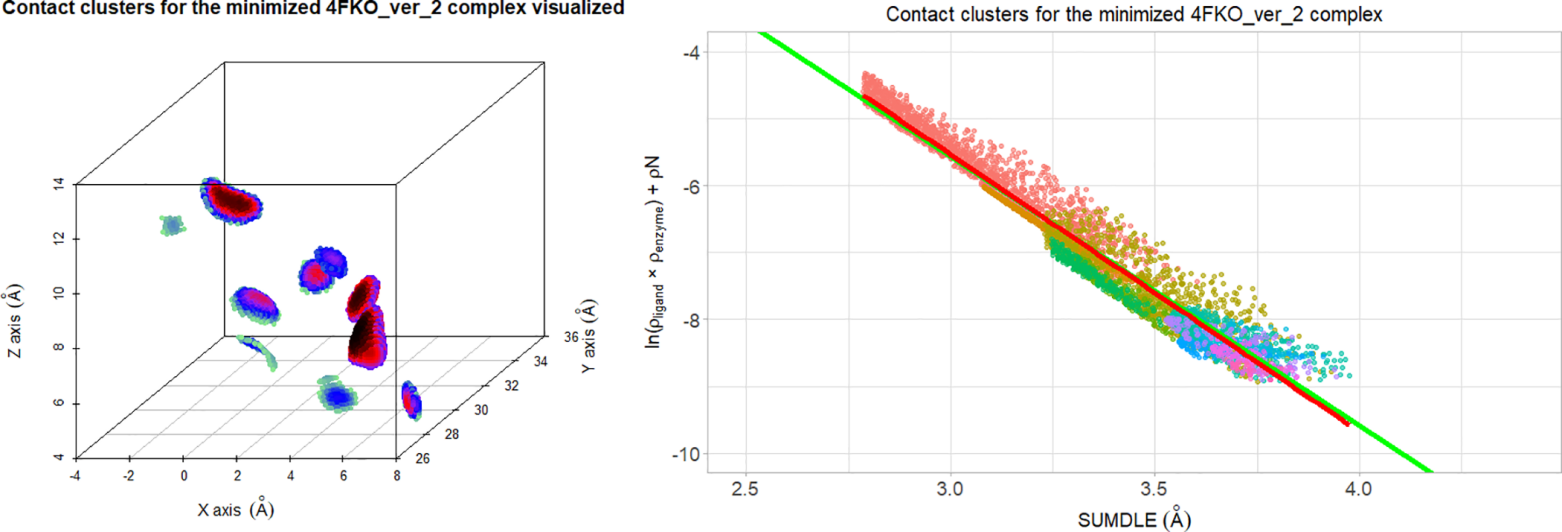
Contact clusters (11 in total) for the minimized 4FKO_ver_2 complex visualized: in 3D space (points are colored by values of ln(ρ_ligand_ × ρ_enzyme_): black for the highest values, through red, purple, blue, and light green for the lowest values) (left) and by correlation of ln(ρ_ligand_ × ρ_enzyme_) and ρN to SUMDLE, and colored by clusters (right). The averaged regression line of all minimized complexes is shown in green, and the regression line for the selected complex is shown in red.

When the points in the intermolecular space where there is an overlap between ligand and receptor electron clouds are visualized (Figure 6 left), the number of their contacts can be obtained (in the case of the minimized 4FKO_ver_2 complex, that number is 11). Also, the ln(ρ_ligand_ × ρ_enzyme_) + ρN versus SUMDLE graph (Figure 6 right) can be colored by clusters to show how these clusters contribute to the equation coefficients.

**Figure 7:**
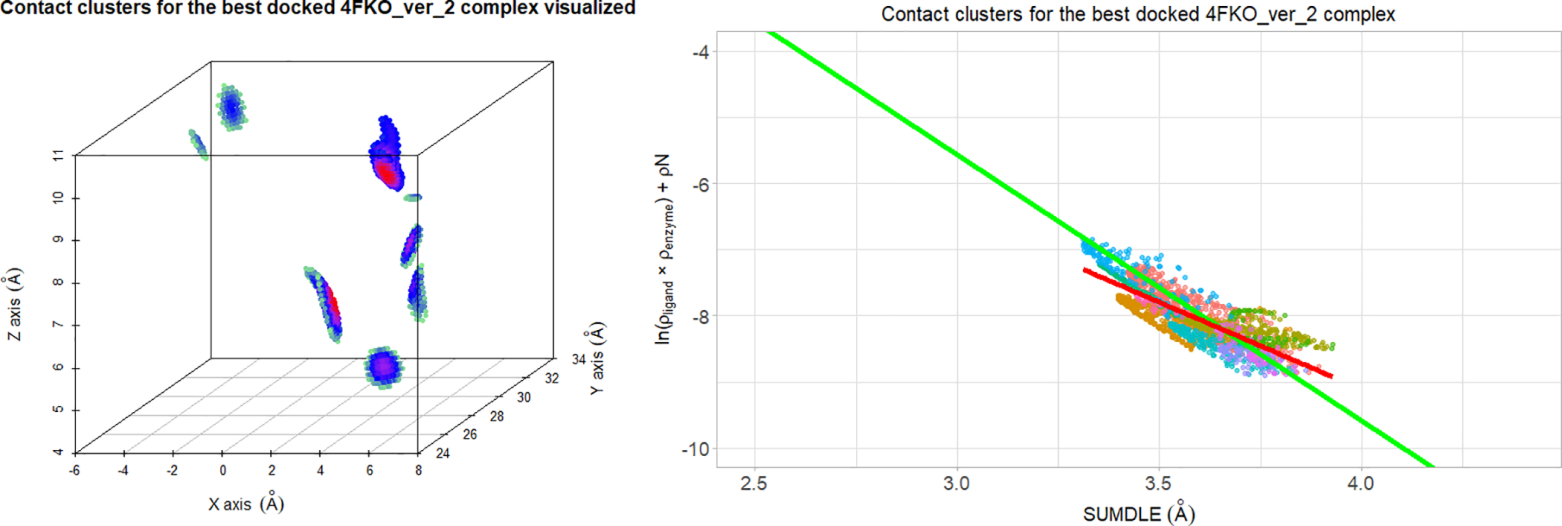
Contact clusters (10 in total) for the best docked 4FKO_ver_2 complex visualized: in 3D space (points are colored by values of ln(ρ_ligand_ × ρ_enzyme_): black for the highest values, through red, purple, blue, and light green for the lowest values) (left) and by correlation of ln(ρ_ligand_ × ρ_enzyme_) and ρN to SUMDLE and colored by clusters (right). The averaged regression line of all minimized complexes is shown in green, and the regression line for the selected complex is shown in red.

If the same analysis is done for the best docked complex (Figure 7), we can see that the pattern of interactions is completely different, with less overlaps between ligand and receptor electron clouds (lack of clusters with points with black color, left part of Figure 7). This again confirms the fact that docking procedures, despite being able to obtain very similar poses to the correct ones, can miss important interactions and their distances.

**Figure 8:**
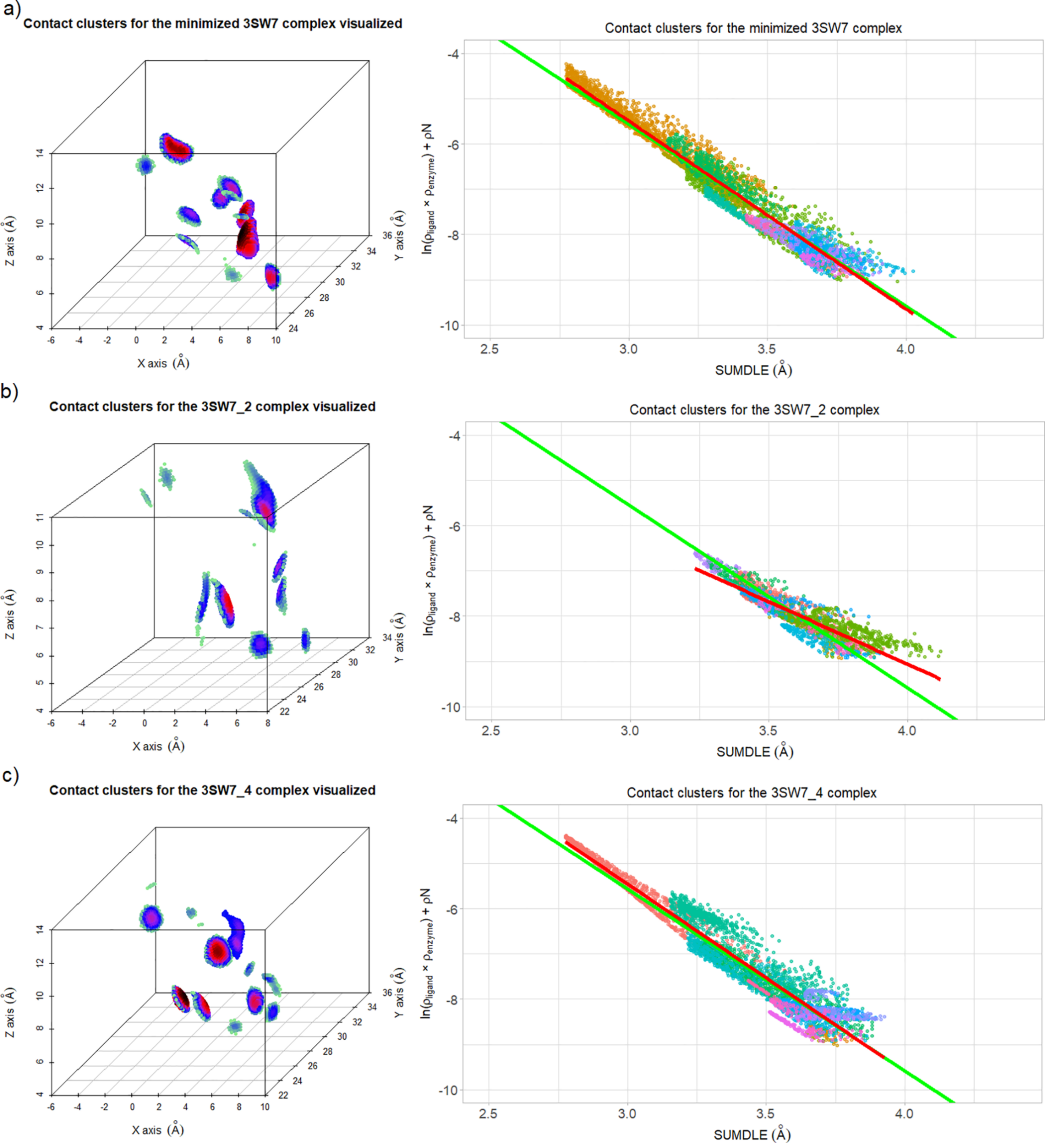
Contact clusters for the 3SW7 complexes in 3D space (points are colored by values of ln(ρ_ligand_ × ρ_enzyme_): black for the highest values, through red, purple, blue, and light green for the lowest values) (left) and by correlation of ln(ρ_ligand_ × ρ_enzyme_) and ρN to SUMDLE, and colored by clusters (right): (a) for the minimized 3SW7 complex (11 clusters in total), (b) for the 3SW7_2 complex (the best docked complex, 11 clusters in total), and (c) for the 3SW7_4 complex (16 clusters in total). The averaged regression line of all minimized complexes is shown in green, and the regression line for the selected complex is shown in red.

On the other hand, some docked ligand poses with higher RMSD can have better preserved interactions with the receptor atoms than ligands with a lower RMSD. One such example are the minimized complex 3SW7 (Figure 8a), the best docked 3SW7 complex (3SW7_2, RMSD from the minimized complex 0.667 Å) (Figure 8b), and complex 3SW7_4 (RMSD from the minimized complex 2.416 Å) (Figure 8c).

**Figure 9:**
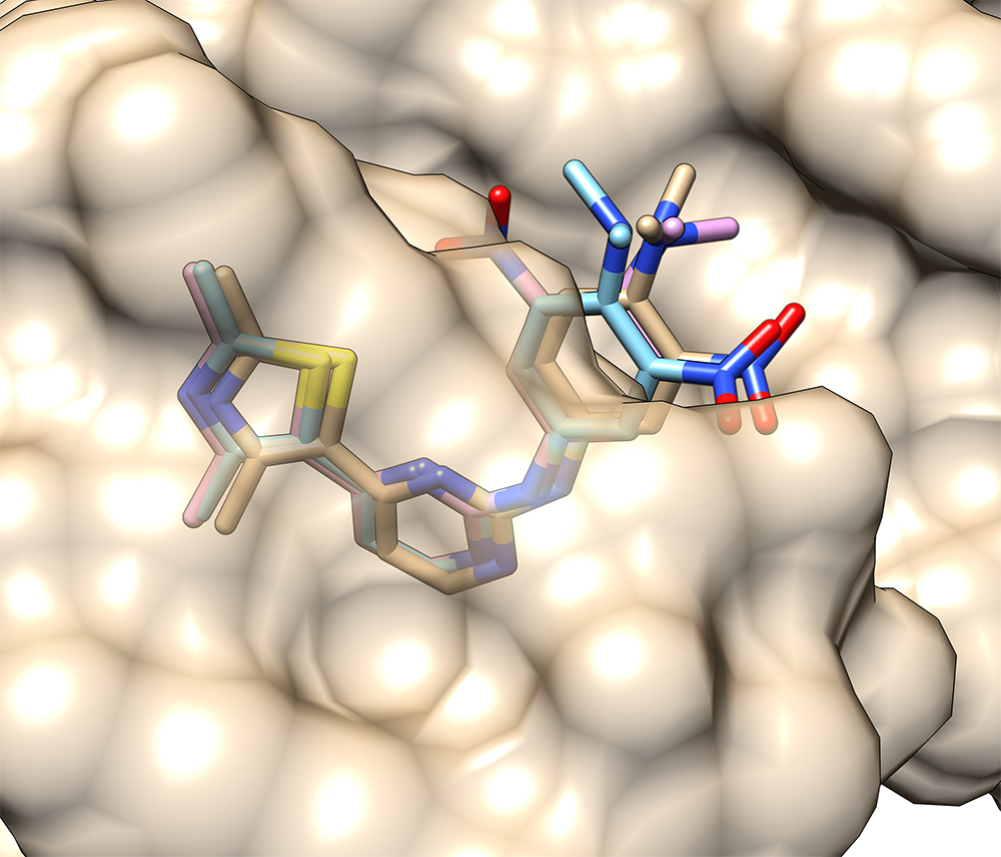
Overlay of the minimized 3SW7 complex (tan), the best docked 3SW7 complex (3SW7_2, light blue), and the 3SW7_4 complex (pink) obtained using the MSMS package^28^ in UCSF Chimera 1.14.^21^

From both the left and right sides of Figure 8, it can be seen that the ligand–receptor contacts in the minimized complex resemble contacts in the 3SW7_4 much more than in the 3SW7_2, even though the 3SW7_2 complex has a lower RMSD. This can also be seen in their overlay (Figure 9). These results can be explained by the position of the *N,N*-dimethyl-*o*-nitrobenzene group, which, in the 3SW7_4 complex, is rotated by approximately 180° but does not form any significant interactions since it is protruding out of the binding pocket. However, even if we neglect this group, the RMSD of the 3SW7_2 complex is still lower than that of the 3SW7_4 complex (RMSD values are 1.237 and 3.518 Å and decrease to 0.484 and 0.637 Å, respectively, if the *N,N*-dimethyl-*o*-nitrobenzene group is not considered) with positions of their contact clusters being also significantly different.

This contribution to the RMSD of substituent groups, which do not significantly interact with receptor atoms, poses a problem in docking validation if docking results are judged solely on the RMSD score. One way to tackle this problem, as it was done by Bagheri et al.^15^ and Feinstein and Brylinski,^16^ is by using the fraction of recovered ligand–receptor contacts, which was defined in a following way: “the contacts were identified for interatomic distances less than 4.5 Å between any pair of heavy atoms, one from the ligand and one from the receptor. The difference of less than 1.0 Å between the predicted contact and the corresponding contact in the experimental structure was considered as a correct recovery of the contact.” Analogously, the similarity of the regression lines obtained by using eq 1 and the AlteQ method for the docked and minimized poses can be regarded as an alternative method of checking the recovered ligand–receptor contacts. The advantage of this approach is that there is no need to introduce cut-offs for interatomic distances, as the strength of ligand–receptor contacts are determined by overlaps of their electron clouds.

**Figure 10:**
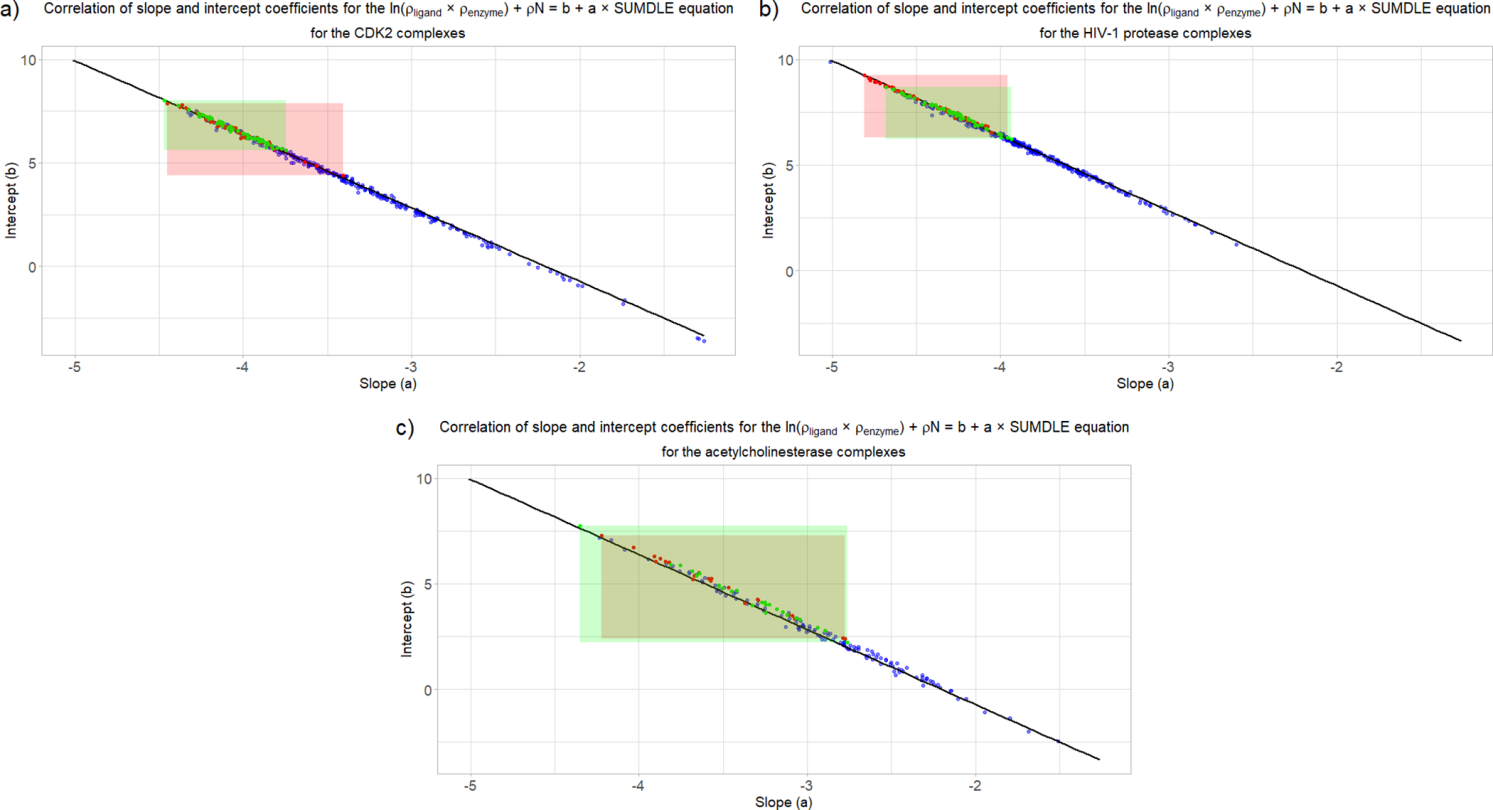
Correlation of slope and intercept coefficients for all: (a) CDK2, (b) HIV-1 protease, and (c) acetylcholinesterase complexes. Docked complexes are shown in blue, minimized complexes are shown in green, and crystallographic complexes are shown in red. Green and red rectangles show the areas above where all the minimized and crystallographic complexes are located, respectively.

**Figure 11:**
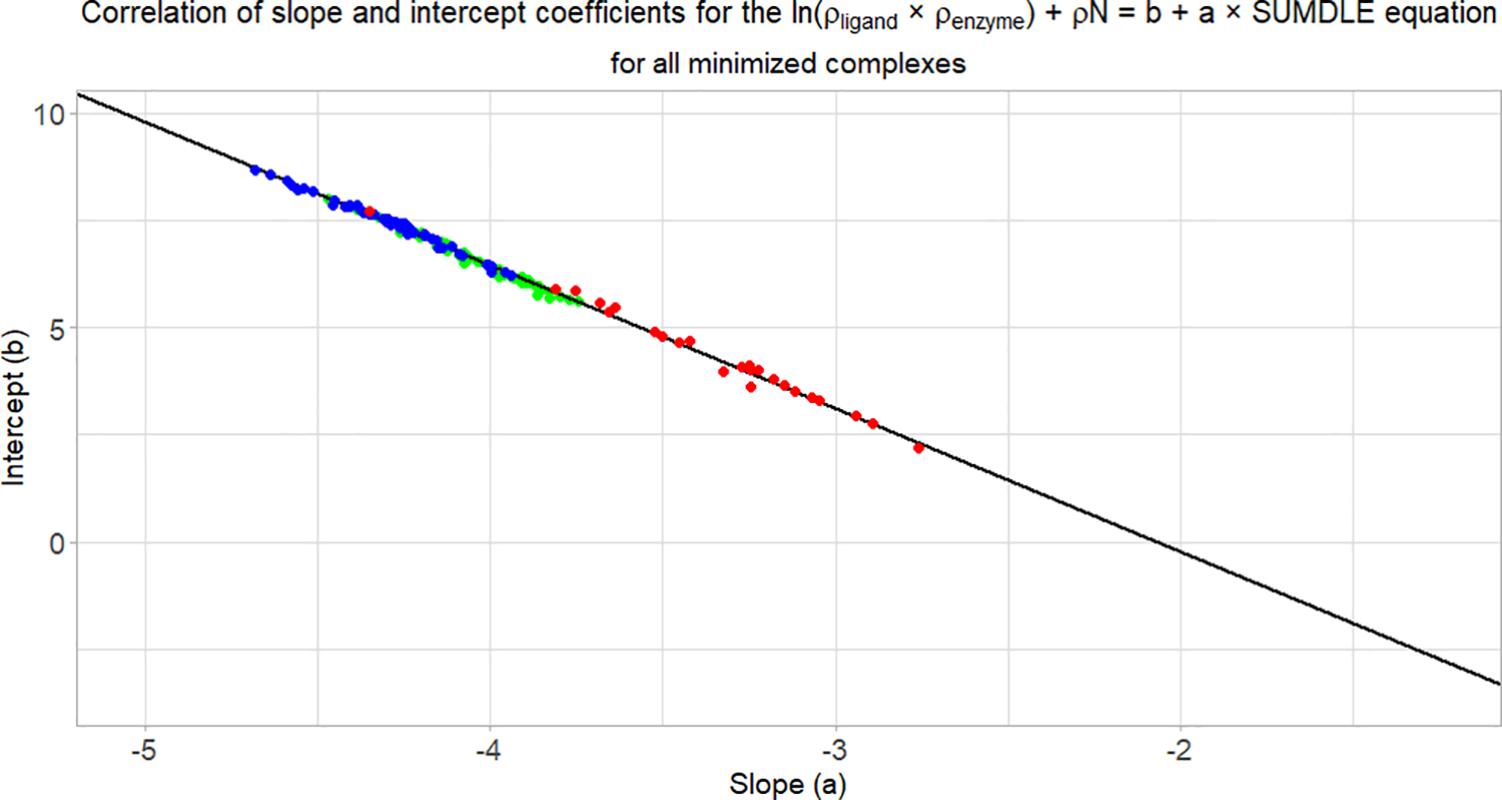
Comparison of all minimized complexes based on their *b* and *a* coefficients: CDK2 complexes are shown in green, HIV-1 protease complexes are shown in blue, and acetylcholinesterase complexes are shown in red.

In a more simplified manner, apart from comparing the shape of the graphs obtained through eq 1, the correctness of docked poses can be determined from the intercept versus slope graph. When the slope and intercept coefficients for all tested complexes are correlated, they all fall onto the same regression line (Figure 10), with adjusted *R*^2^ = 0.9980 (*N* = 413), *R*^2^ = 0.9965 (*N* = 392), and *R*^2^ = 0.9937 (*N* = 175). It can be seen that the docked complexes (blue) have a wide range of slopes and intercepts, while the crystallographic (red) and minimized (green) complexes have a significantly lower range of possible values (this can also be seen from Figures 2 and 3). The CDK2 crystallographic complexes that are located outside of the green rectangle (Figure 10b) are 3LFS (*a* = −3.63), 2C5N_ver_2 (*a* = −3.56), and 1PF8 (*a* = −3.41). These complexes also have crystallographic data of a very bad quality. In these cases, minimizing the complexes significantly improves their conformations and consequently their *b* and *a* coefficients, which are afterward more in line with other complexes. For the docked complexes, lower *b* coefficients and higher *a* coefficients can be explained by the unsuccessfulness of the docking algorithm to find the correct pose and these points represent docking poses with higher binding energies (the results for individual complexes can be found in the Supporting Information). If all the minimized complexes are compared (Figure 11), it can be seen that the HIV protease complexes in general have the highest values of the *b* coefficient and the lowest values of the *a* coefficient (blue line), followed by the CDK2 complexes (green line), and acetylcholinesterase complexes (red line). Since the adjusted *R*^2^ is in all cases very close to 1, all ligands can be defined just by their slope coefficient value (*a*), leaving out the *b* coefficient.

**Figure 12:**
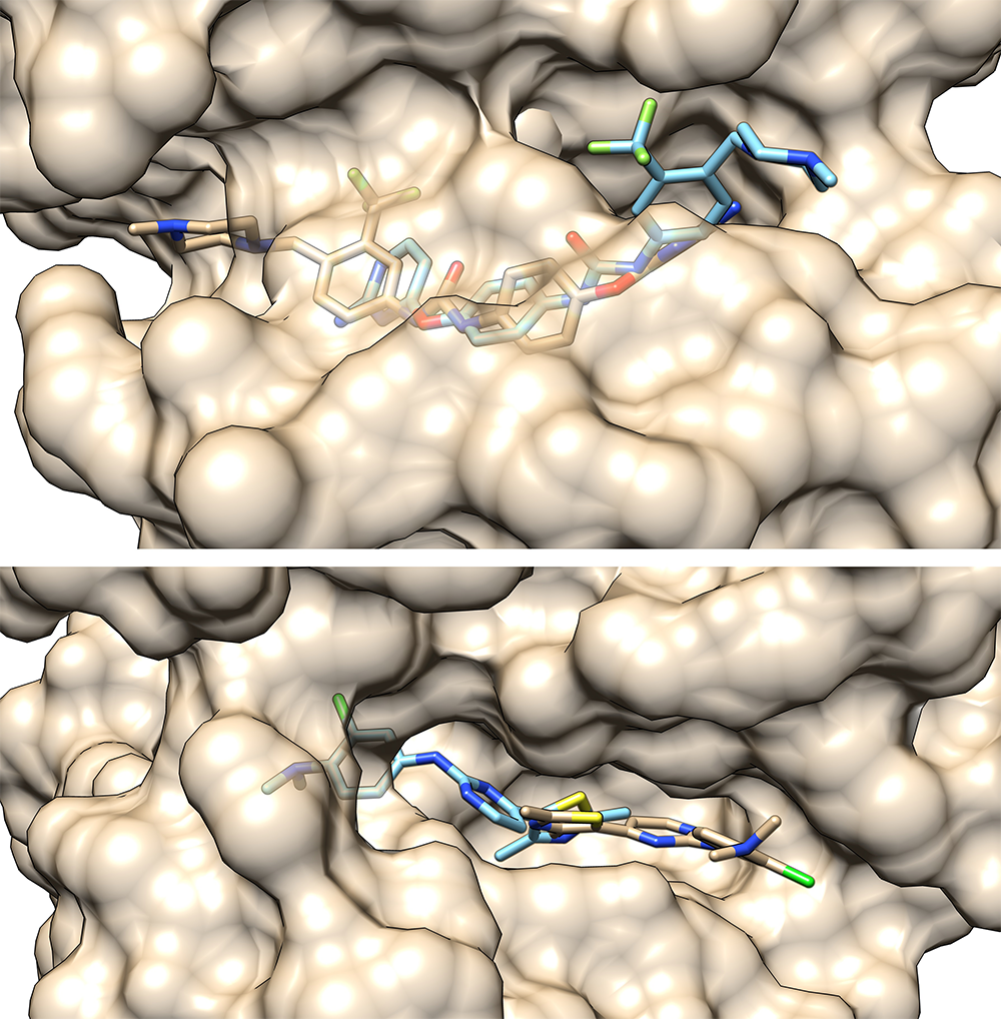
Comparison of the minimized (tan) and docked (blue) ligands in complexes 5A14_4 (upper) and 4FKO_ver_1_3 (lower) obtained using the MSMS package^28^ in UCSF Chimera 1.14.^21^

As an example, from all the docked CDK2 complexes with the *a* coefficient between the highest and the lowest values of the minimized complexes (the green rectangle in Figure 10a), two complexes with the highest RMSD values are 5A14_4 and 4FKO_ver_1_3 with RMSD values 13.477 and 11.297 Å, respectively (Figure 12). In these cases, docking did not obtain minimized poses; however, these two poses partially overlap with the minimized ones, forming very similar interactions as the minimized complex, so they are energetically similar and satisfy eq 1, with coefficients indistinguishable from the minimized ones. It is possible that such poses (5A14_4 and 4FKO_ver_1_3) could represent alternative or metastable poses but were not obtained experimentally due to other factors, e.g., complexes with such ligand poses for some reason or another are not able to form a crystal.

The preliminary results of comparing CDK2, HIV-1 protease, and acetylcholinesterase crystallographic data show that the *a* coefficient reflects the binding efficiency. On the one hand, it has rather low values for the crystallographic and minimized complexes compared to most docked complexes of the same receptor, and on the other hand, it has to obey the Pauli exclusion principle, i.e., the occupied electronic levels cannot overlap. This also explains the lower standard deviation of the *b* and *a* coefficients for the minimized complexes, compared to the docked ones. The range of binding energies can be best observed in the case of the acetylcholinesterase complexes, as here, the ligands vary the most in their size and structure. However, full validation of this approach, including the exact relationship and the error of the method still need to be determined, as this exceeds the subject of this article and will be published separately.

### Comparison of the PDBbind Core Collection Complexes

**Figure 13:**
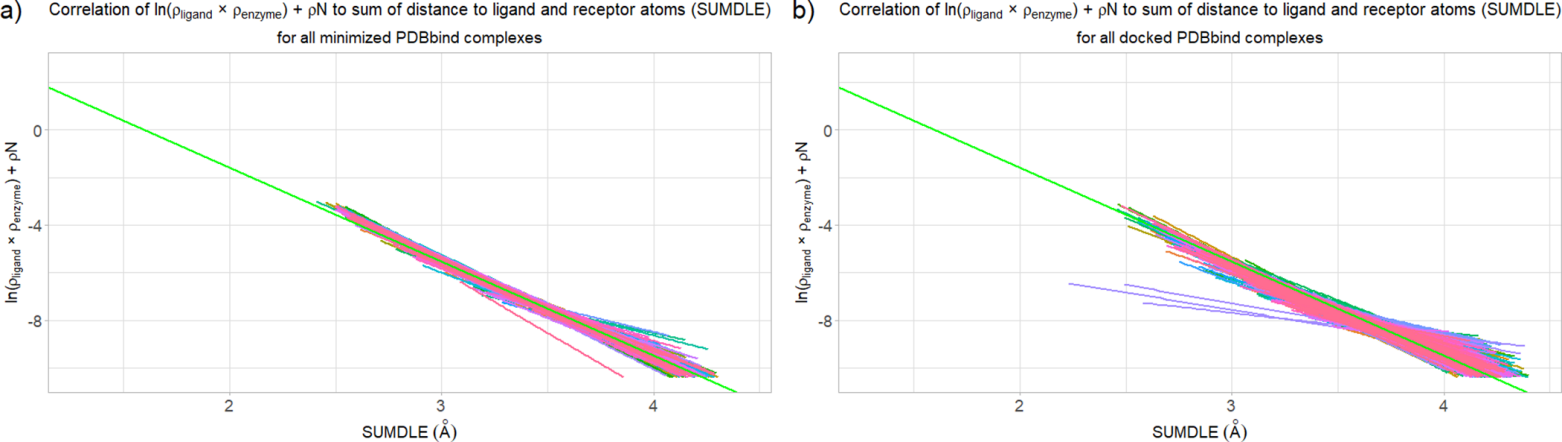
Correlation of the sum of ln(ρ_ligand_ × ρ_enzyme_) and ρN to the sum (SUMDLE) of the distances of a point to the highest-contributing ligand and enzyme atoms for the (a) minimized and (b) docked tested PDBbind complexes with the corresponding averaged regression line shown in green.

The 200 randomly selected complexes taken from the PDBbind core collection database were used to test the robustness of the proposed AlteQ method. After following the same minimization and docking protocols, for each complex, seven different ligand–receptor conformations were obtained (1400 in total). These conformations were then analyzed using the AlteQ method to obtain their *b* and *a* coefficients. As can be seen from Figure 13, regression lines for the docked complexes are much more dispersed than those for the minimized complexes, indicating a wider range of binding affinities for the docked compounds, as was expected.

**Figure 14:**
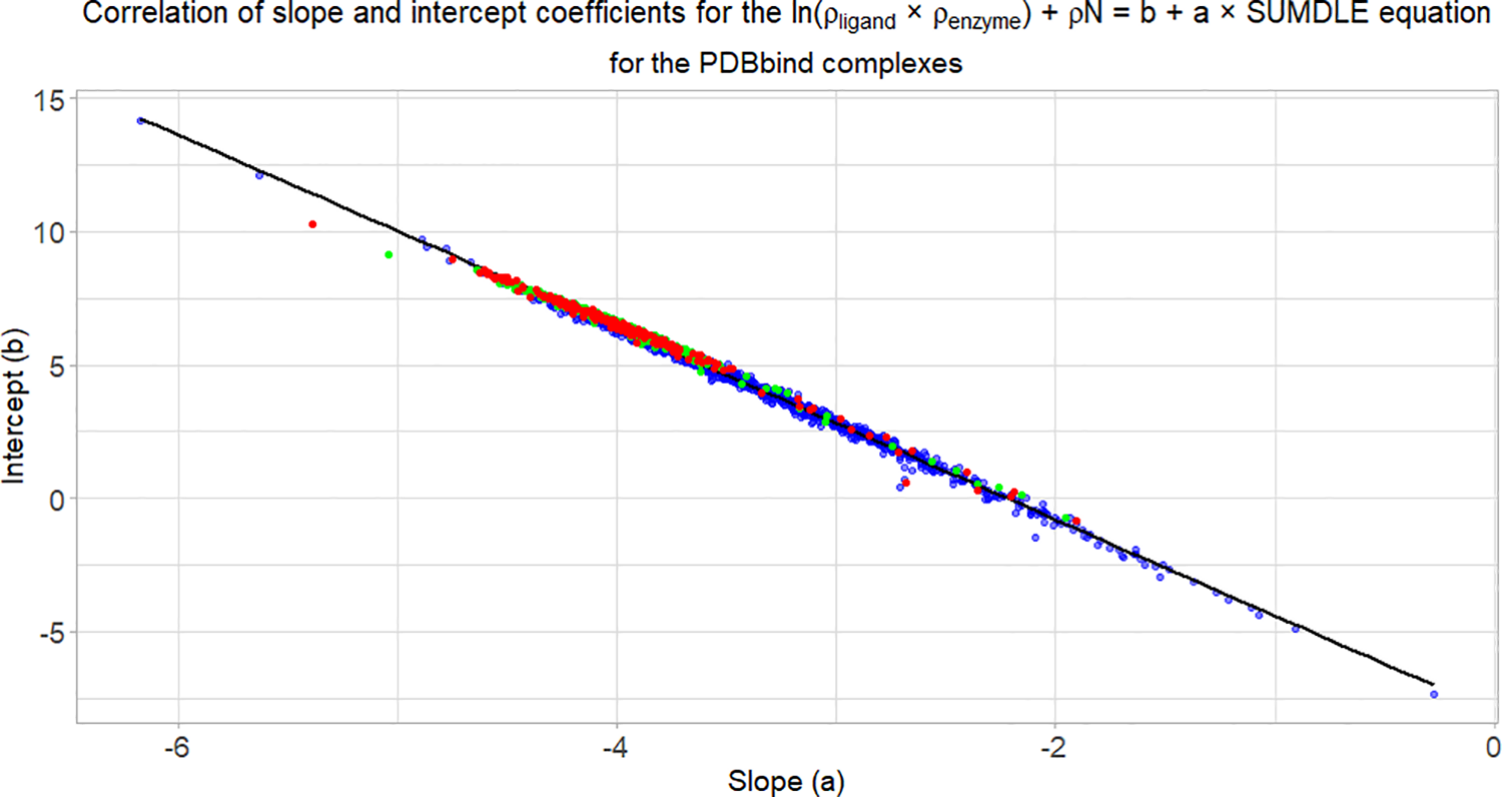
Correlation of slope and intercept coefficients for all tested PDBbind core collection complexes. Docked complexes are shown in blue, minimized complexes are shown in green, and crystallographic complexes are shown in red.

Analogously to Figure 10 and the conclusion that the coefficient *a* can be regarded as a measure of the ligand’s binding affinity, Figure 14 shows a correlation between the *b* and *a* coefficients for all 1400 tested conformations. Again, it is immediately noticeable that the docked complexes have a wider range of the *b* and *a* coefficients, while the minimized and crystallographic complexes tend to be grouped together, with the exception of smaller ligands (molecular fragments), which tend to have the slope coefficient *a* > −3.5 (the results for individual complexes can be found in the Supporting Information). Additionally, as is the case for the three systems discussed earlier, the correlation between the slope and intercept coefficients is very high, with the adjusted *R*^2^ = 0.9952.

This is a confirmation that the AlteQ method is a robust method that can be applied to a wide variety of ligand–receptor complexes with the same quality of results.

## Discussion

This study highlights the problem of using RMSD, a measure of intermolecular differences in position and conformation, as criteria for assessing the efficacy of docking programs, since higher RMSD does not necessarily correspond to changes in key protein–ligand interactions. RMSD, being a scalar quantity, an average of changes in locations of all atoms in the molecule, does not give any information about the type of positional change (e.g., translation or rotation) or which parts of the molecule are well aligned, and which are not. There are several variations in calculating RMSD: the standard RMSD method, the minimum-distance RMSD, and the symmetry-corrected RMSD, to name a few. A problem with the standard RMSD method is the fixed one-to-one correspondence, which does not consider molecular symmetry and the existence of equivalent atoms. For example, a rotation of the isopropyl group by 180° will yield two different RMSDs, even though the two structures are equivalent. This results in the overestimation of the true RMSD. Another approach is the minimum-distance RMSD, as employed in AutoDock Vina.^13^ This method measures the distance between atoms of the same type but in some cases may not enforce a one-to-one atom correspondence. Under these circumstances, some atoms could be used multiple times for the RMSD calculation, while others could be neglected, resulting in an underestimation of the true RMSD. Finally, there is the symmetry-corrected RMSD, as employed in DOCK.^14^ This method considers the symmetry of the molecule and adjusts the atom-to-atom correspondence, with all atoms being used in the calculation. In this way, the obtained RMSD value is always equal or lower than the value obtained by the standard RMSD. However, even this method has its drawbacks in calculating the correspondence between the “correct” and the docked conformation, as it does not include information about molecular flexibility and ligand–receptor interactions. The problem of flexibility is much more prominent in calculating the RMSD of proteins,^29^ but it is important in ligand docking as well, especially when a part of the ligand is outside of the binding pocket. For example, in Figure 9, the *N,N*-dimethyl-*o*-nitrobenzene group is rotated by approximately 180° but this does not significantly influence interactions with the receptor molecule, since it is protruding out of the binding pocket. Therefore, its rotation has no significant influence on ligand’s binding energy. An alternative method for determining correctness of binding poses is by using the fraction of recovered ligand–receptor contacts, where the presence of such flexible substituents that form no significant interactions does not influence the final result. In its standard form, this method employs cut-offs for distances and distance differences between the docked and reference complexes. This can lead to different results based on what values of cut-offs are employed. Employment of the AlteQ method for docking purposes can circumvent this problem by defining and calculating the strength of the contacts between ligand and receptor atoms based on the overlaps of their electron clouds and the assessments of which, as well as a topological analysis of the electron density of large biomolecular systems, assessment of physicochemical properties, biological activity, and comparisons of the AlteQ method with other quantum-chemical methods are described in recent publications.^19,30,31^ In such a way, an equation for optimal ligand–receptor interactions can be obtained which needs to be satisfied by the docked pose to consider it correct. Additionally, based on Figure 10, the slope coefficient *a* can be used as a tool for ranking of different docking poses of the same ligand as it is correlated with their binding energy. It has to be pointed out here that there could exist several alternative binding poses, which do not correspond to the crystallographic pose but have very similar binding energies (e.g., Figure 12) and therefore have a similar shape of eq 1 to that of the minimized complex.

Future prospects in the precise search for the correct docking pose may be based on modeling of ligand–receptor structures considering also the hydrogen atoms and evaluating overlaps of inner electron shells, which, according to the Pauli principle, should be zero even for covalently bound atoms. Likewise, in the current state, the AlteQ program is more suitable for an in-depth analysis of ligand–receptor interactions and targeted docking and is not optimized for routine virtual screening tasks. However, the *a* coefficient was shown to be correlated to ligand binding affinity so its use for ranking ligand poses and predicting their binding constants is also being studied. This could further broaden the use of the AlteQ method to include virtual screening tasks.

## Conclusions

Since RMSD criteria for validation of docking results has its flaws, alternative methods for assessing the correctness of results are also being developed. One such method is using the fraction of recovered ligand–receptor contacts. In this study, we presented the AlteQ method, which can be considered as an improvement of the classical form of this method, as it avoids using arbitrary cut-offs but calculates the strength of contacts between ligand and receptor atoms directly from their electron densities. In such a manner, the most important interactions (and their strength) between the ligand and the receptor can be identified, but also the coefficient *a*, which corresponds to the binding constant, can be obtained. This method represents an alternative way of determining ligand–receptor interactions based on overlaps of their electron clouds and can be used to rank different docking poses of the same ligand while identifying the strongest interactions with the receptor.

## Supporting Information Available

Structures of all studied complexes, Windows and Linux versions of the AlteQ program, calculation duration data, RMSD data for the three tested systems, and coefficients *b* and *a* values for all complexes (ZIP)

## Notes

Raw AlteQ data is available free of charge at www.chemosophia.com.
