# Supplementary figures and images for: Use of the Complementarity Principle in Docking Procedures: A New Approach for Evaluating the Correctness of Binding Poses (part 2 of 2)
Source: J Chem Inf Model. 2021 Apr 2;61(4):1801–13. doi: 10.1021/acs.jcim.0c01382 (PMC8154257; doi:10.1021/acs.jcim.0c01382)

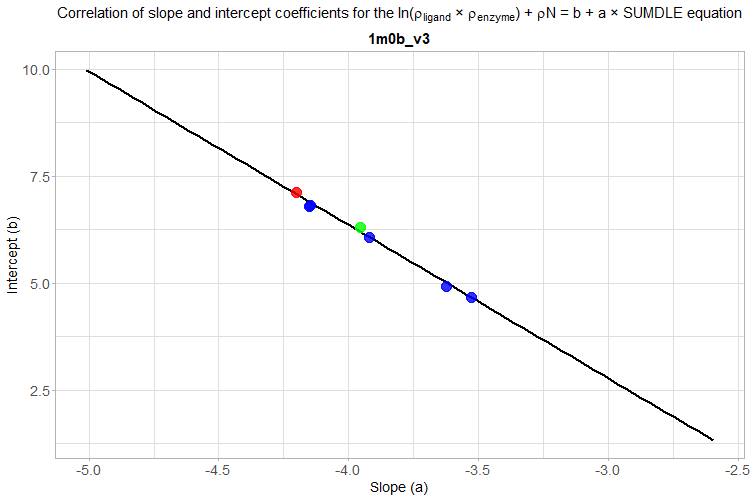

Supplement: Supplementary file 1 — ci0c01382_si_001.zip [file ci0c01382_si_001.zip › Supporting_Information/electron_density_data/HIV_1_protease/Coefficient data/1m0b_v3.png]

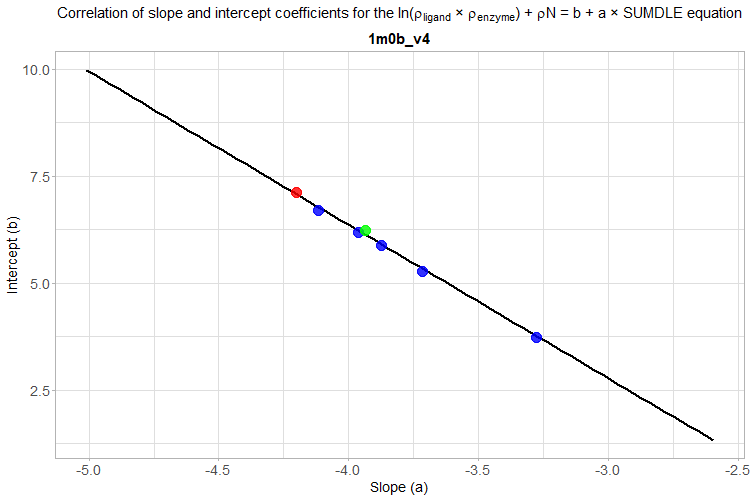

Supplement: Supplementary file 1 — ci0c01382_si_001.zip [file ci0c01382_si_001.zip › Supporting_Information/electron_density_data/HIV_1_protease/Coefficient data/1m0b_v4.png]

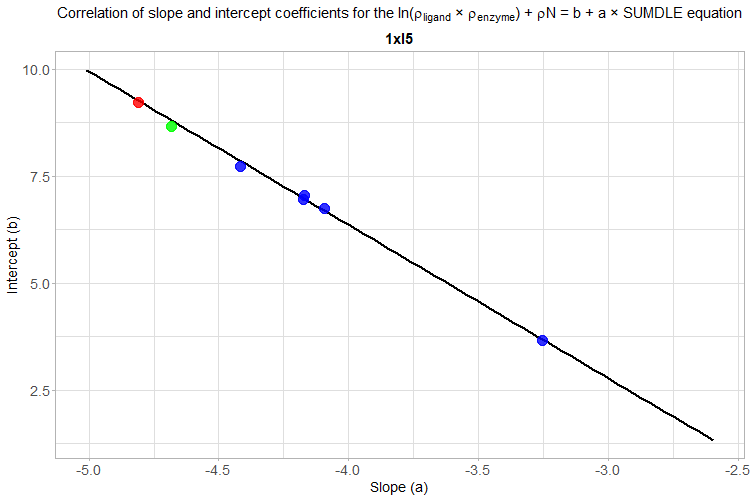

Supplement: Supplementary file 1 — ci0c01382_si_001.zip [file ci0c01382_si_001.zip › Supporting_Information/electron_density_data/HIV_1_protease/Coefficient data/1xl5.png]

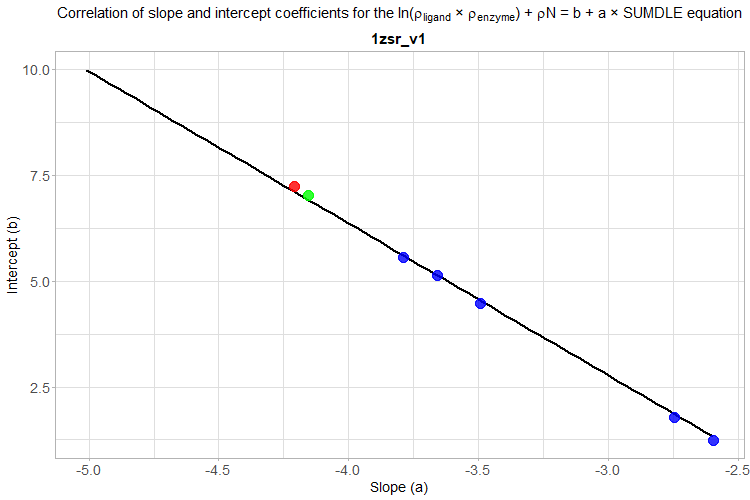

Supplement: Supplementary file 1 — ci0c01382_si_001.zip [file ci0c01382_si_001.zip › Supporting_Information/electron_density_data/HIV_1_protease/Coefficient data/1zsr_v1.png]

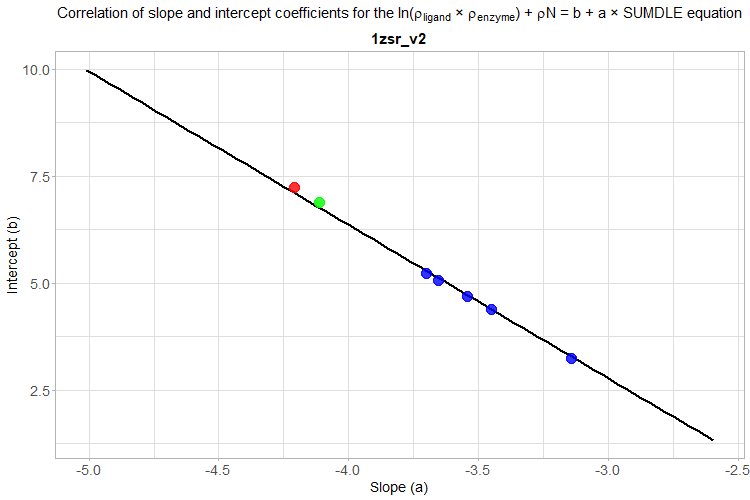

Supplement: Supplementary file 1 — ci0c01382_si_001.zip [file ci0c01382_si_001.zip › Supporting_Information/electron_density_data/HIV_1_protease/Coefficient data/1zsr_v2.png]

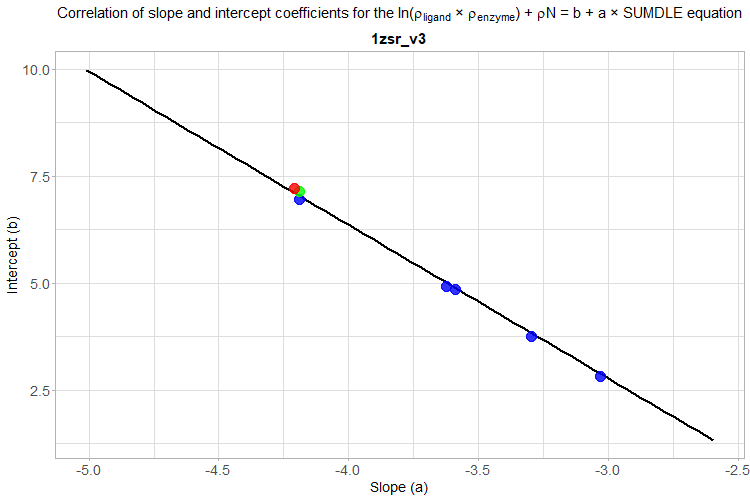

Supplement: Supplementary file 1 — ci0c01382_si_001.zip [file ci0c01382_si_001.zip › Supporting_Information/electron_density_data/HIV_1_protease/Coefficient data/1zsr_v3.png]

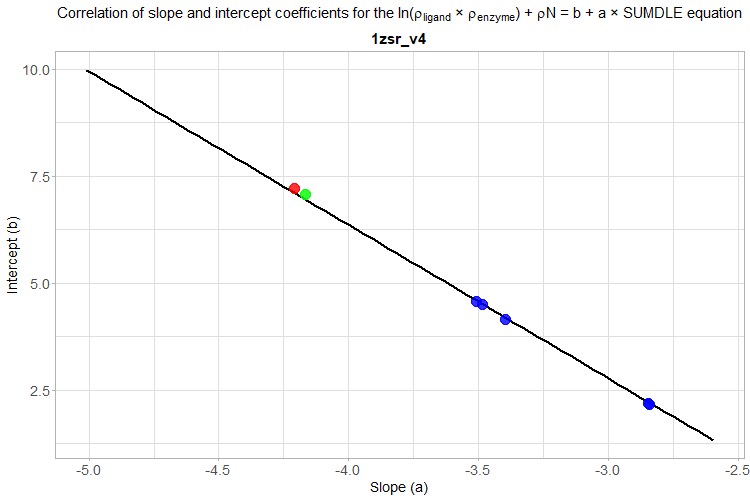

Supplement: Supplementary file 1 — ci0c01382_si_001.zip [file ci0c01382_si_001.zip › Supporting_Information/electron_density_data/HIV_1_protease/Coefficient data/1zsr_v4.png]

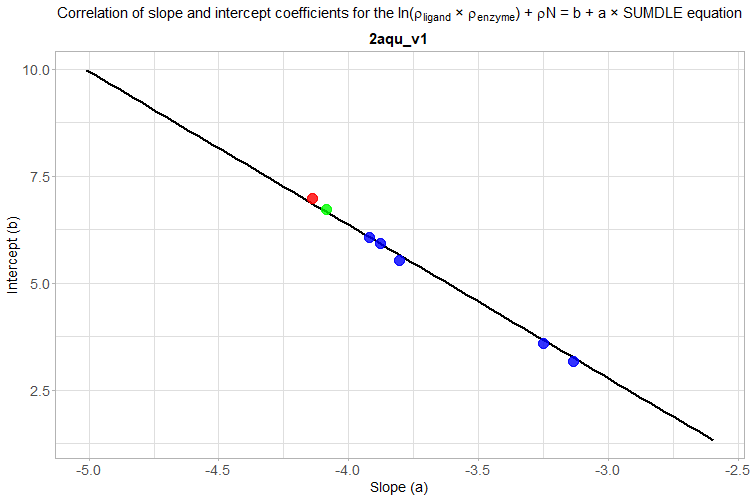

Supplement: Supplementary file 1 — ci0c01382_si_001.zip [file ci0c01382_si_001.zip › Supporting_Information/electron_density_data/HIV_1_protease/Coefficient data/2aqu_v1.png]

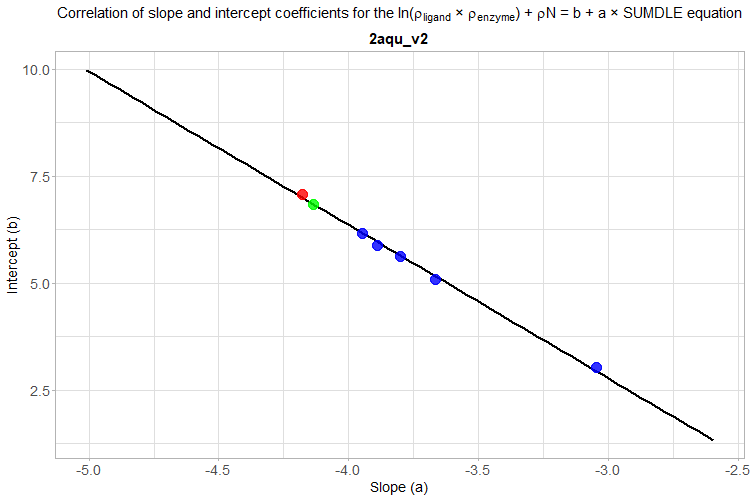

Supplement: Supplementary file 1 — ci0c01382_si_001.zip [file ci0c01382_si_001.zip › Supporting_Information/electron_density_data/HIV_1_protease/Coefficient data/2aqu_v2.png]

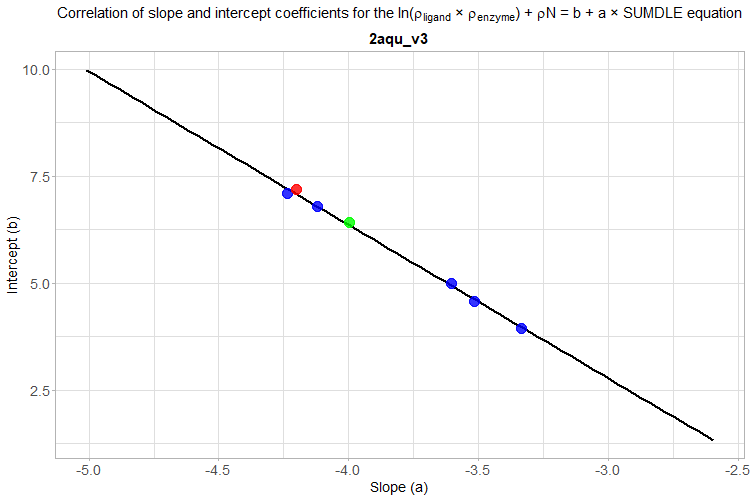

Supplement: Supplementary file 1 — ci0c01382_si_001.zip [file ci0c01382_si_001.zip › Supporting_Information/electron_density_data/HIV_1_protease/Coefficient data/2aqu_v3.png]

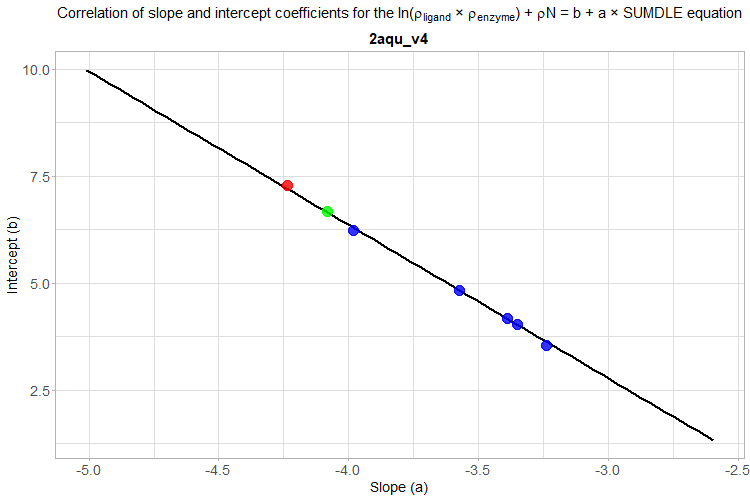

Supplement: Supplementary file 1 — ci0c01382_si_001.zip [file ci0c01382_si_001.zip › Supporting_Information/electron_density_data/HIV_1_protease/Coefficient data/2aqu_v4.png]

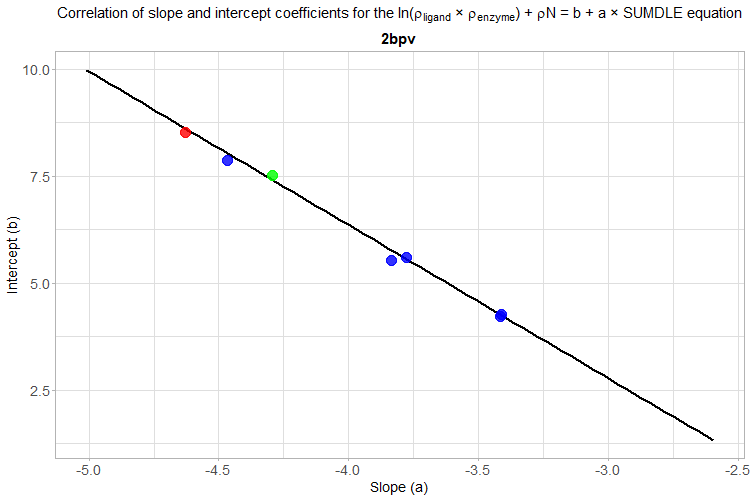

Supplement: Supplementary file 1 — ci0c01382_si_001.zip [file ci0c01382_si_001.zip › Supporting_Information/electron_density_data/HIV_1_protease/Coefficient data/2bpv.png]

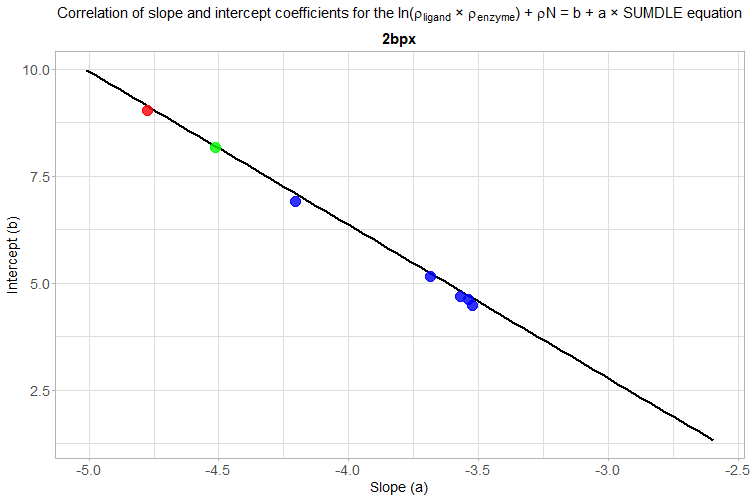

Supplement: Supplementary file 1 — ci0c01382_si_001.zip [file ci0c01382_si_001.zip › Supporting_Information/electron_density_data/HIV_1_protease/Coefficient data/2bpx.png]

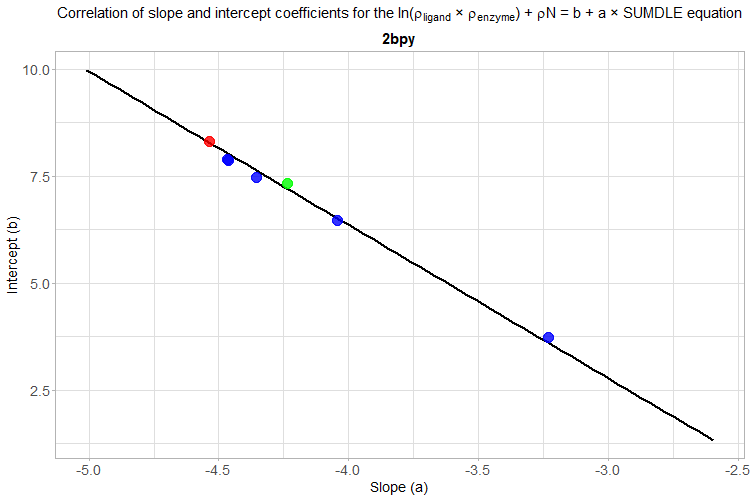

Supplement: Supplementary file 1 — ci0c01382_si_001.zip [file ci0c01382_si_001.zip › Supporting_Information/electron_density_data/HIV_1_protease/Coefficient data/2bpy.png]

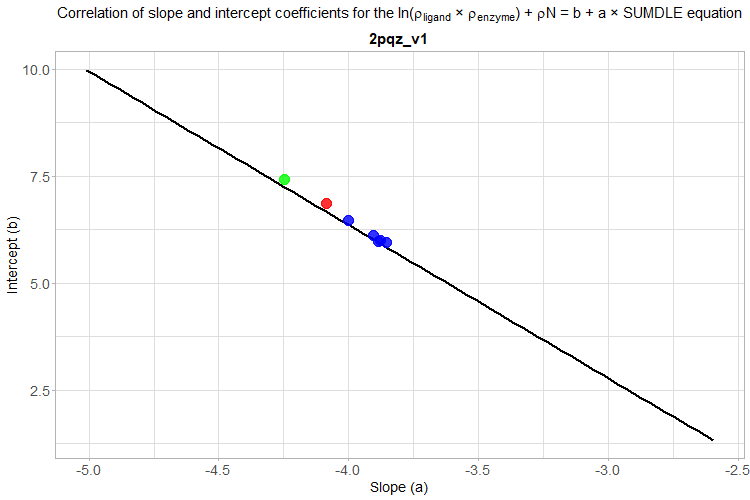

Supplement: Supplementary file 1 — ci0c01382_si_001.zip [file ci0c01382_si_001.zip › Supporting_Information/electron_density_data/HIV_1_protease/Coefficient data/2pqz_v1.png]

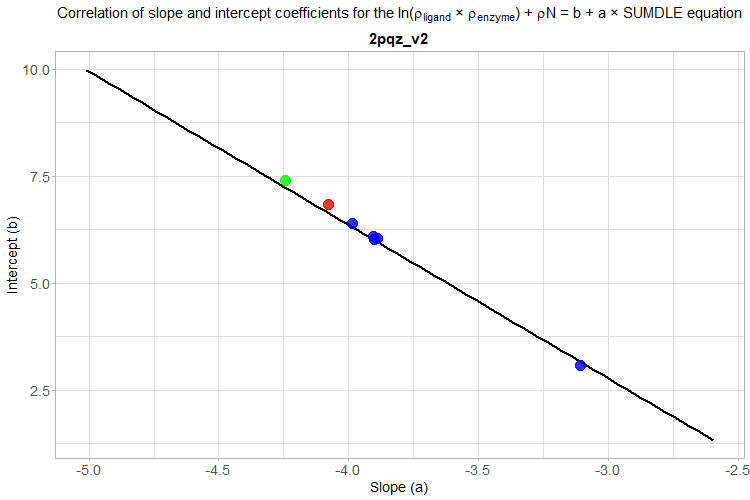

Supplement: Supplementary file 1 — ci0c01382_si_001.zip [file ci0c01382_si_001.zip › Supporting_Information/electron_density_data/HIV_1_protease/Coefficient data/2pqz_v2.png]

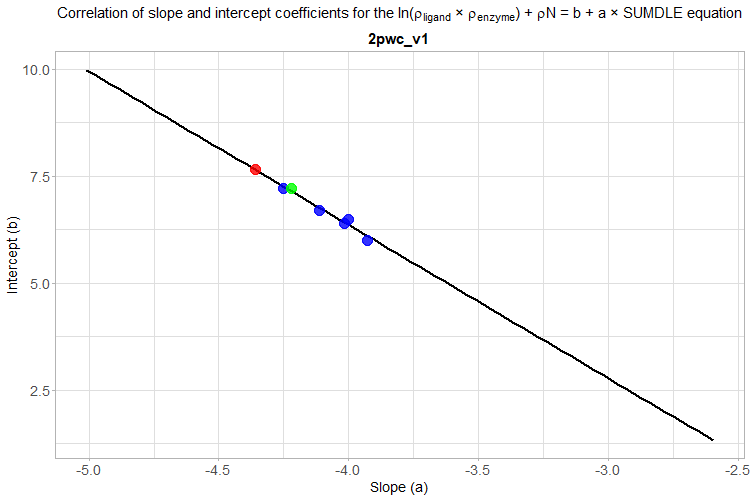

Supplement: Supplementary file 1 — ci0c01382_si_001.zip [file ci0c01382_si_001.zip › Supporting_Information/electron_density_data/HIV_1_protease/Coefficient data/2pwc_v1.png]

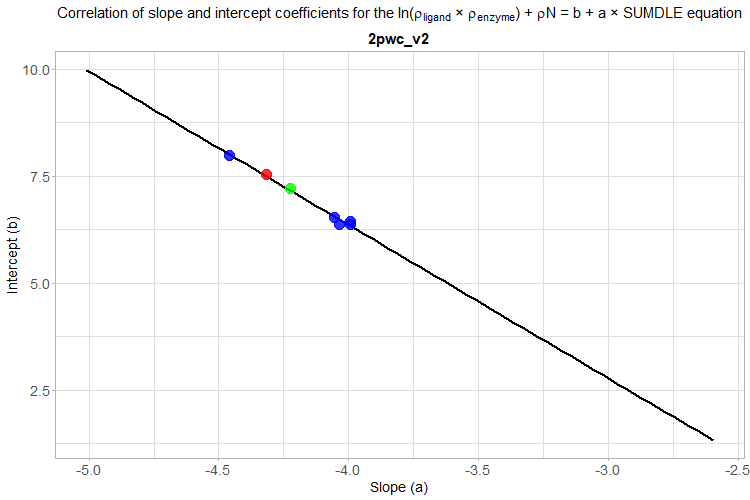

Supplement: Supplementary file 1 — ci0c01382_si_001.zip [file ci0c01382_si_001.zip › Supporting_Information/electron_density_data/HIV_1_protease/Coefficient data/2pwc_v2.png]

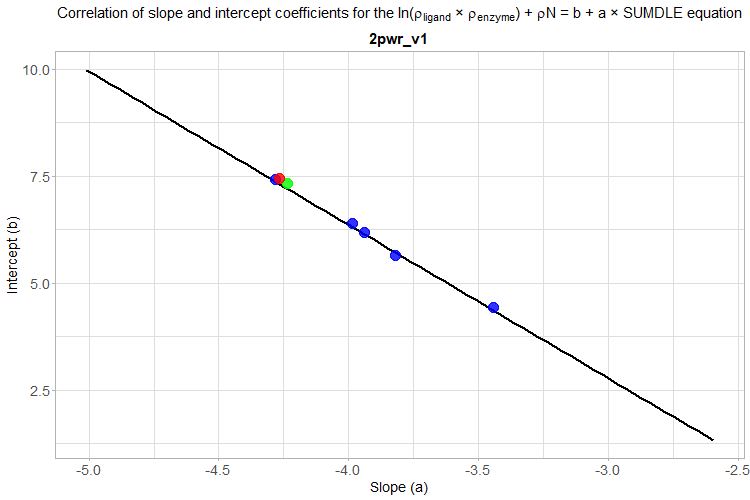

Supplement: Supplementary file 1 — ci0c01382_si_001.zip [file ci0c01382_si_001.zip › Supporting_Information/electron_density_data/HIV_1_protease/Coefficient data/2pwr_v1.png]

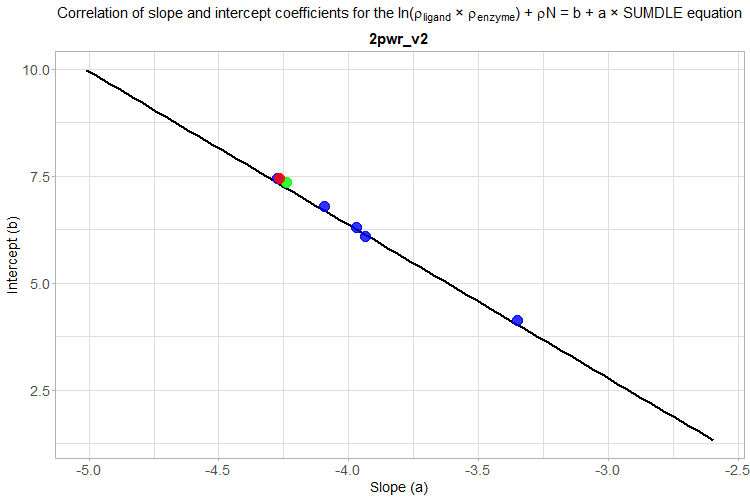

Supplement: Supplementary file 1 — ci0c01382_si_001.zip [file ci0c01382_si_001.zip › Supporting_Information/electron_density_data/HIV_1_protease/Coefficient data/2pwr_v2.png]

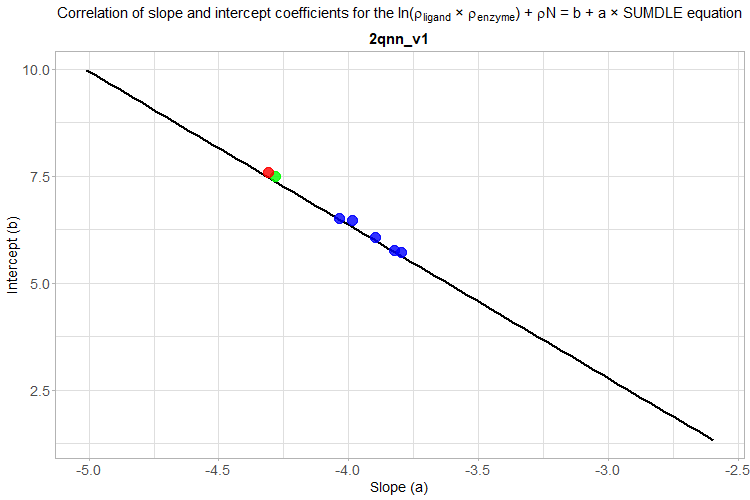

Supplement: Supplementary file 1 — ci0c01382_si_001.zip [file ci0c01382_si_001.zip › Supporting_Information/electron_density_data/HIV_1_protease/Coefficient data/2qnn_v1.png]

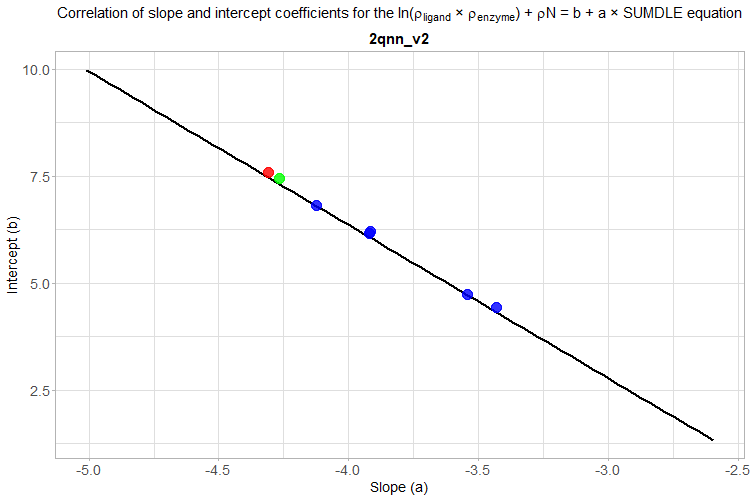

Supplement: Supplementary file 1 — ci0c01382_si_001.zip [file ci0c01382_si_001.zip › Supporting_Information/electron_density_data/HIV_1_protease/Coefficient data/2qnn_v2.png]

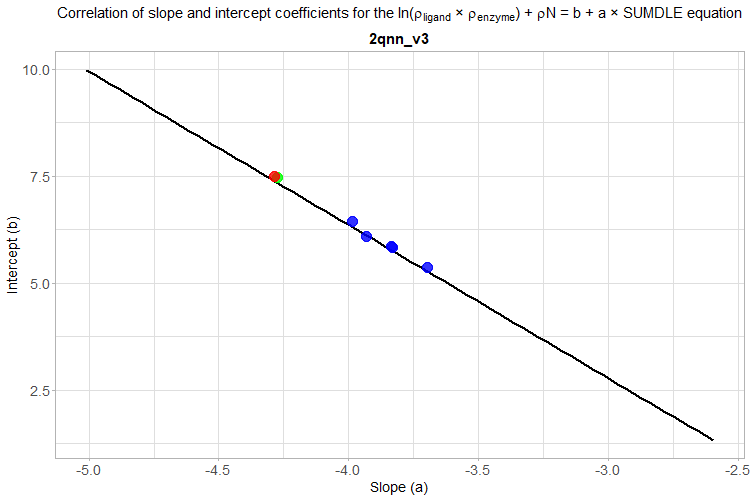

Supplement: Supplementary file 1 — ci0c01382_si_001.zip [file ci0c01382_si_001.zip › Supporting_Information/electron_density_data/HIV_1_protease/Coefficient data/2qnn_v3.png]

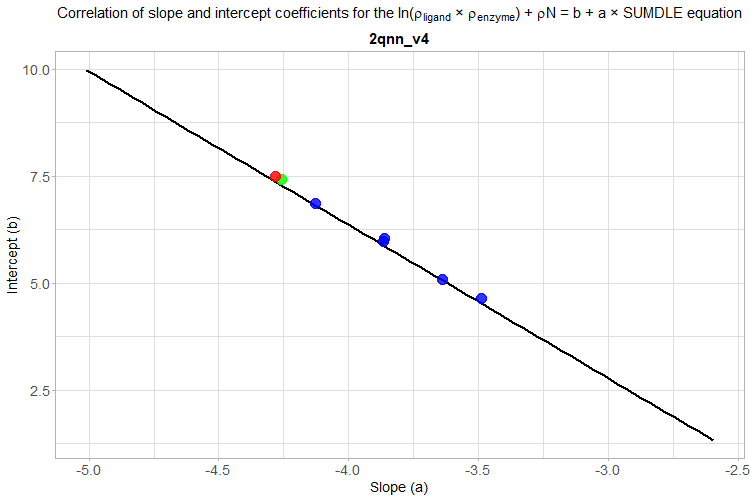

Supplement: Supplementary file 1 — ci0c01382_si_001.zip [file ci0c01382_si_001.zip › Supporting_Information/electron_density_data/HIV_1_protease/Coefficient data/2qnn_v4.png]

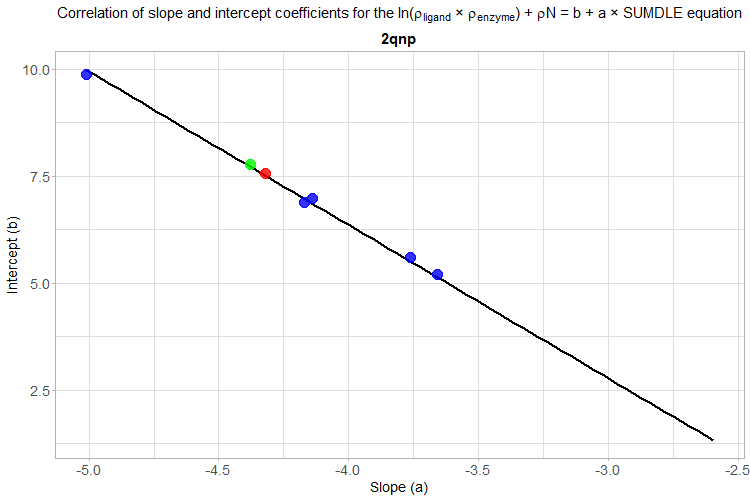

Supplement: Supplementary file 1 — ci0c01382_si_001.zip [file ci0c01382_si_001.zip › Supporting_Information/electron_density_data/HIV_1_protease/Coefficient data/2qnp.png]

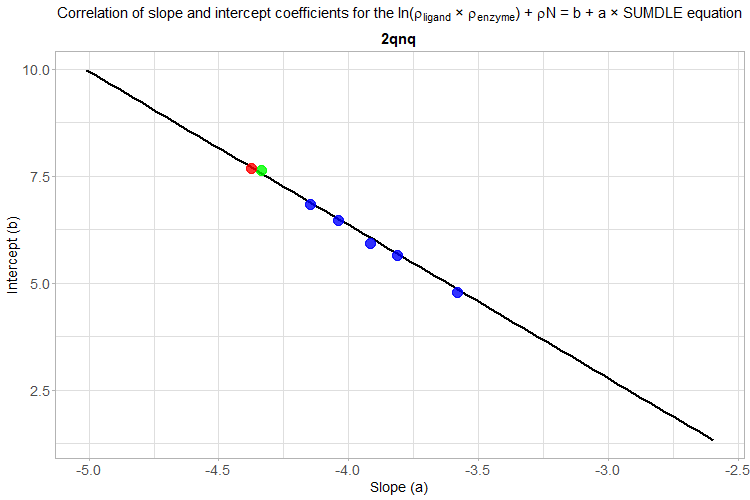

Supplement: Supplementary file 1 — ci0c01382_si_001.zip [file ci0c01382_si_001.zip › Supporting_Information/electron_density_data/HIV_1_protease/Coefficient data/2qnq.png]

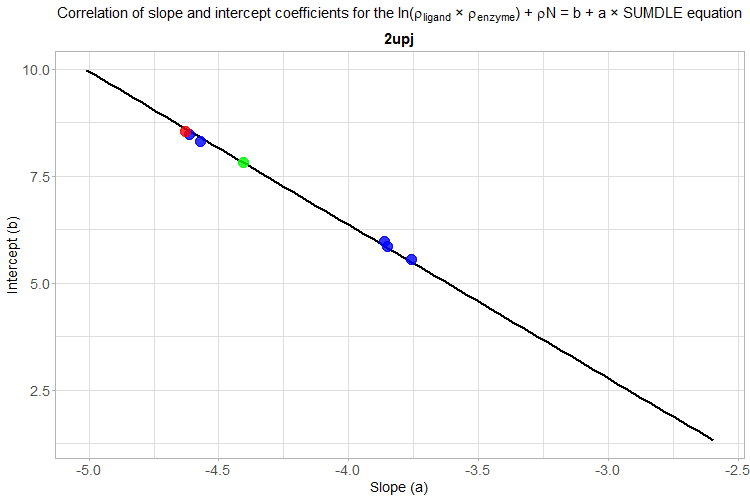

Supplement: Supplementary file 1 — ci0c01382_si_001.zip [file ci0c01382_si_001.zip › Supporting_Information/electron_density_data/HIV_1_protease/Coefficient data/2upj.png]

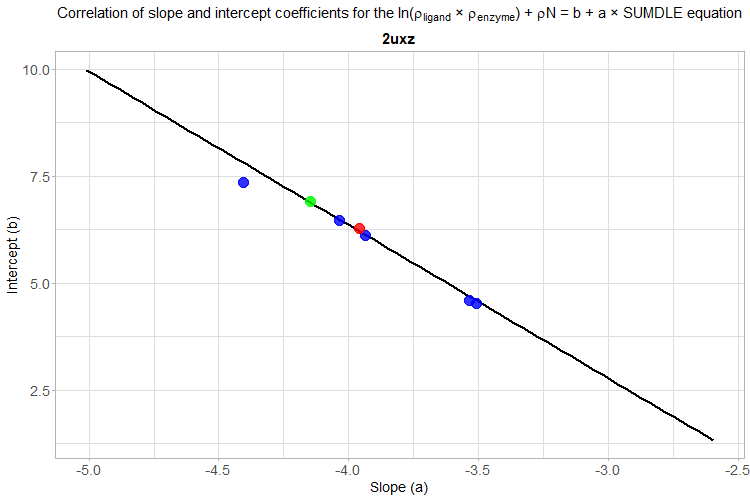

Supplement: Supplementary file 1 — ci0c01382_si_001.zip [file ci0c01382_si_001.zip › Supporting_Information/electron_density_data/HIV_1_protease/Coefficient data/2uxz.png]

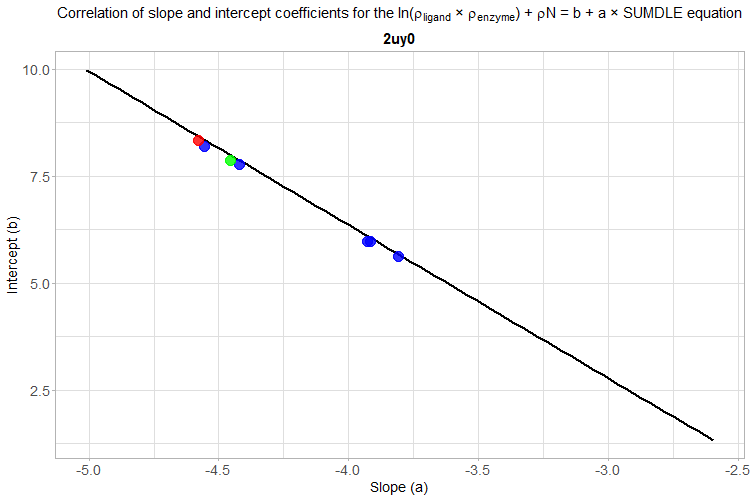

Supplement: Supplementary file 1 — ci0c01382_si_001.zip [file ci0c01382_si_001.zip › Supporting_Information/electron_density_data/HIV_1_protease/Coefficient data/2uy0.png]

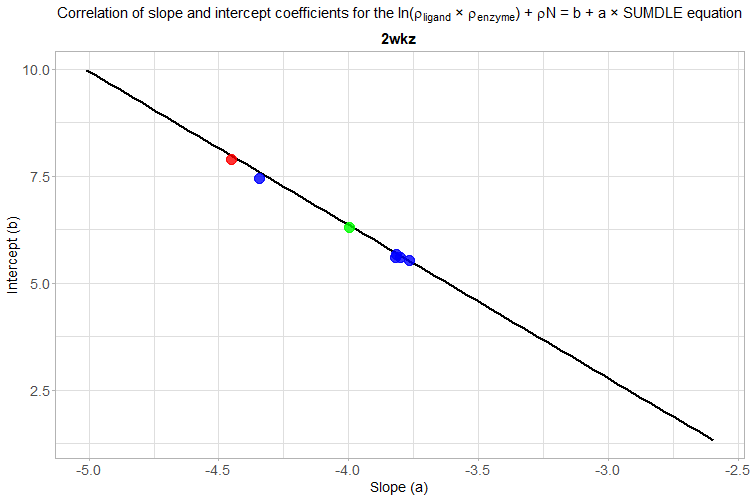

Supplement: Supplementary file 1 — ci0c01382_si_001.zip [file ci0c01382_si_001.zip › Supporting_Information/electron_density_data/HIV_1_protease/Coefficient data/2wkz.png]

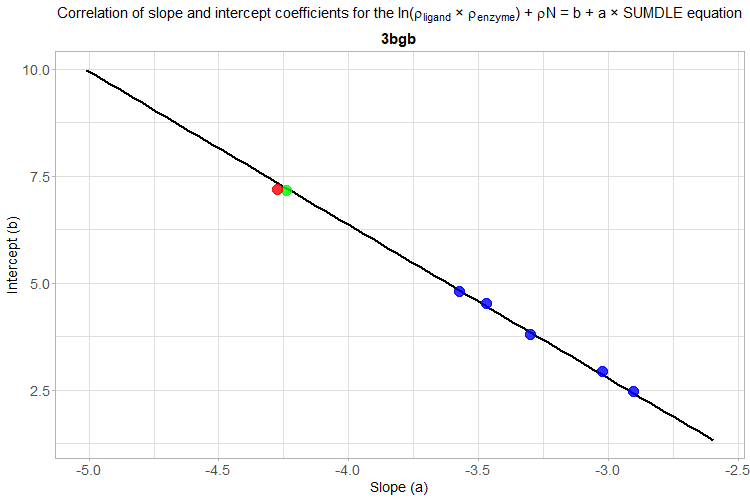

Supplement: Supplementary file 1 — ci0c01382_si_001.zip [file ci0c01382_si_001.zip › Supporting_Information/electron_density_data/HIV_1_protease/Coefficient data/3bgb.png]

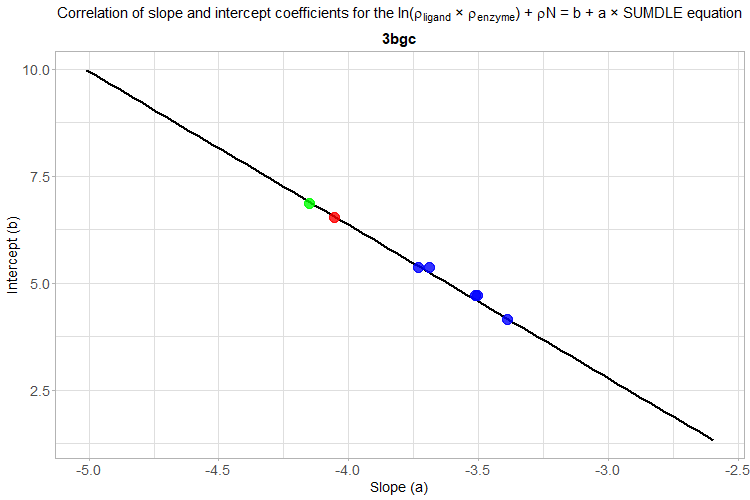

Supplement: Supplementary file 1 — ci0c01382_si_001.zip [file ci0c01382_si_001.zip › Supporting_Information/electron_density_data/HIV_1_protease/Coefficient data/3bgc.png]

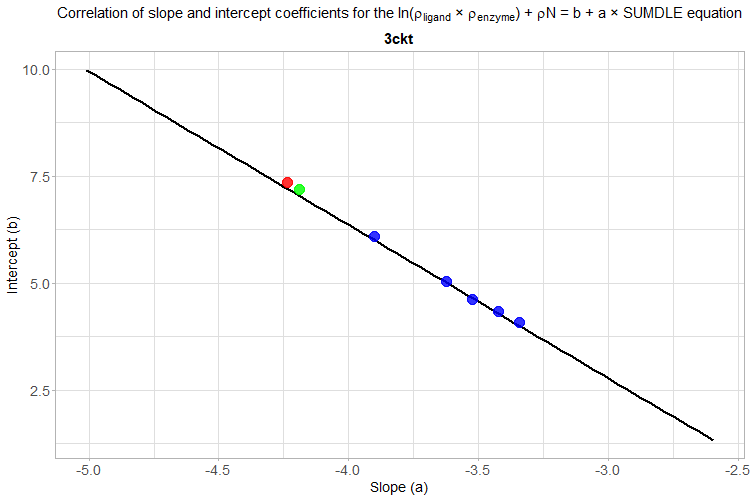

Supplement: Supplementary file 1 — ci0c01382_si_001.zip [file ci0c01382_si_001.zip › Supporting_Information/electron_density_data/HIV_1_protease/Coefficient data/3ckt.png]

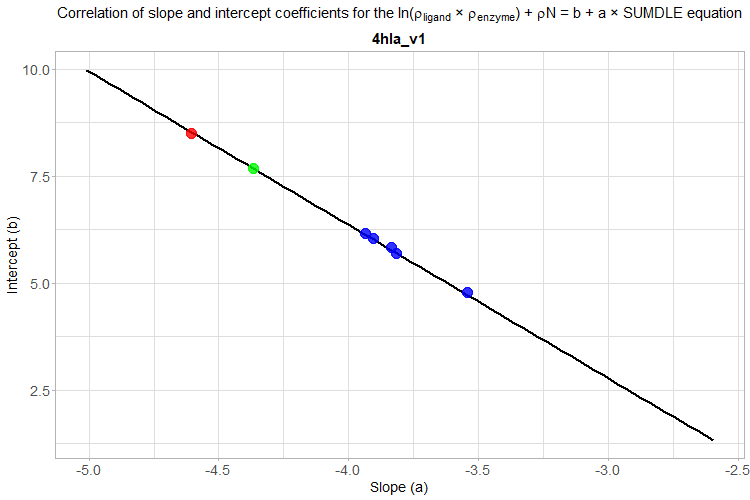

Supplement: Supplementary file 1 — ci0c01382_si_001.zip [file ci0c01382_si_001.zip › Supporting_Information/electron_density_data/HIV_1_protease/Coefficient data/4hla_v1.png]

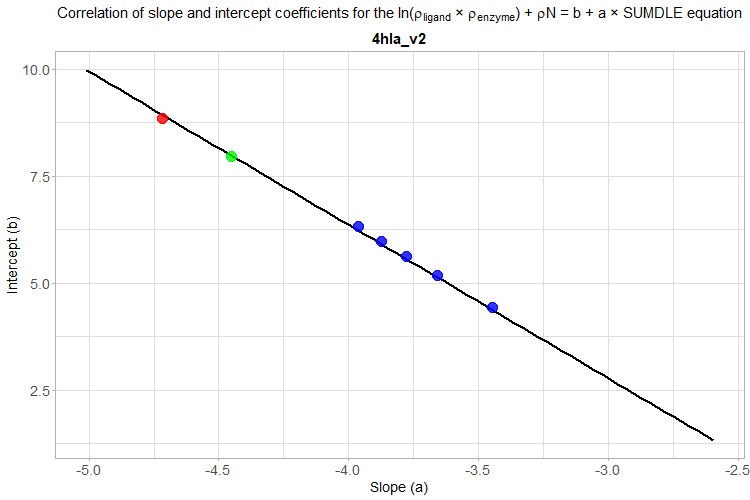

Supplement: Supplementary file 1 — ci0c01382_si_001.zip [file ci0c01382_si_001.zip › Supporting_Information/electron_density_data/HIV_1_protease/Coefficient data/4hla_v2.png]

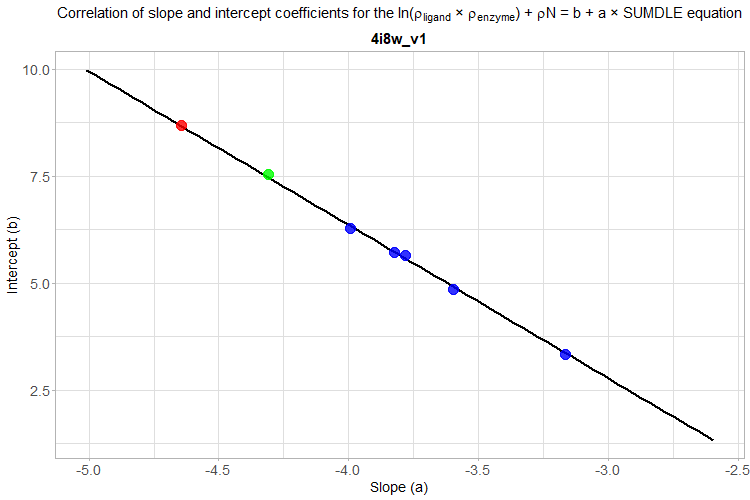

Supplement: Supplementary file 1 — ci0c01382_si_001.zip [file ci0c01382_si_001.zip › Supporting_Information/electron_density_data/HIV_1_protease/Coefficient data/4i8w_v1.png]

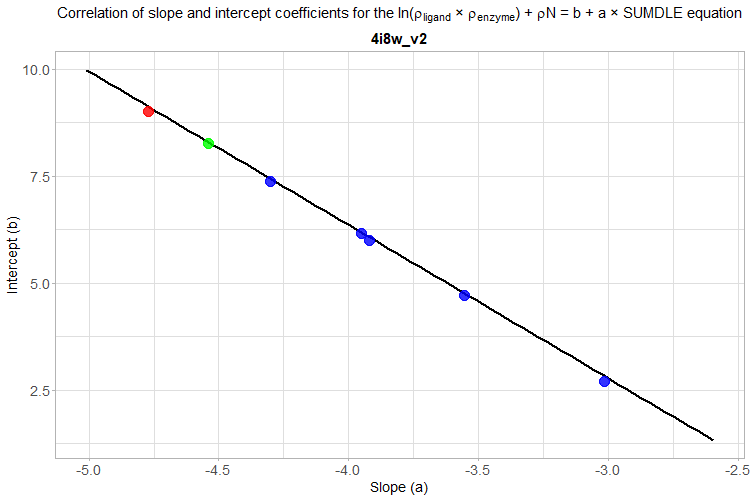

Supplement: Supplementary file 1 — ci0c01382_si_001.zip [file ci0c01382_si_001.zip › Supporting_Information/electron_density_data/HIV_1_protease/Coefficient data/4i8w_v2.png]

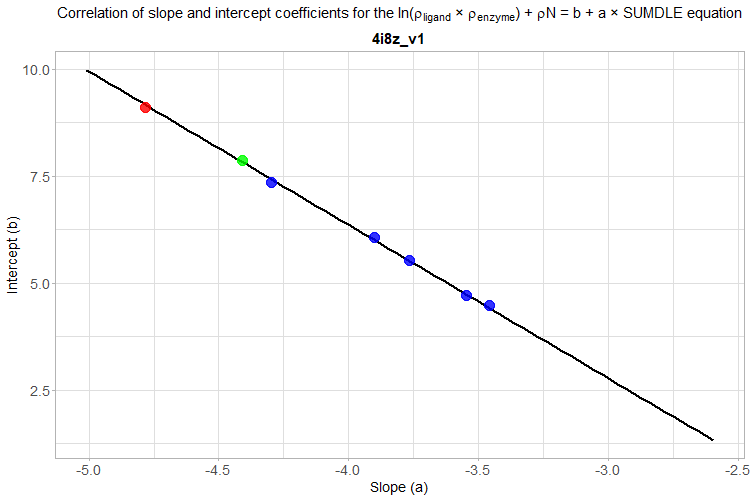

Supplement: Supplementary file 1 — ci0c01382_si_001.zip [file ci0c01382_si_001.zip › Supporting_Information/electron_density_data/HIV_1_protease/Coefficient data/4i8z_v1.png]

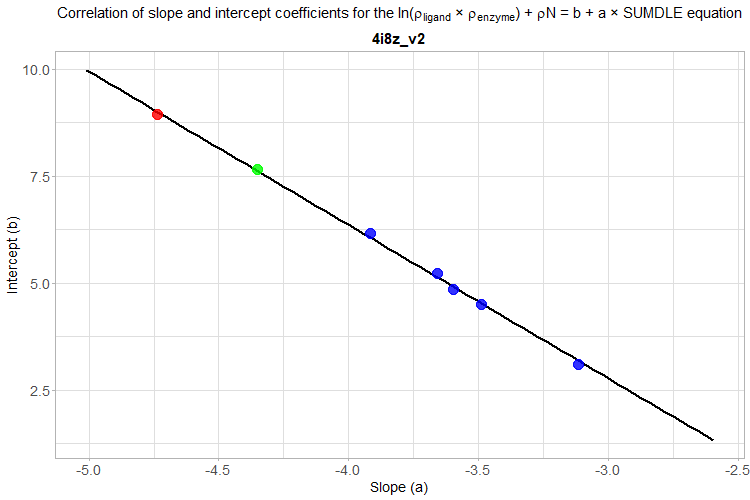

Supplement: Supplementary file 1 — ci0c01382_si_001.zip [file ci0c01382_si_001.zip › Supporting_Information/electron_density_data/HIV_1_protease/Coefficient data/4i8z_v2.png]

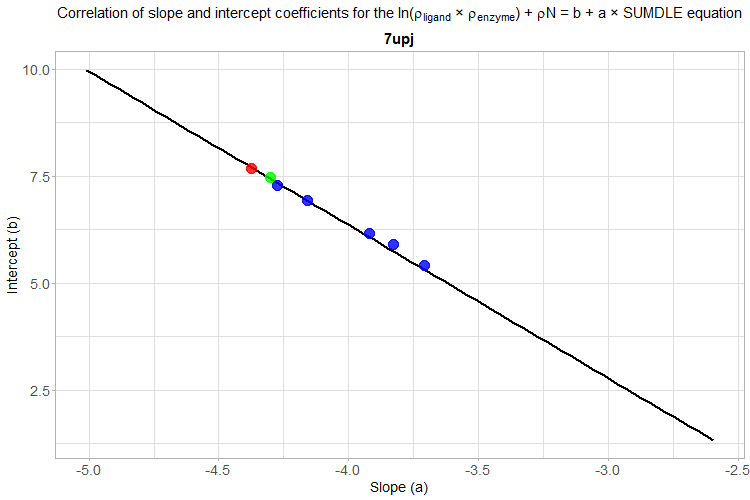

Supplement: Supplementary file 1 — ci0c01382_si_001.zip [file ci0c01382_si_001.zip › Supporting_Information/electron_density_data/HIV_1_protease/Coefficient data/7upj.png]

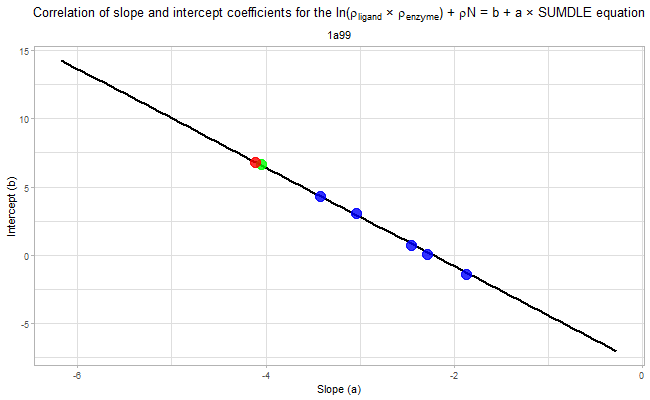

Supplement: Supplementary file 1 — ci0c01382_si_001.zip [file ci0c01382_si_001.zip › Supporting_Information/electron_density_data/PDBbind/Coefficient data/1a99.png]

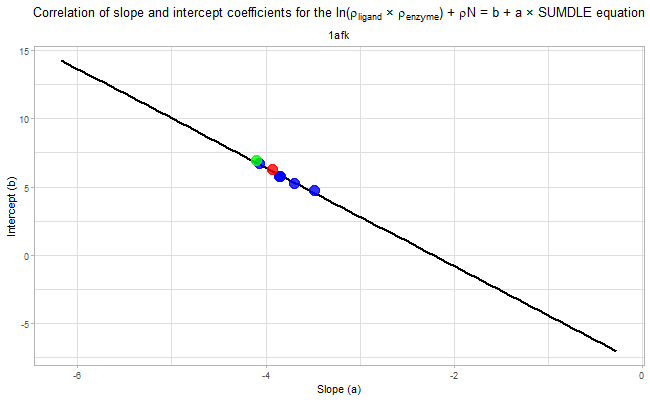

Supplement: Supplementary file 1 — ci0c01382_si_001.zip [file ci0c01382_si_001.zip › Supporting_Information/electron_density_data/PDBbind/Coefficient data/1afk.png]

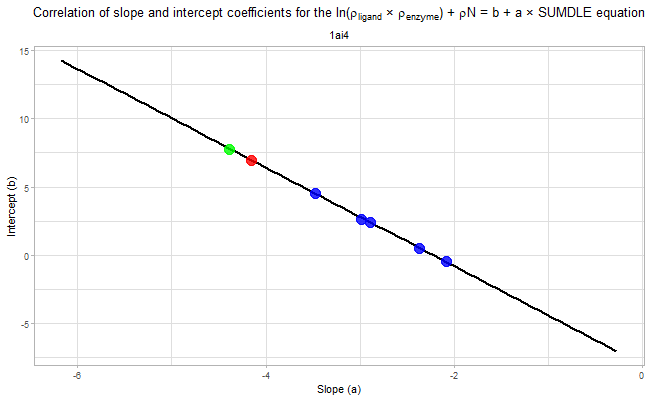

Supplement: Supplementary file 1 — ci0c01382_si_001.zip [file ci0c01382_si_001.zip › Supporting_Information/electron_density_data/PDBbind/Coefficient data/1ai4.png]

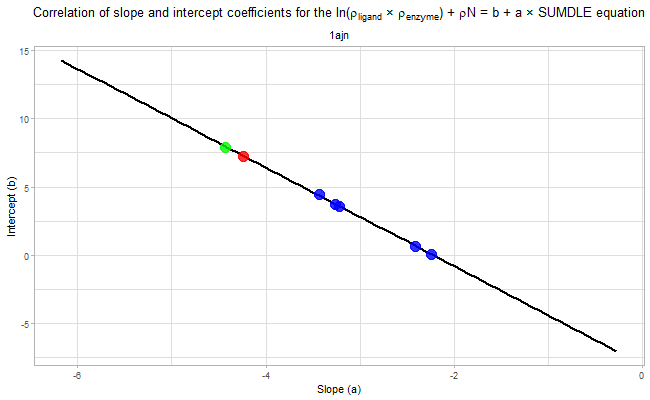

Supplement: Supplementary file 1 — ci0c01382_si_001.zip [file ci0c01382_si_001.zip › Supporting_Information/electron_density_data/PDBbind/Coefficient data/1ajn.png]

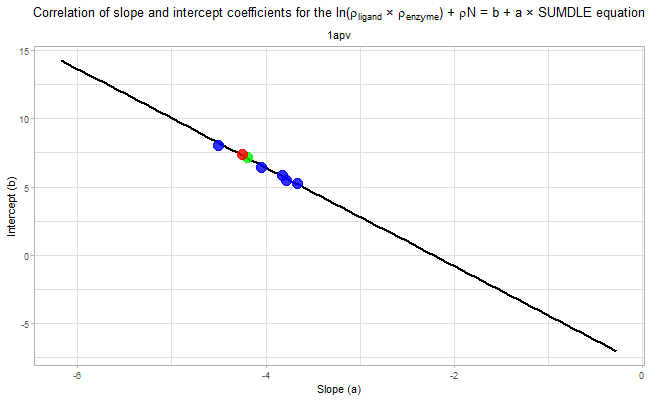

Supplement: Supplementary file 1 — ci0c01382_si_001.zip [file ci0c01382_si_001.zip › Supporting_Information/electron_density_data/PDBbind/Coefficient data/1apv.png]

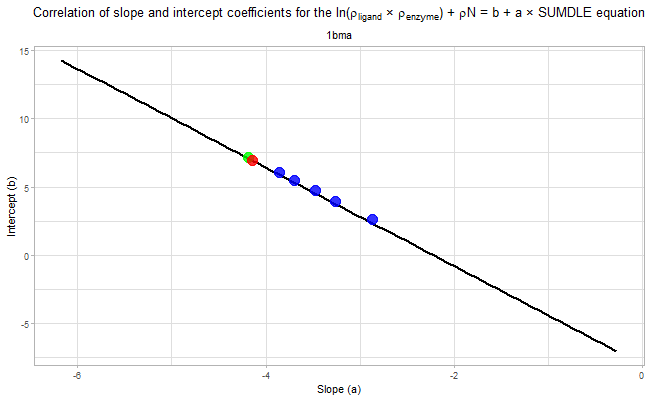

Supplement: Supplementary file 1 — ci0c01382_si_001.zip [file ci0c01382_si_001.zip › Supporting_Information/electron_density_data/PDBbind/Coefficient data/1bma.png]

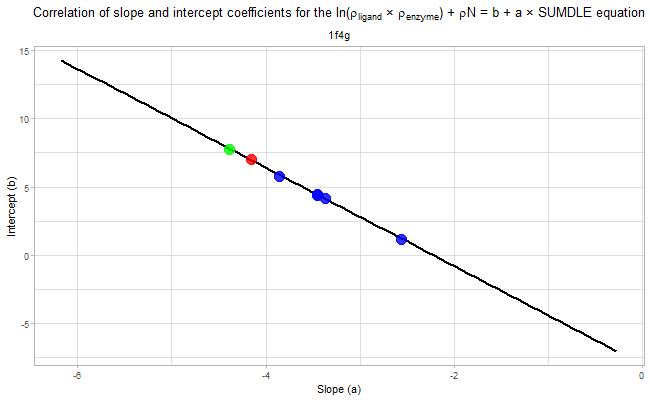

Supplement: Supplementary file 1 — ci0c01382_si_001.zip [file ci0c01382_si_001.zip › Supporting_Information/electron_density_data/PDBbind/Coefficient data/1f4g.png]

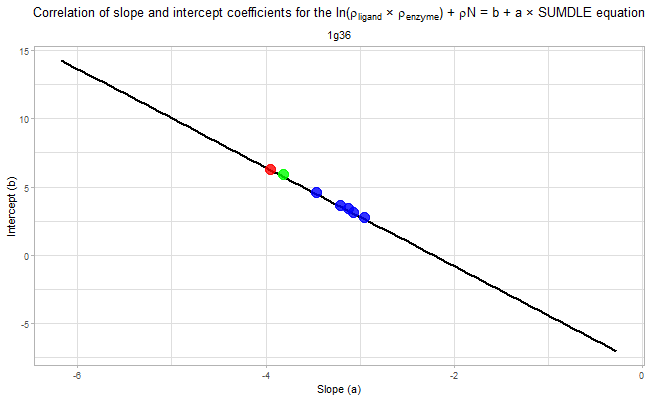

Supplement: Supplementary file 1 — ci0c01382_si_001.zip [file ci0c01382_si_001.zip › Supporting_Information/electron_density_data/PDBbind/Coefficient data/1g36.png]

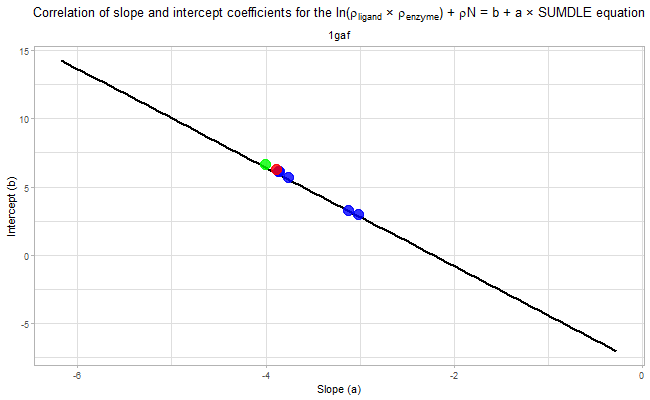

Supplement: Supplementary file 1 — ci0c01382_si_001.zip [file ci0c01382_si_001.zip › Supporting_Information/electron_density_data/PDBbind/Coefficient data/1gaf.png]

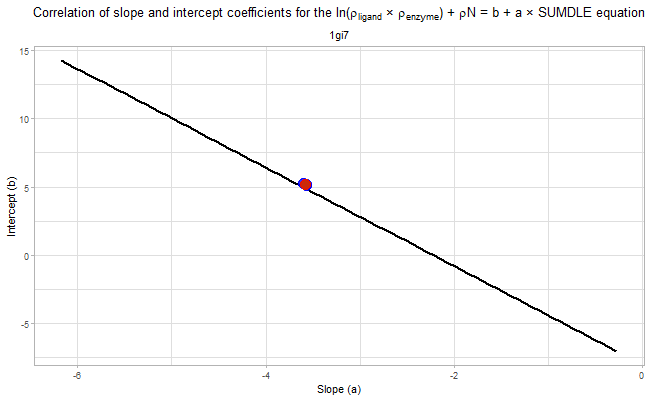

Supplement: Supplementary file 1 — ci0c01382_si_001.zip [file ci0c01382_si_001.zip › Supporting_Information/electron_density_data/PDBbind/Coefficient data/1gi7.png]

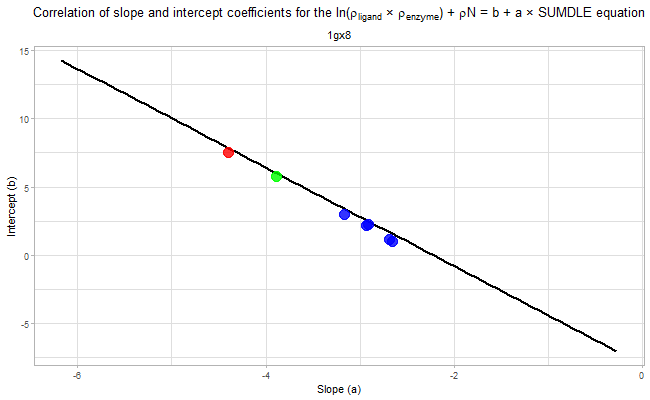

Supplement: Supplementary file 1 — ci0c01382_si_001.zip [file ci0c01382_si_001.zip › Supporting_Information/electron_density_data/PDBbind/Coefficient data/1gx8.png]

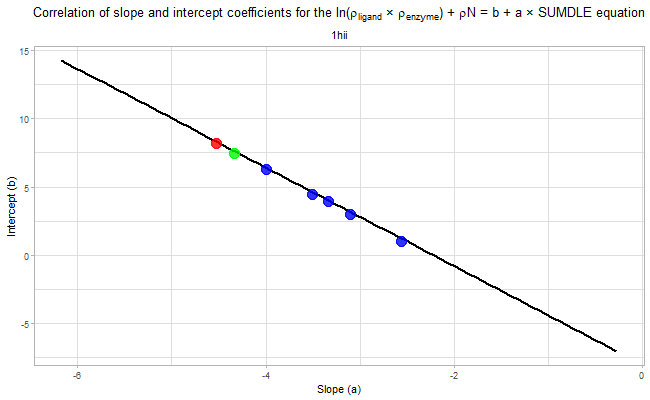

Supplement: Supplementary file 1 — ci0c01382_si_001.zip [file ci0c01382_si_001.zip › Supporting_Information/electron_density_data/PDBbind/Coefficient data/1hii.png]

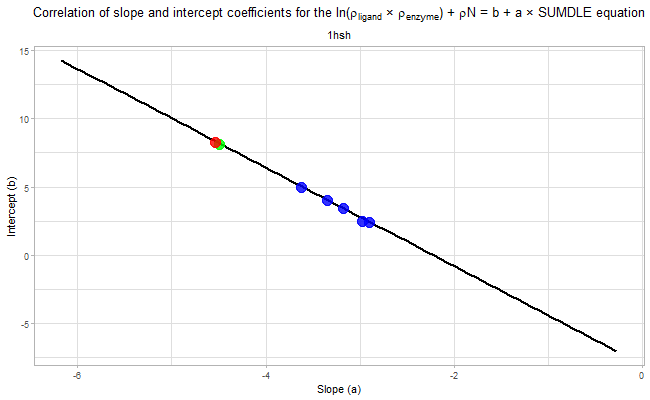

Supplement: Supplementary file 1 — ci0c01382_si_001.zip [file ci0c01382_si_001.zip › Supporting_Information/electron_density_data/PDBbind/Coefficient data/1hsh.png]

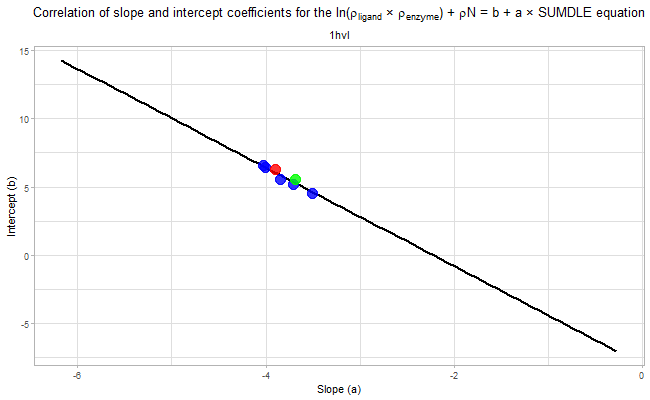

Supplement: Supplementary file 1 — ci0c01382_si_001.zip [file ci0c01382_si_001.zip › Supporting_Information/electron_density_data/PDBbind/Coefficient data/1hvl.png]

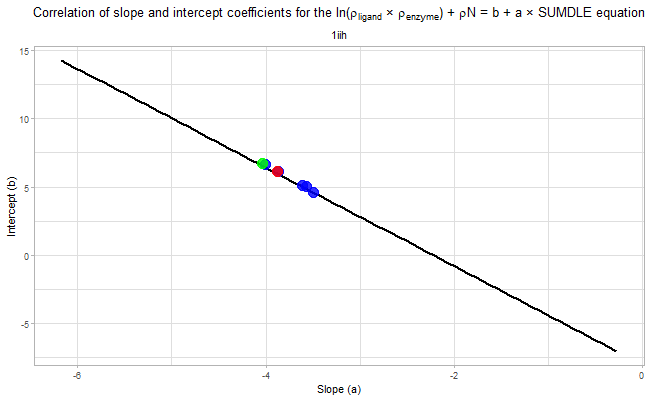

Supplement: Supplementary file 1 — ci0c01382_si_001.zip [file ci0c01382_si_001.zip › Supporting_Information/electron_density_data/PDBbind/Coefficient data/1iih.png]

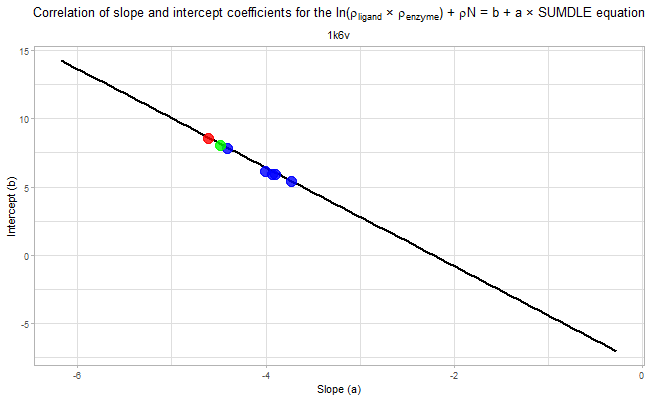

Supplement: Supplementary file 1 — ci0c01382_si_001.zip [file ci0c01382_si_001.zip › Supporting_Information/electron_density_data/PDBbind/Coefficient data/1k6v.png]

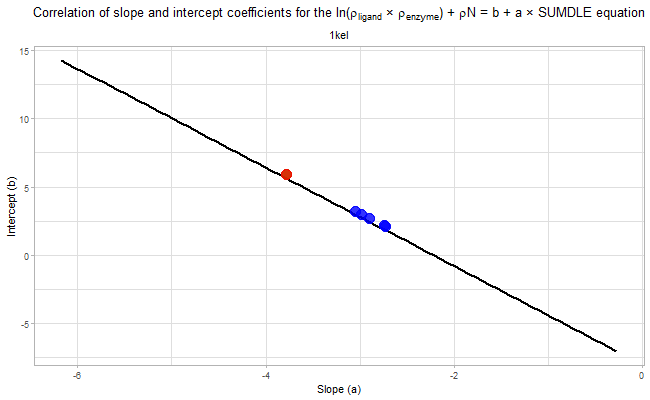

Supplement: Supplementary file 1 — ci0c01382_si_001.zip [file ci0c01382_si_001.zip › Supporting_Information/electron_density_data/PDBbind/Coefficient data/1kel.png]

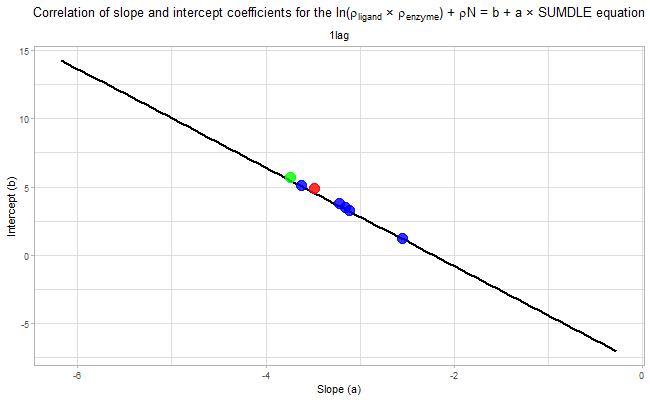

Supplement: Supplementary file 1 — ci0c01382_si_001.zip [file ci0c01382_si_001.zip › Supporting_Information/electron_density_data/PDBbind/Coefficient data/1lag.png]

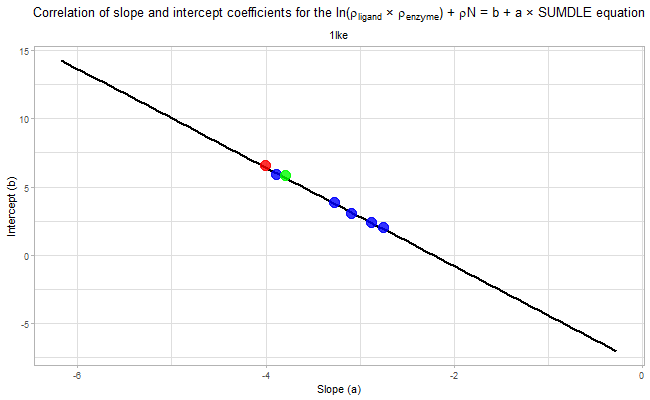

Supplement: Supplementary file 1 — ci0c01382_si_001.zip [file ci0c01382_si_001.zip › Supporting_Information/electron_density_data/PDBbind/Coefficient data/1lke.png]

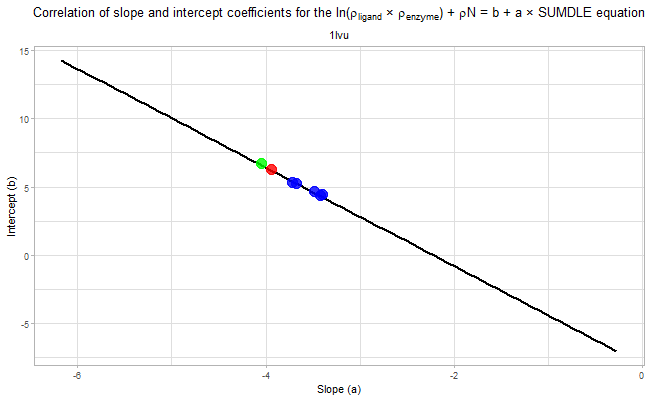

Supplement: Supplementary file 1 — ci0c01382_si_001.zip [file ci0c01382_si_001.zip › Supporting_Information/electron_density_data/PDBbind/Coefficient data/1lvu.png]

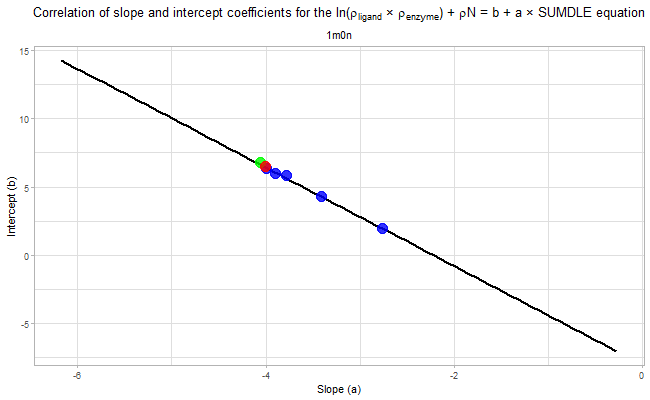

Supplement: Supplementary file 1 — ci0c01382_si_001.zip [file ci0c01382_si_001.zip › Supporting_Information/electron_density_data/PDBbind/Coefficient data/1m0n.png]

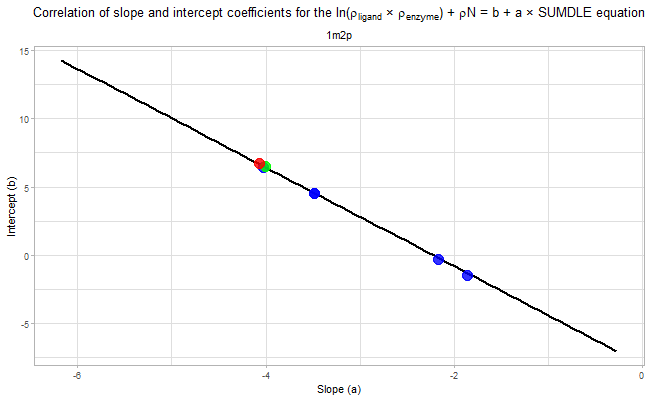

Supplement: Supplementary file 1 — ci0c01382_si_001.zip [file ci0c01382_si_001.zip › Supporting_Information/electron_density_data/PDBbind/Coefficient data/1m2p.png]

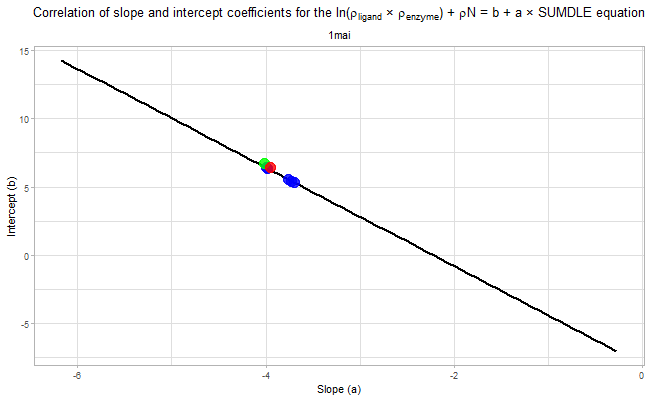

Supplement: Supplementary file 1 — ci0c01382_si_001.zip [file ci0c01382_si_001.zip › Supporting_Information/electron_density_data/PDBbind/Coefficient data/1mai.png]

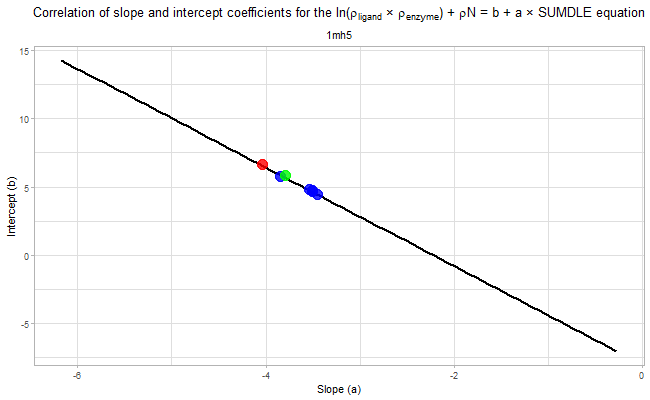

Supplement: Supplementary file 1 — ci0c01382_si_001.zip [file ci0c01382_si_001.zip › Supporting_Information/electron_density_data/PDBbind/Coefficient data/1mh5.png]

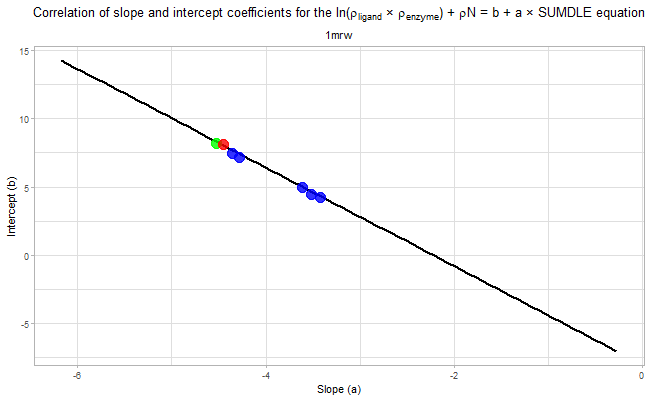

Supplement: Supplementary file 1 — ci0c01382_si_001.zip [file ci0c01382_si_001.zip › Supporting_Information/electron_density_data/PDBbind/Coefficient data/1mrw.png]

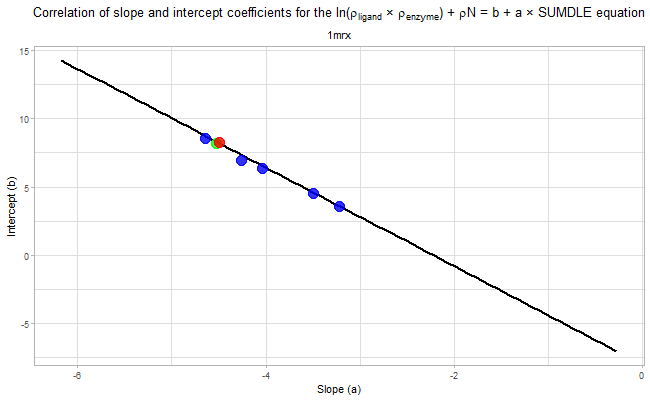

Supplement: Supplementary file 1 — ci0c01382_si_001.zip [file ci0c01382_si_001.zip › Supporting_Information/electron_density_data/PDBbind/Coefficient data/1mrx.png]

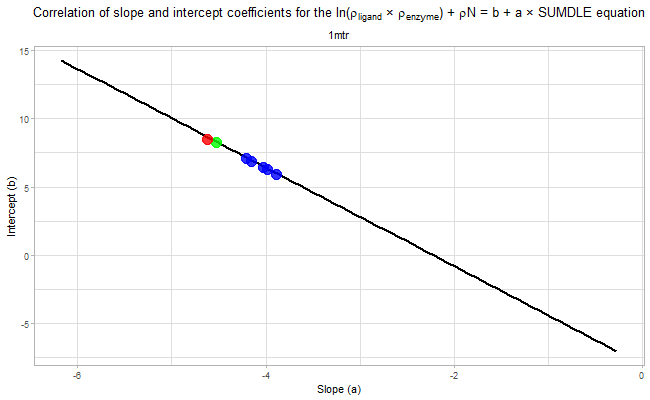

Supplement: Supplementary file 1 — ci0c01382_si_001.zip [file ci0c01382_si_001.zip › Supporting_Information/electron_density_data/PDBbind/Coefficient data/1mtr.png]

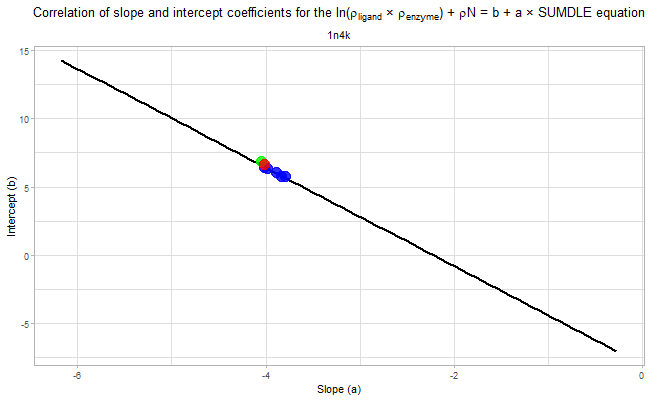

Supplement: Supplementary file 1 — ci0c01382_si_001.zip [file ci0c01382_si_001.zip › Supporting_Information/electron_density_data/PDBbind/Coefficient data/1n4k.png]

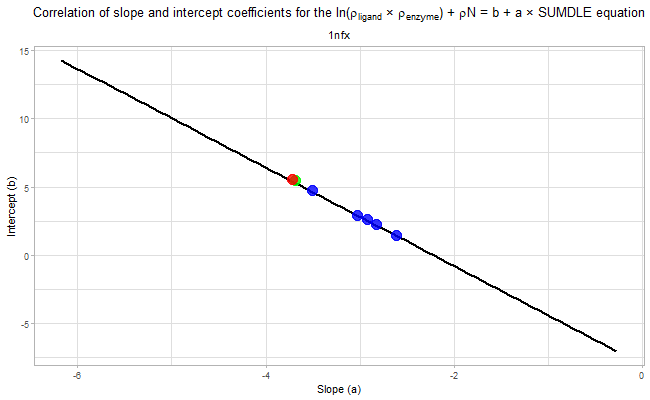

Supplement: Supplementary file 1 — ci0c01382_si_001.zip [file ci0c01382_si_001.zip › Supporting_Information/electron_density_data/PDBbind/Coefficient data/1nfx.png]

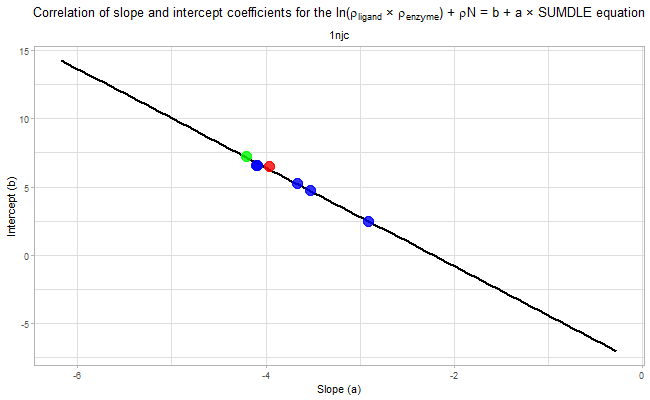

Supplement: Supplementary file 1 — ci0c01382_si_001.zip [file ci0c01382_si_001.zip › Supporting_Information/electron_density_data/PDBbind/Coefficient data/1njc.png]

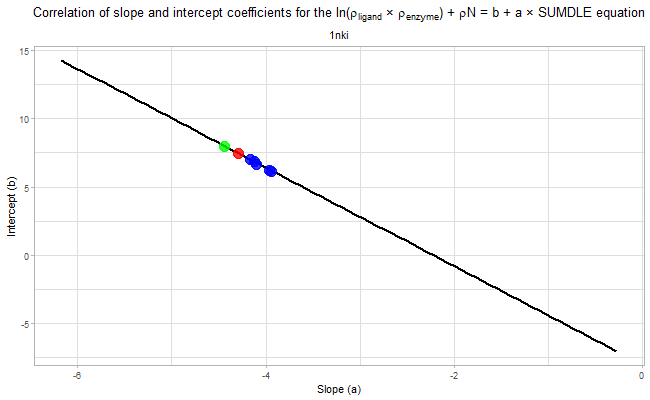

Supplement: Supplementary file 1 — ci0c01382_si_001.zip [file ci0c01382_si_001.zip › Supporting_Information/electron_density_data/PDBbind/Coefficient data/1nki.png]

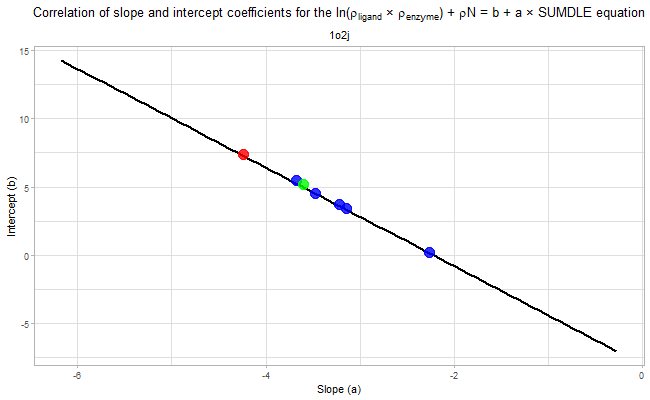

Supplement: Supplementary file 1 — ci0c01382_si_001.zip [file ci0c01382_si_001.zip › Supporting_Information/electron_density_data/PDBbind/Coefficient data/1o2j.png]

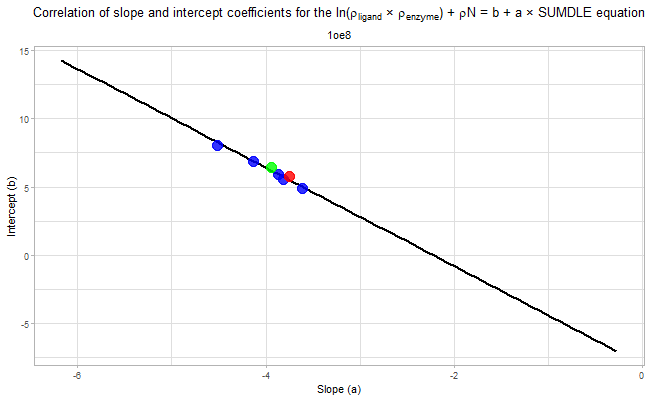

Supplement: Supplementary file 1 — ci0c01382_si_001.zip [file ci0c01382_si_001.zip › Supporting_Information/electron_density_data/PDBbind/Coefficient data/1oe8.png]

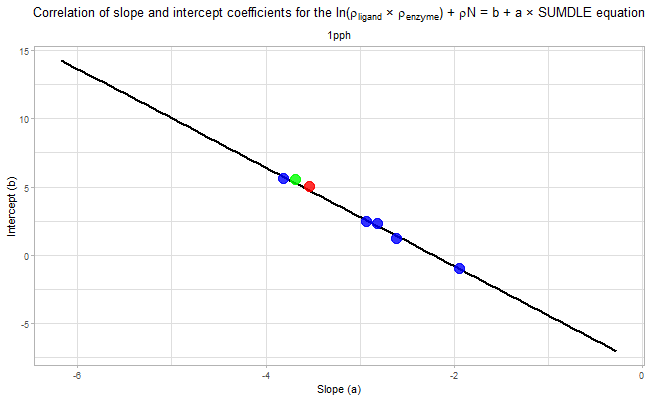

Supplement: Supplementary file 1 — ci0c01382_si_001.zip [file ci0c01382_si_001.zip › Supporting_Information/electron_density_data/PDBbind/Coefficient data/1pph.png]

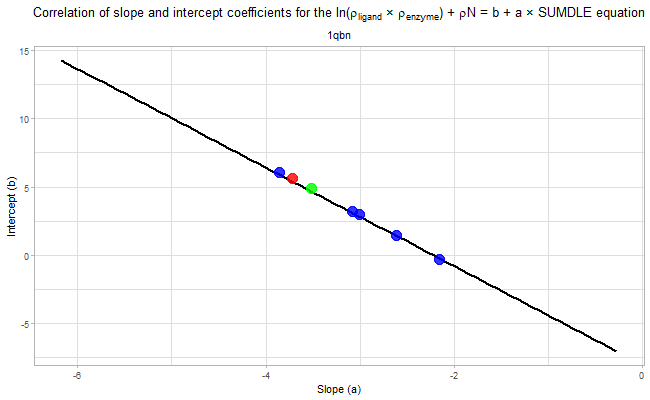

Supplement: Supplementary file 1 — ci0c01382_si_001.zip [file ci0c01382_si_001.zip › Supporting_Information/electron_density_data/PDBbind/Coefficient data/1qbn.png]

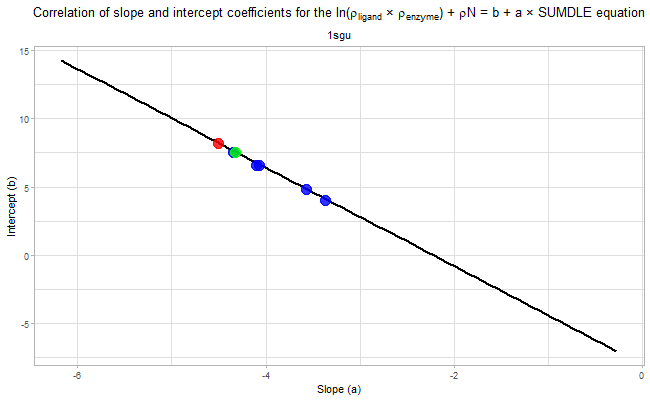

Supplement: Supplementary file 1 — ci0c01382_si_001.zip [file ci0c01382_si_001.zip › Supporting_Information/electron_density_data/PDBbind/Coefficient data/1sgu.png]

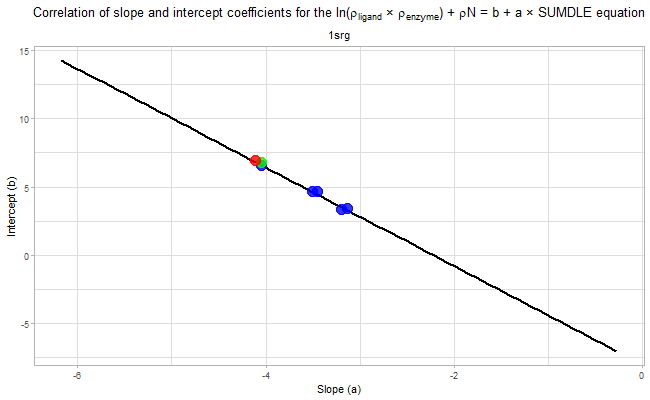

Supplement: Supplementary file 1 — ci0c01382_si_001.zip [file ci0c01382_si_001.zip › Supporting_Information/electron_density_data/PDBbind/Coefficient data/1srg.png]

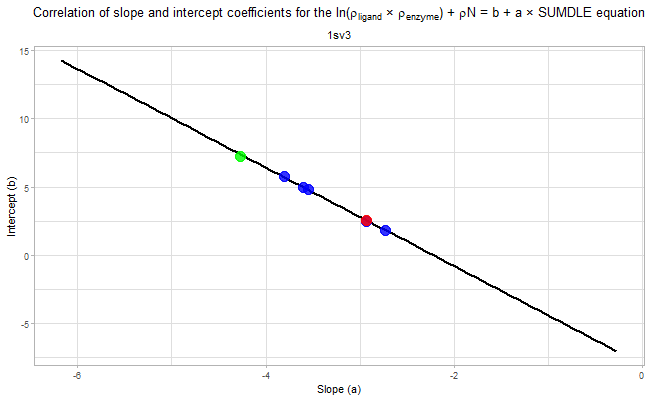

Supplement: Supplementary file 1 — ci0c01382_si_001.zip [file ci0c01382_si_001.zip › Supporting_Information/electron_density_data/PDBbind/Coefficient data/1sv3.png]

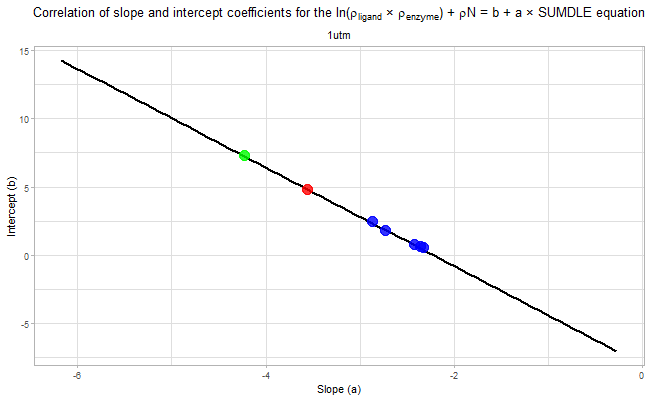

Supplement: Supplementary file 1 — ci0c01382_si_001.zip [file ci0c01382_si_001.zip › Supporting_Information/electron_density_data/PDBbind/Coefficient data/1utm.png]

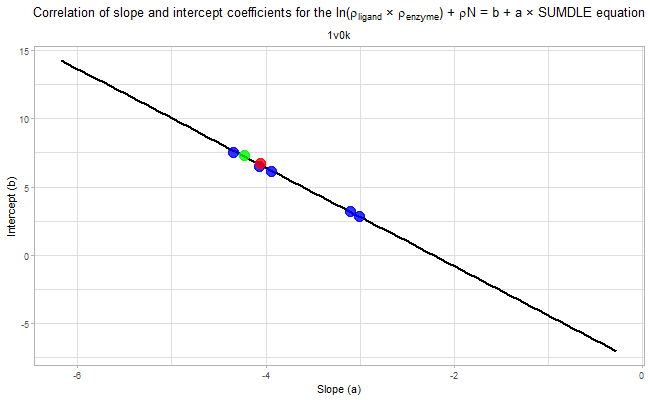

Supplement: Supplementary file 1 — ci0c01382_si_001.zip [file ci0c01382_si_001.zip › Supporting_Information/electron_density_data/PDBbind/Coefficient data/1v0k.png]

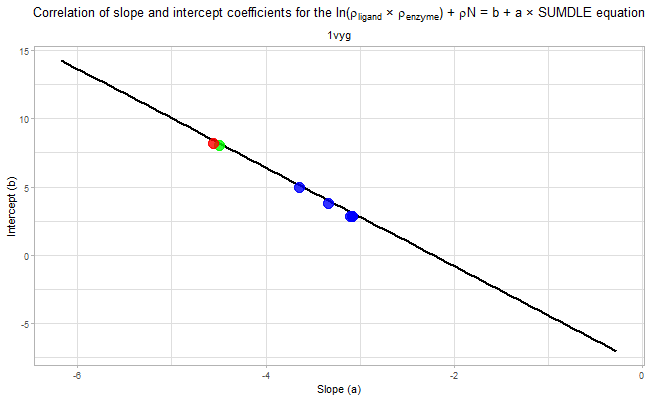

Supplement: Supplementary file 1 — ci0c01382_si_001.zip [file ci0c01382_si_001.zip › Supporting_Information/electron_density_data/PDBbind/Coefficient data/1vyg.png]

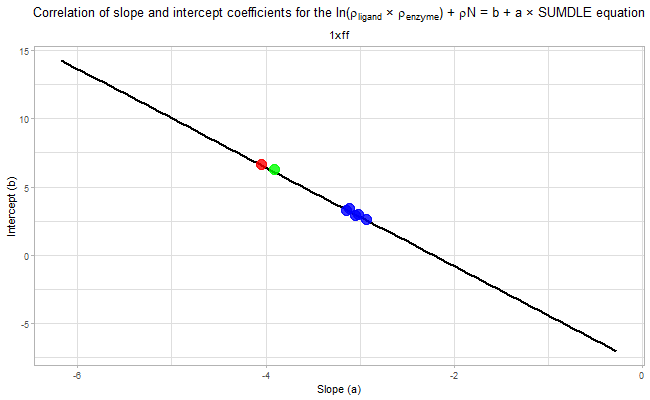

Supplement: Supplementary file 1 — ci0c01382_si_001.zip [file ci0c01382_si_001.zip › Supporting_Information/electron_density_data/PDBbind/Coefficient data/1xff.png]

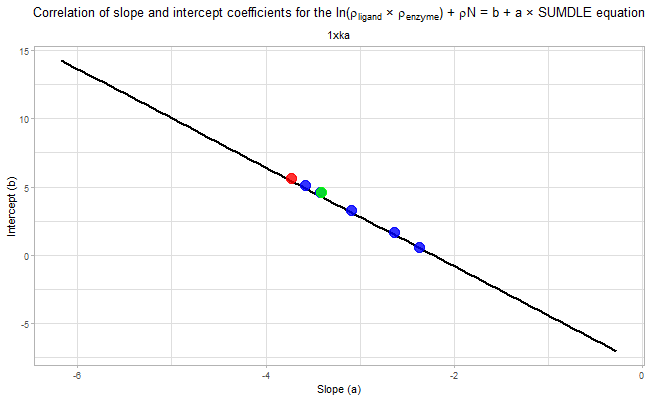

Supplement: Supplementary file 1 — ci0c01382_si_001.zip [file ci0c01382_si_001.zip › Supporting_Information/electron_density_data/PDBbind/Coefficient data/1xka.png]

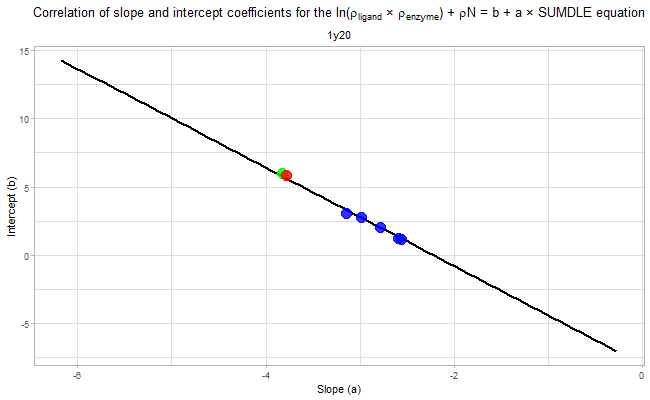

Supplement: Supplementary file 1 — ci0c01382_si_001.zip [file ci0c01382_si_001.zip › Supporting_Information/electron_density_data/PDBbind/Coefficient data/1y20.png]

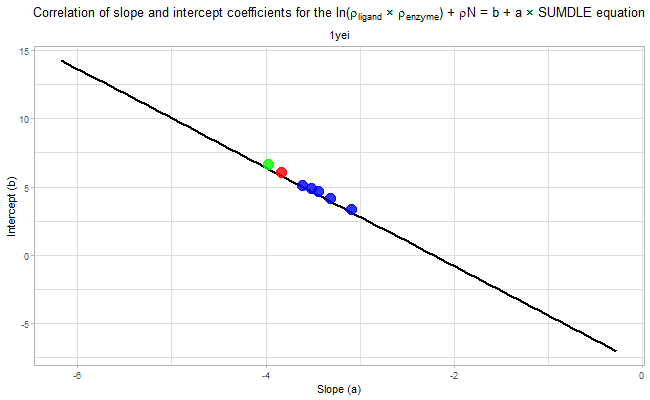

Supplement: Supplementary file 1 — ci0c01382_si_001.zip [file ci0c01382_si_001.zip › Supporting_Information/electron_density_data/PDBbind/Coefficient data/1yei.png]

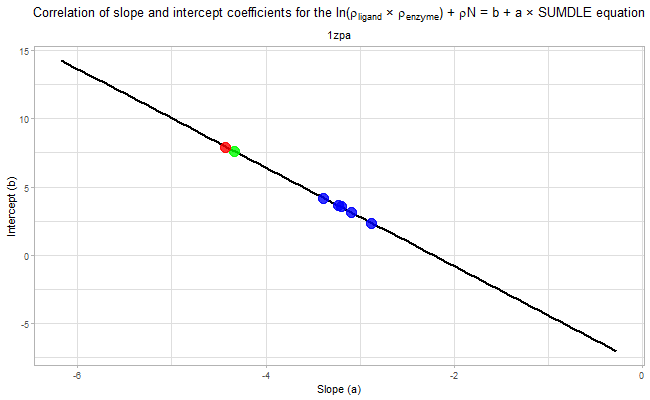

Supplement: Supplementary file 1 — ci0c01382_si_001.zip [file ci0c01382_si_001.zip › Supporting_Information/electron_density_data/PDBbind/Coefficient data/1zpa.png]

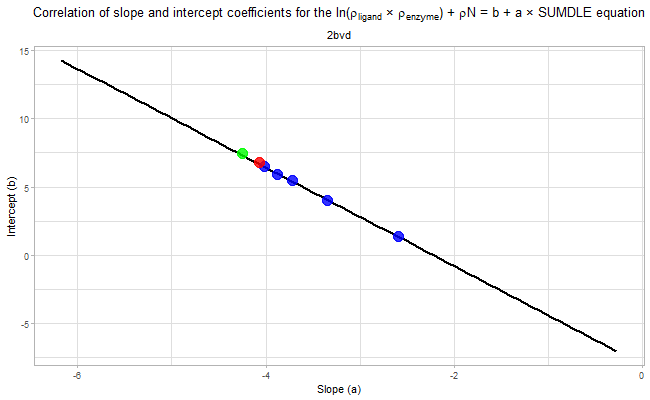

Supplement: Supplementary file 1 — ci0c01382_si_001.zip [file ci0c01382_si_001.zip › Supporting_Information/electron_density_data/PDBbind/Coefficient data/2bvd.png]

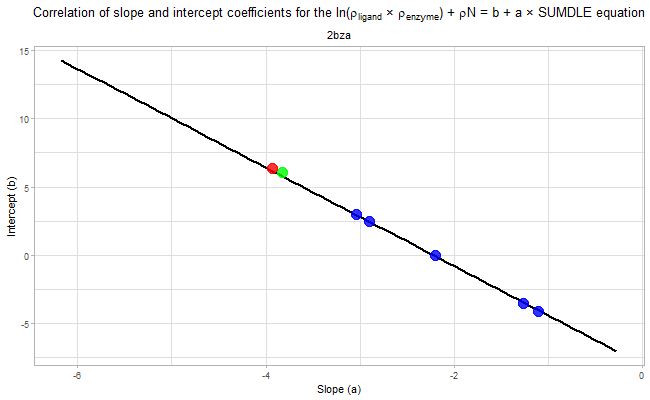

Supplement: Supplementary file 1 — ci0c01382_si_001.zip [file ci0c01382_si_001.zip › Supporting_Information/electron_density_data/PDBbind/Coefficient data/2bza.png]

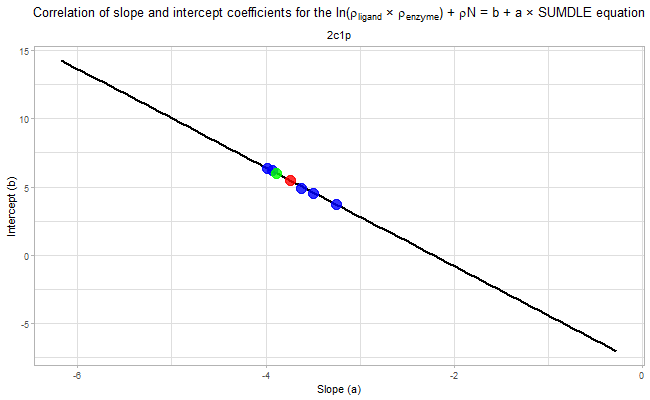

Supplement: Supplementary file 1 — ci0c01382_si_001.zip [file ci0c01382_si_001.zip › Supporting_Information/electron_density_data/PDBbind/Coefficient data/2c1p.png]

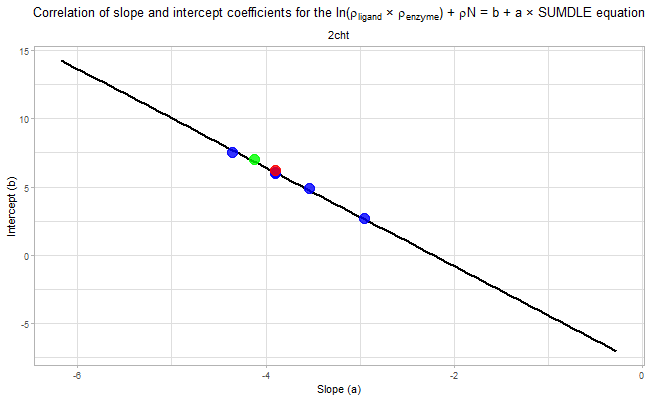

Supplement: Supplementary file 1 — ci0c01382_si_001.zip [file ci0c01382_si_001.zip › Supporting_Information/electron_density_data/PDBbind/Coefficient data/2cht.png]

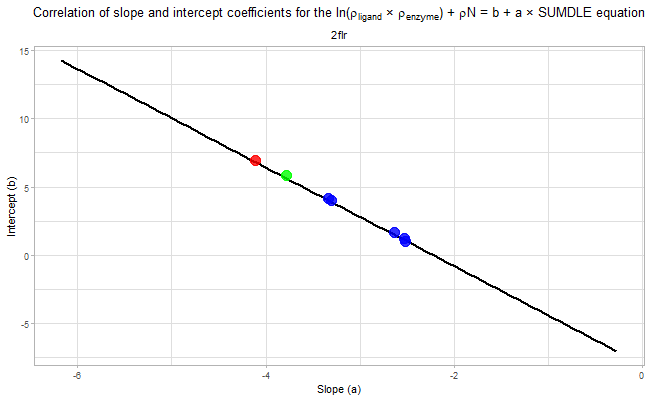

Supplement: Supplementary file 1 — ci0c01382_si_001.zip [file ci0c01382_si_001.zip › Supporting_Information/electron_density_data/PDBbind/Coefficient data/2flr.png]

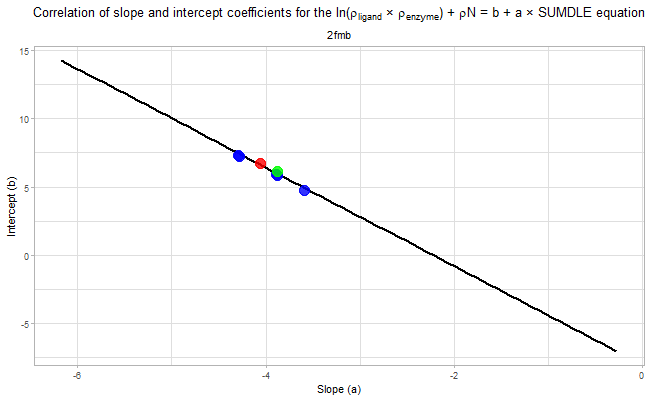

Supplement: Supplementary file 1 — ci0c01382_si_001.zip [file ci0c01382_si_001.zip › Supporting_Information/electron_density_data/PDBbind/Coefficient data/2fmb.png]

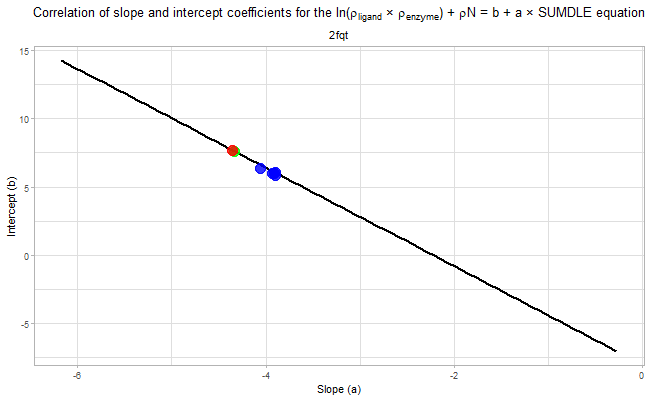

Supplement: Supplementary file 1 — ci0c01382_si_001.zip [file ci0c01382_si_001.zip › Supporting_Information/electron_density_data/PDBbind/Coefficient data/2fqt.png]
