# Supplementary figures and images for: Use of the Complementarity Principle in Docking Procedures: A New Approach for Evaluating the Correctness of Binding Poses (part 1 of 2)
Source: J Chem Inf Model. 2021 Apr 2;61(4):1801–13. doi: 10.1021/acs.jcim.0c01382 (PMC8154257; doi:10.1021/acs.jcim.0c01382)

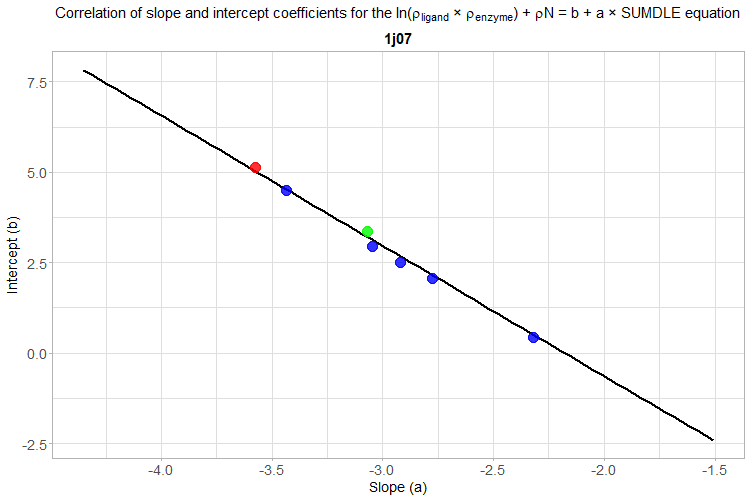

Supplement: Supplementary file 1 — ci0c01382_si_001.zip [file ci0c01382_si_001.zip › Supporting_Information/electron_density_data/ace_chol_esterase/Coefficient data/1j07.png]

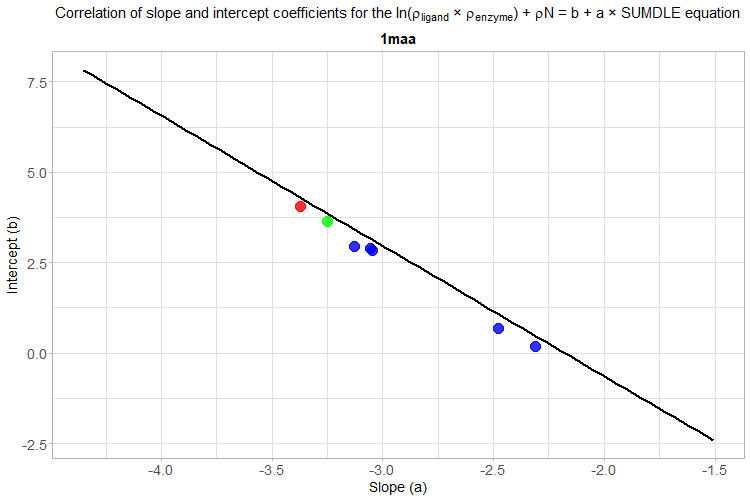

Supplement: Supplementary file 1 — ci0c01382_si_001.zip [file ci0c01382_si_001.zip › Supporting_Information/electron_density_data/ace_chol_esterase/Coefficient data/1maa.png]

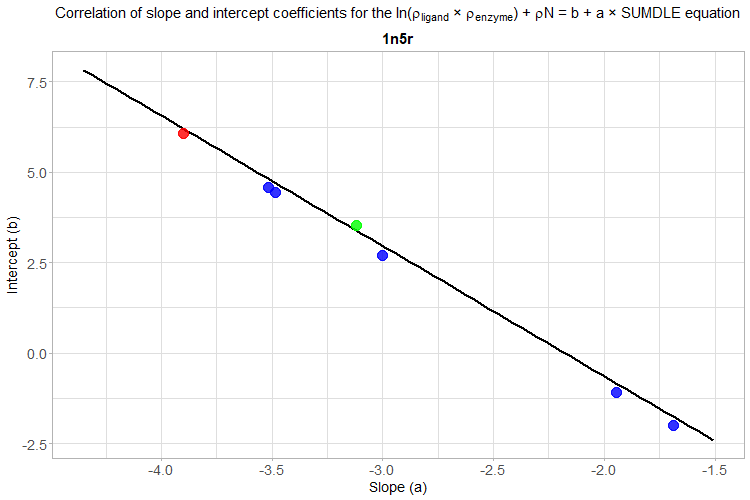

Supplement: Supplementary file 1 — ci0c01382_si_001.zip [file ci0c01382_si_001.zip › Supporting_Information/electron_density_data/ace_chol_esterase/Coefficient data/1n5r.png]

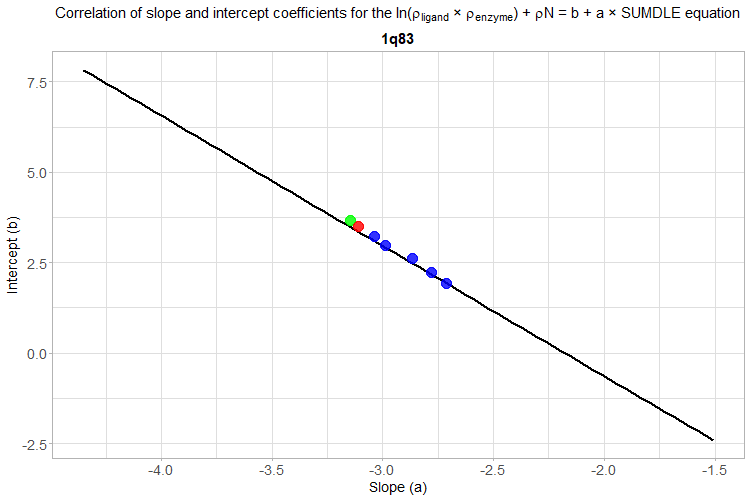

Supplement: Supplementary file 1 — ci0c01382_si_001.zip [file ci0c01382_si_001.zip › Supporting_Information/electron_density_data/ace_chol_esterase/Coefficient data/1q83.png]

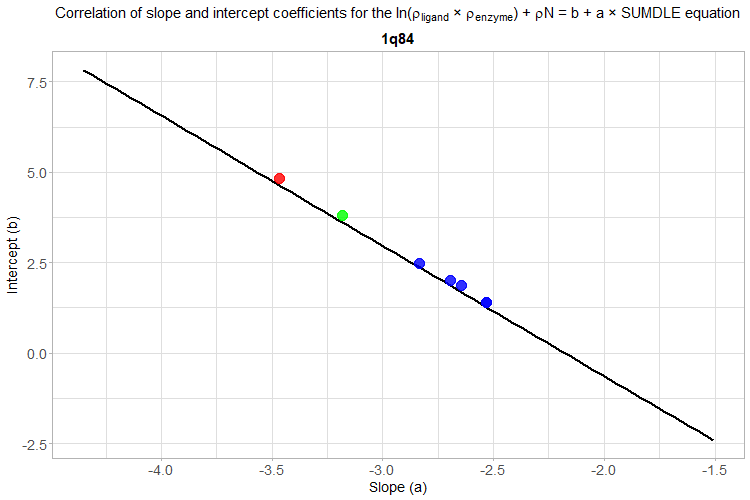

Supplement: Supplementary file 1 — ci0c01382_si_001.zip [file ci0c01382_si_001.zip › Supporting_Information/electron_density_data/ace_chol_esterase/Coefficient data/1q84.png]

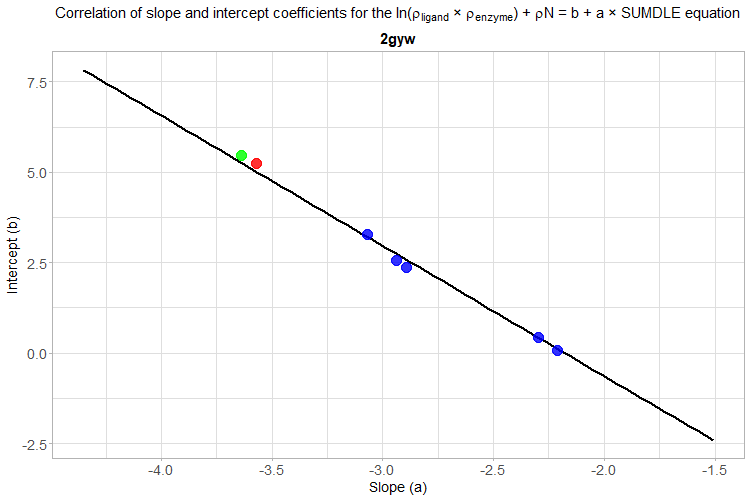

Supplement: Supplementary file 1 — ci0c01382_si_001.zip [file ci0c01382_si_001.zip › Supporting_Information/electron_density_data/ace_chol_esterase/Coefficient data/2gyw.png]

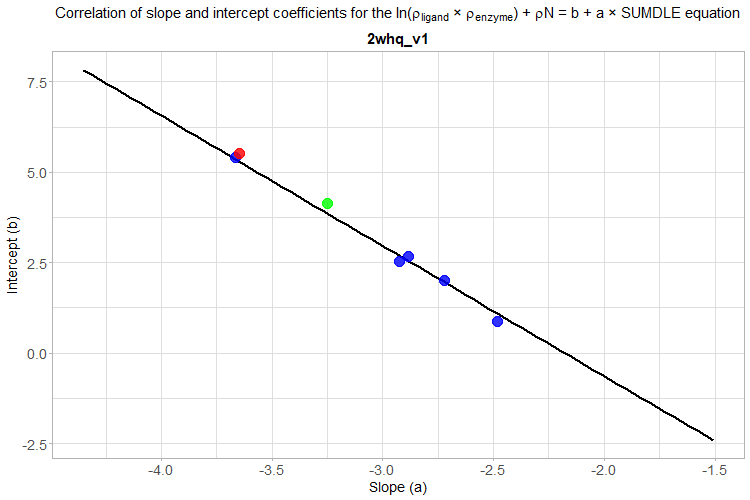

Supplement: Supplementary file 1 — ci0c01382_si_001.zip [file ci0c01382_si_001.zip › Supporting_Information/electron_density_data/ace_chol_esterase/Coefficient data/2whq_v1.png]

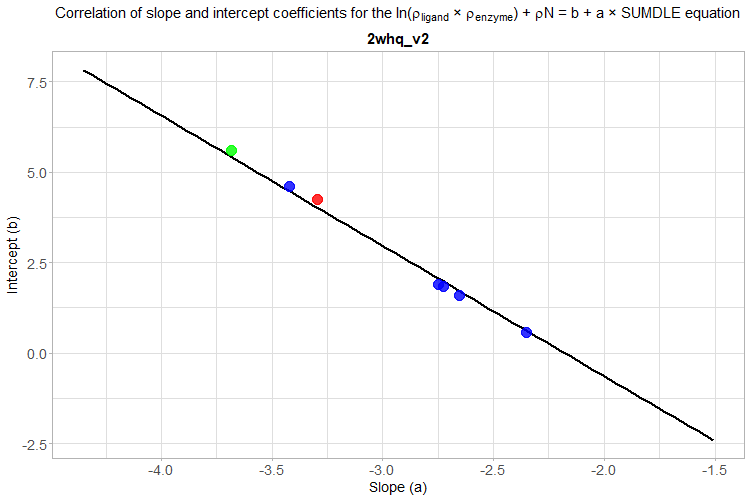

Supplement: Supplementary file 1 — ci0c01382_si_001.zip [file ci0c01382_si_001.zip › Supporting_Information/electron_density_data/ace_chol_esterase/Coefficient data/2whq_v2.png]

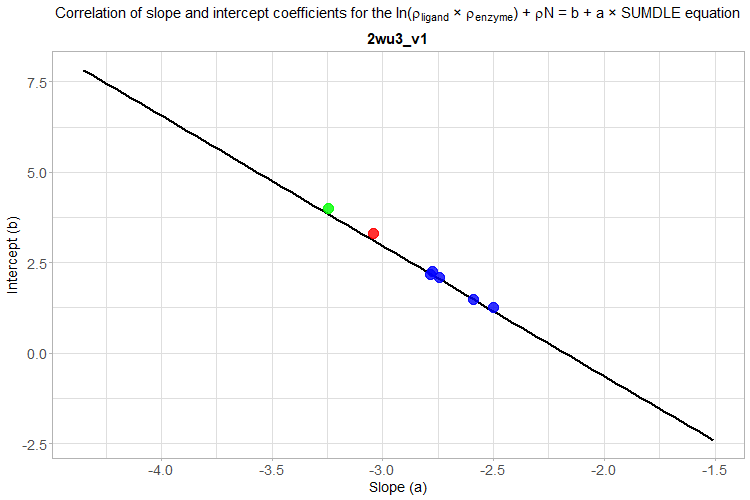

Supplement: Supplementary file 1 — ci0c01382_si_001.zip [file ci0c01382_si_001.zip › Supporting_Information/electron_density_data/ace_chol_esterase/Coefficient data/2wu3_v1.png]

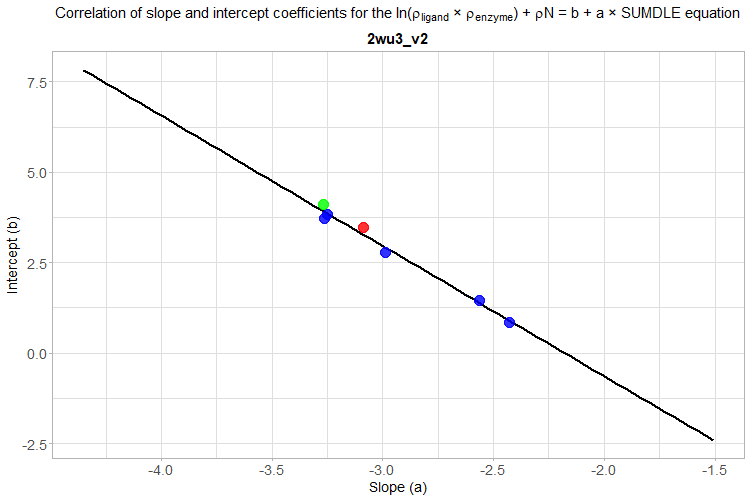

Supplement: Supplementary file 1 — ci0c01382_si_001.zip [file ci0c01382_si_001.zip › Supporting_Information/electron_density_data/ace_chol_esterase/Coefficient data/2wu3_v2.png]

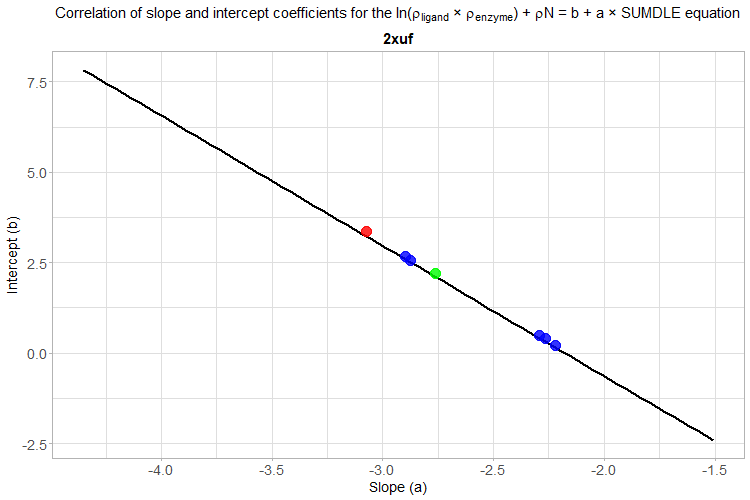

Supplement: Supplementary file 1 — ci0c01382_si_001.zip [file ci0c01382_si_001.zip › Supporting_Information/electron_density_data/ace_chol_esterase/Coefficient data/2xuf.png]

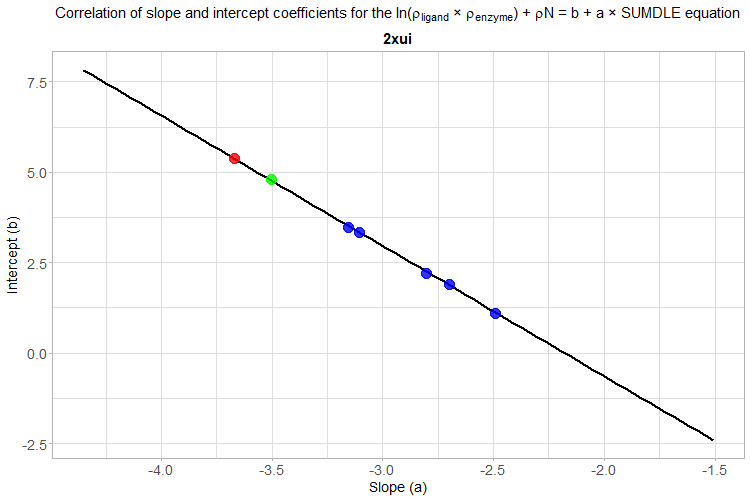

Supplement: Supplementary file 1 — ci0c01382_si_001.zip [file ci0c01382_si_001.zip › Supporting_Information/electron_density_data/ace_chol_esterase/Coefficient data/2xui.png]

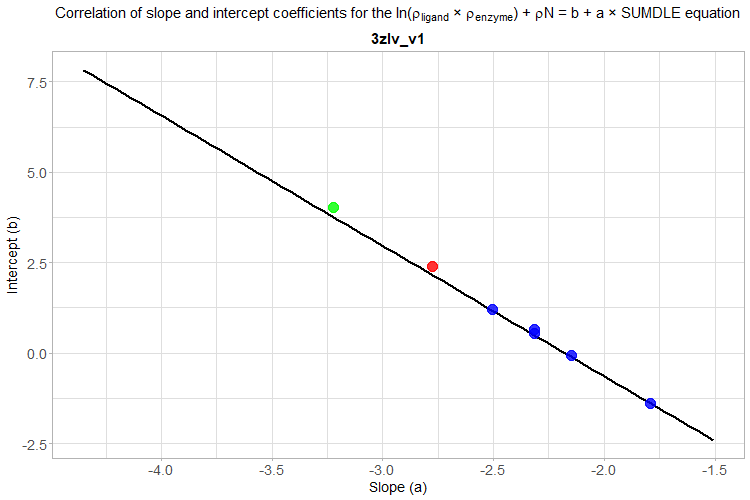

Supplement: Supplementary file 1 — ci0c01382_si_001.zip [file ci0c01382_si_001.zip › Supporting_Information/electron_density_data/ace_chol_esterase/Coefficient data/3zlv_v1.png]

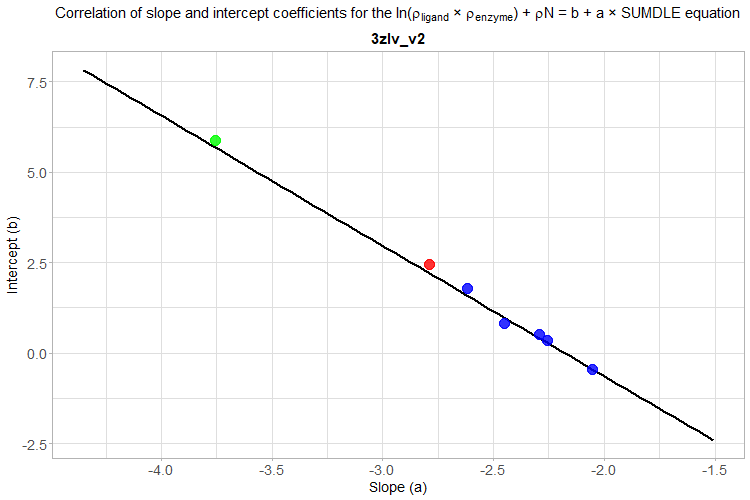

Supplement: Supplementary file 1 — ci0c01382_si_001.zip [file ci0c01382_si_001.zip › Supporting_Information/electron_density_data/ace_chol_esterase/Coefficient data/3zlv_v2.png]

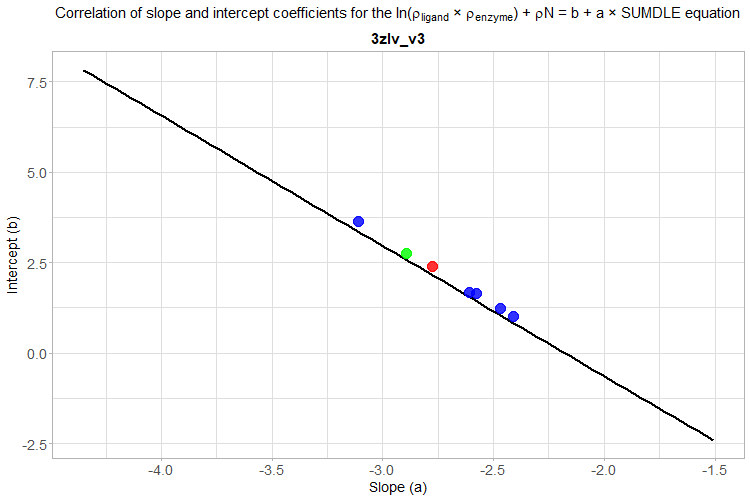

Supplement: Supplementary file 1 — ci0c01382_si_001.zip [file ci0c01382_si_001.zip › Supporting_Information/electron_density_data/ace_chol_esterase/Coefficient data/3zlv_v3.png]

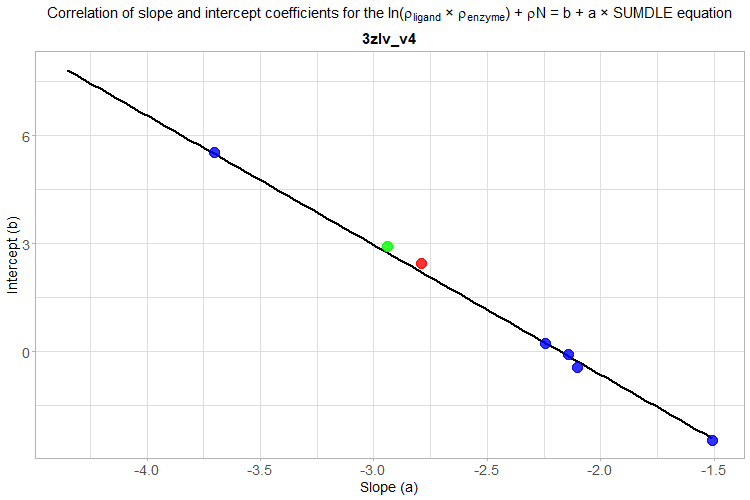

Supplement: Supplementary file 1 — ci0c01382_si_001.zip [file ci0c01382_si_001.zip › Supporting_Information/electron_density_data/ace_chol_esterase/Coefficient data/3zlv_v4.png]

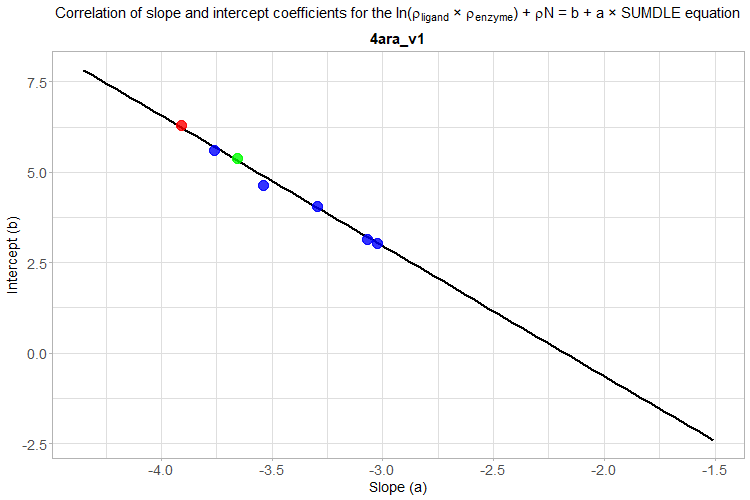

Supplement: Supplementary file 1 — ci0c01382_si_001.zip [file ci0c01382_si_001.zip › Supporting_Information/electron_density_data/ace_chol_esterase/Coefficient data/4ara_v1.png]

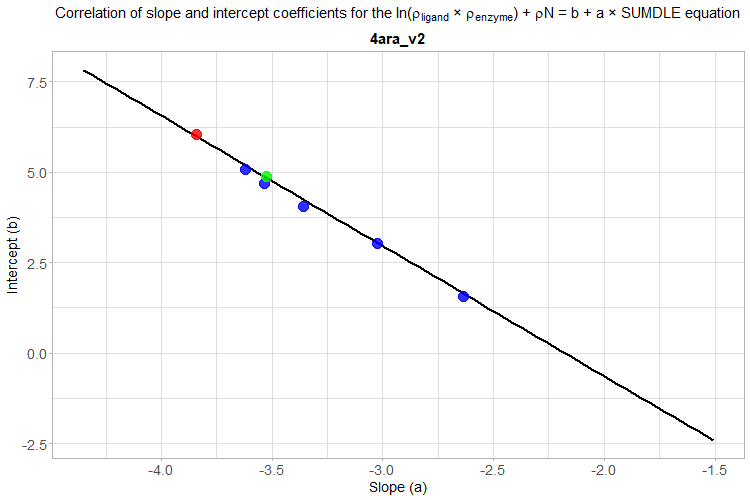

Supplement: Supplementary file 1 — ci0c01382_si_001.zip [file ci0c01382_si_001.zip › Supporting_Information/electron_density_data/ace_chol_esterase/Coefficient data/4ara_v2.png]

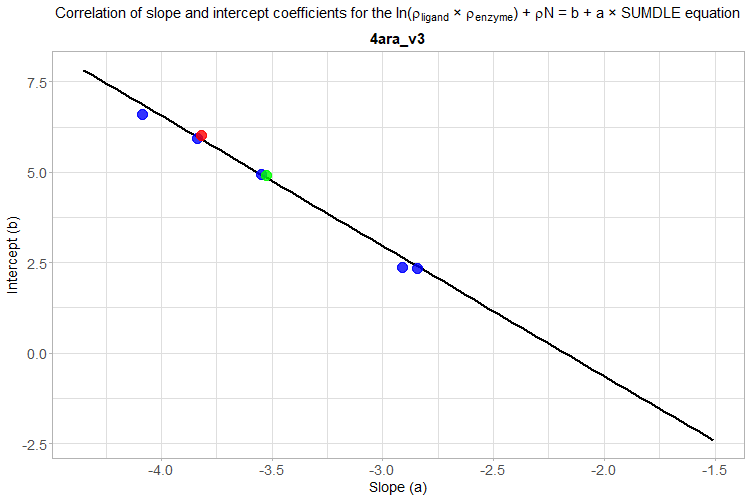

Supplement: Supplementary file 1 — ci0c01382_si_001.zip [file ci0c01382_si_001.zip › Supporting_Information/electron_density_data/ace_chol_esterase/Coefficient data/4ara_v3.png]

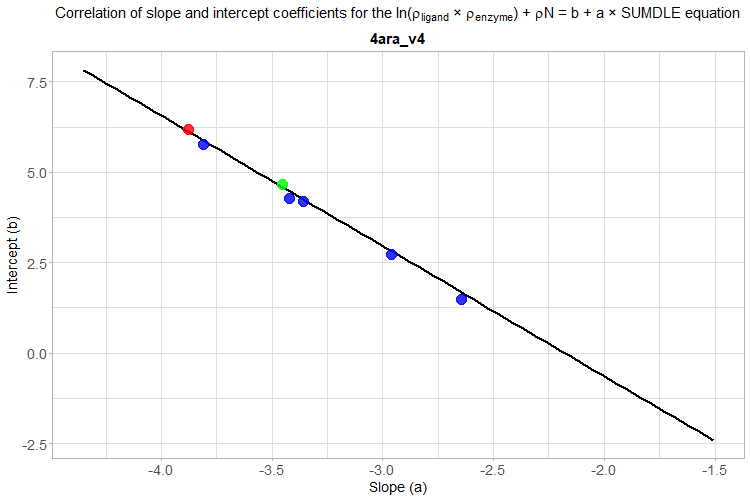

Supplement: Supplementary file 1 — ci0c01382_si_001.zip [file ci0c01382_si_001.zip › Supporting_Information/electron_density_data/ace_chol_esterase/Coefficient data/4ara_v4.png]

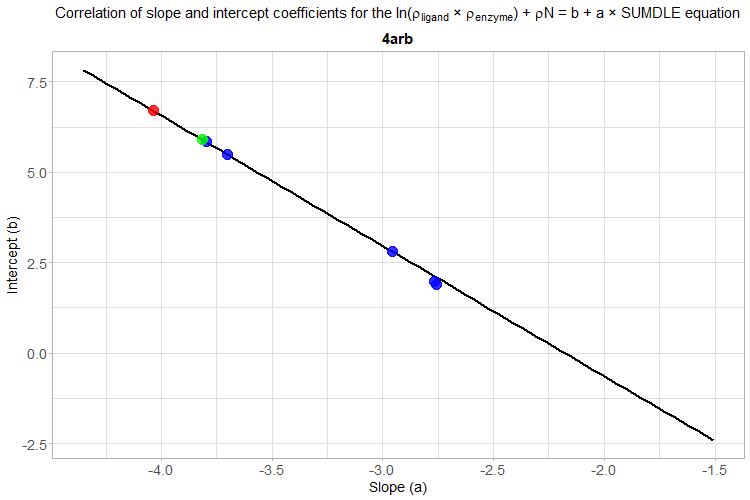

Supplement: Supplementary file 1 — ci0c01382_si_001.zip [file ci0c01382_si_001.zip › Supporting_Information/electron_density_data/ace_chol_esterase/Coefficient data/4arb.png]

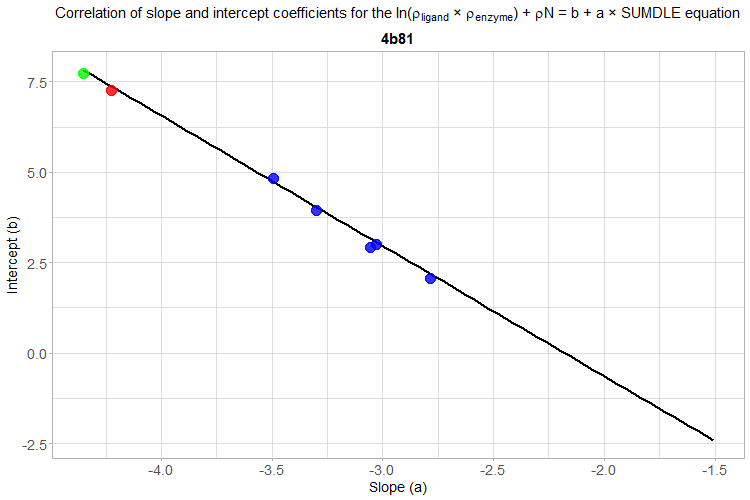

Supplement: Supplementary file 1 — ci0c01382_si_001.zip [file ci0c01382_si_001.zip › Supporting_Information/electron_density_data/ace_chol_esterase/Coefficient data/4b81.png]

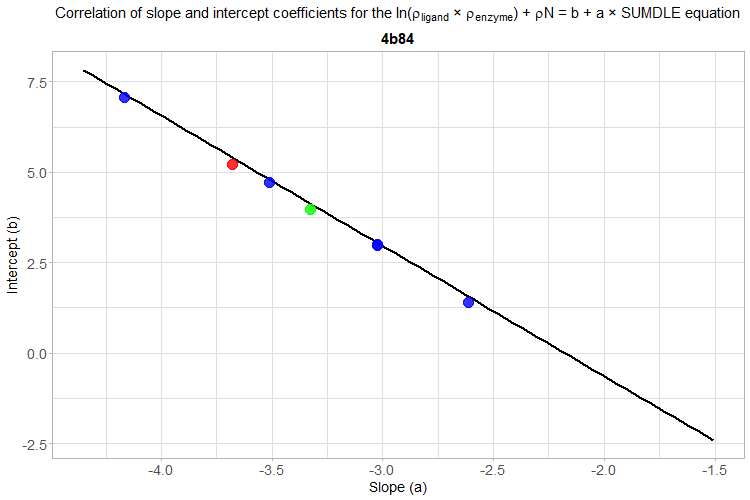

Supplement: Supplementary file 1 — ci0c01382_si_001.zip [file ci0c01382_si_001.zip › Supporting_Information/electron_density_data/ace_chol_esterase/Coefficient data/4b84.png]

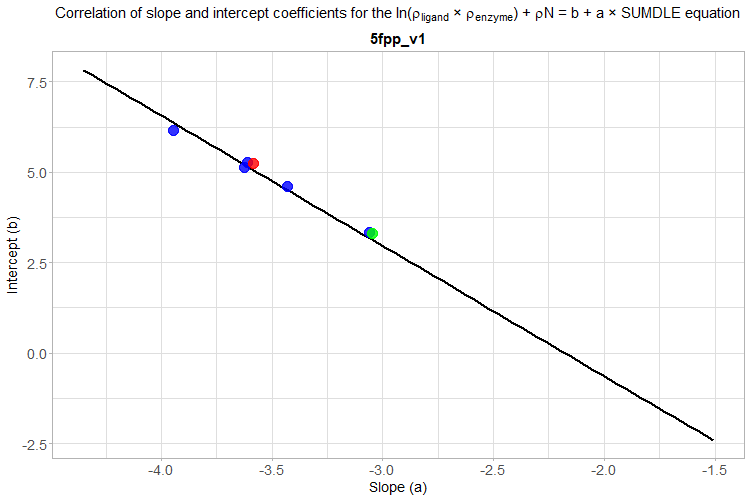

Supplement: Supplementary file 1 — ci0c01382_si_001.zip [file ci0c01382_si_001.zip › Supporting_Information/electron_density_data/ace_chol_esterase/Coefficient data/5fpp_v1.png]

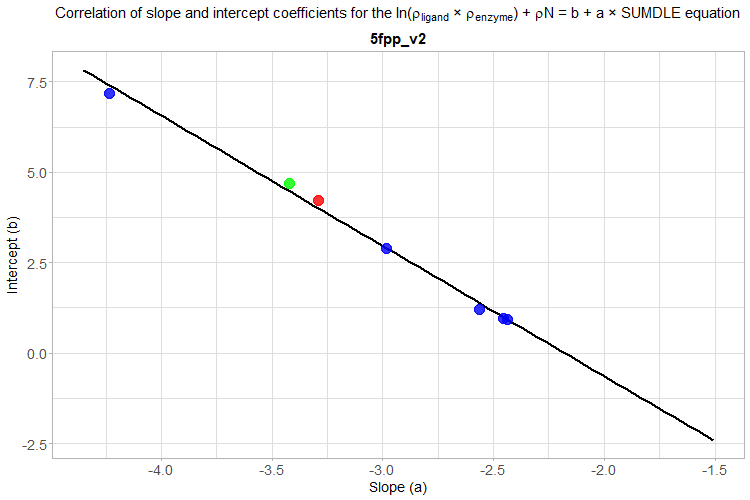

Supplement: Supplementary file 1 — ci0c01382_si_001.zip [file ci0c01382_si_001.zip › Supporting_Information/electron_density_data/ace_chol_esterase/Coefficient data/5fpp_v2.png]

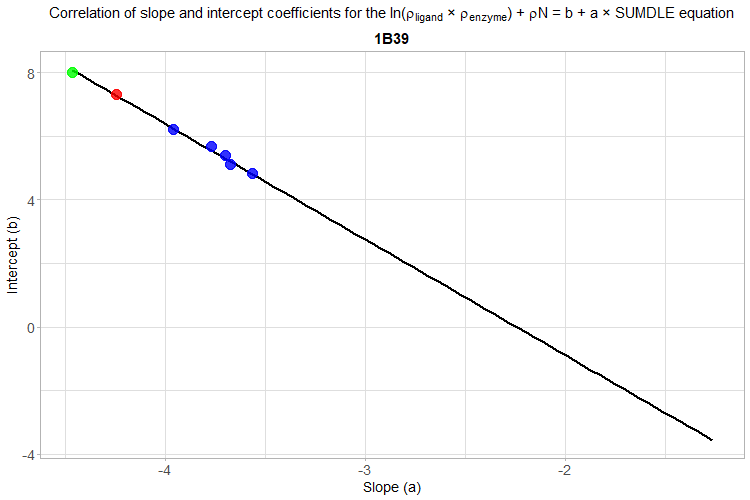

Supplement: Supplementary file 1 — ci0c01382_si_001.zip [file ci0c01382_si_001.zip › Supporting_Information/electron_density_data/CDK2/Coefficient data/1B39.png]

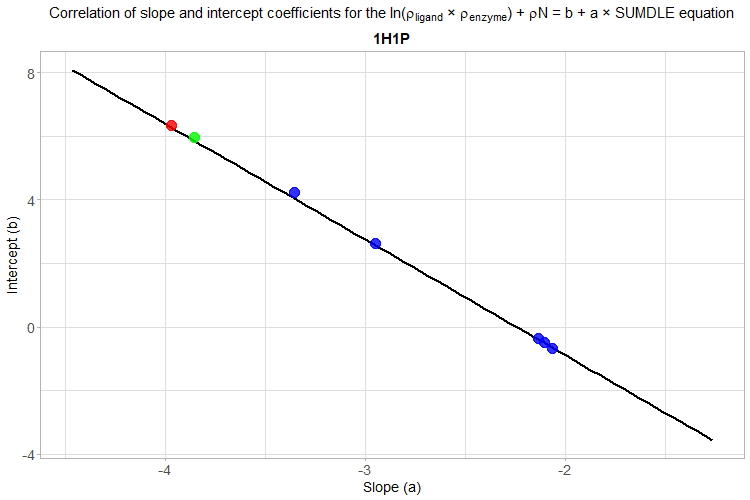

Supplement: Supplementary file 1 — ci0c01382_si_001.zip [file ci0c01382_si_001.zip › Supporting_Information/electron_density_data/CDK2/Coefficient data/1H1P.png]

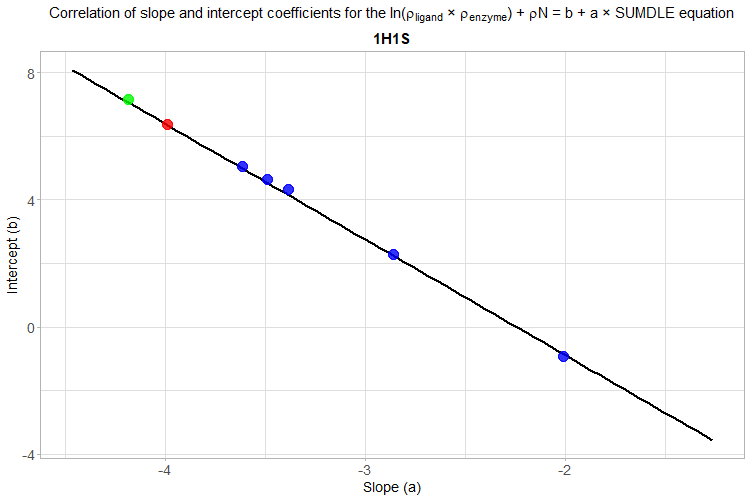

Supplement: Supplementary file 1 — ci0c01382_si_001.zip [file ci0c01382_si_001.zip › Supporting_Information/electron_density_data/CDK2/Coefficient data/1H1S.png]

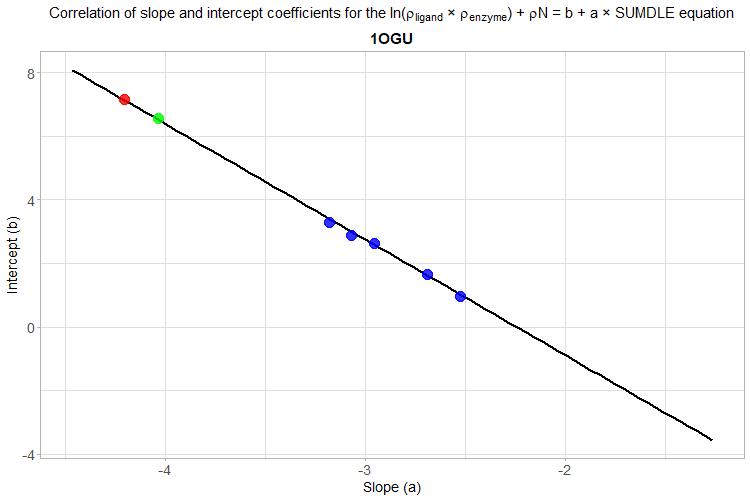

Supplement: Supplementary file 1 — ci0c01382_si_001.zip [file ci0c01382_si_001.zip › Supporting_Information/electron_density_data/CDK2/Coefficient data/1OGU.png]

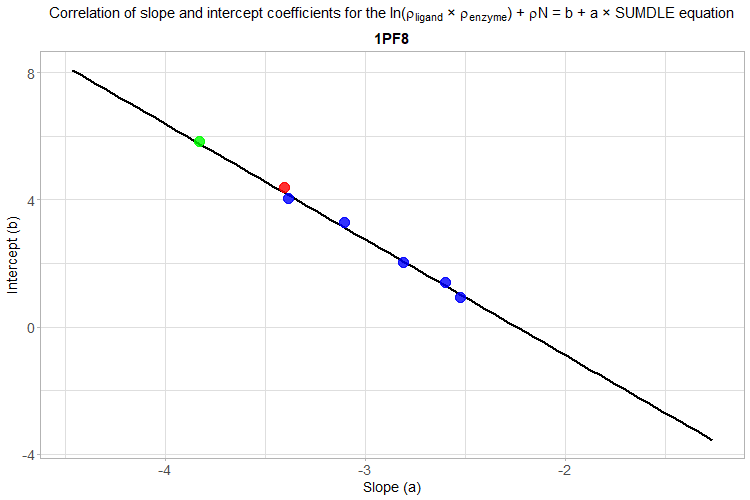

Supplement: Supplementary file 1 — ci0c01382_si_001.zip [file ci0c01382_si_001.zip › Supporting_Information/electron_density_data/CDK2/Coefficient data/1PF8.png]

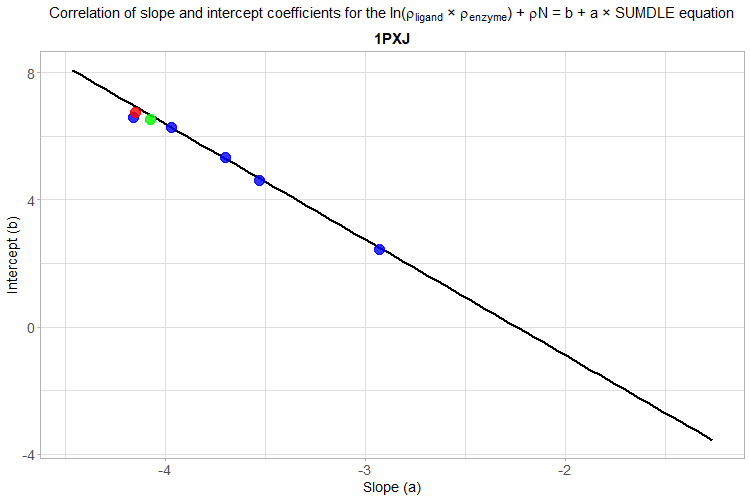

Supplement: Supplementary file 1 — ci0c01382_si_001.zip [file ci0c01382_si_001.zip › Supporting_Information/electron_density_data/CDK2/Coefficient data/1PXJ.png]

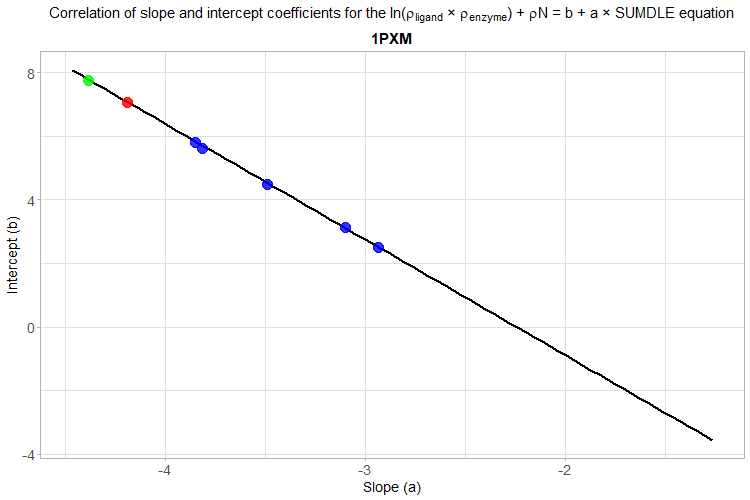

Supplement: Supplementary file 1 — ci0c01382_si_001.zip [file ci0c01382_si_001.zip › Supporting_Information/electron_density_data/CDK2/Coefficient data/1PXM.png]

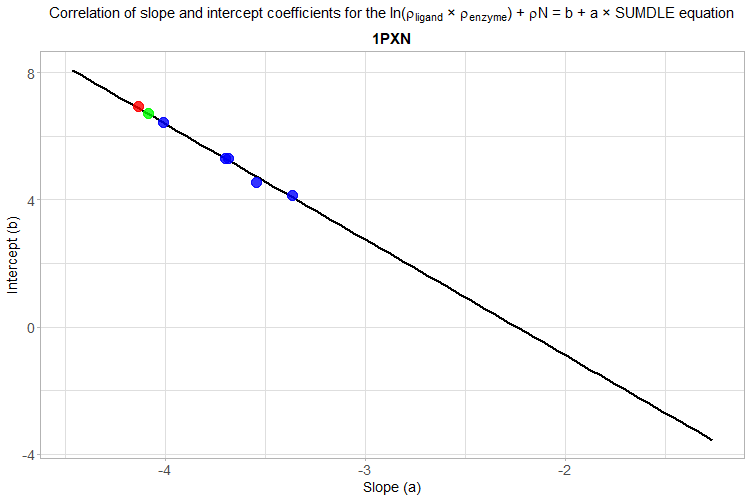

Supplement: Supplementary file 1 — ci0c01382_si_001.zip [file ci0c01382_si_001.zip › Supporting_Information/electron_density_data/CDK2/Coefficient data/1PXN.png]

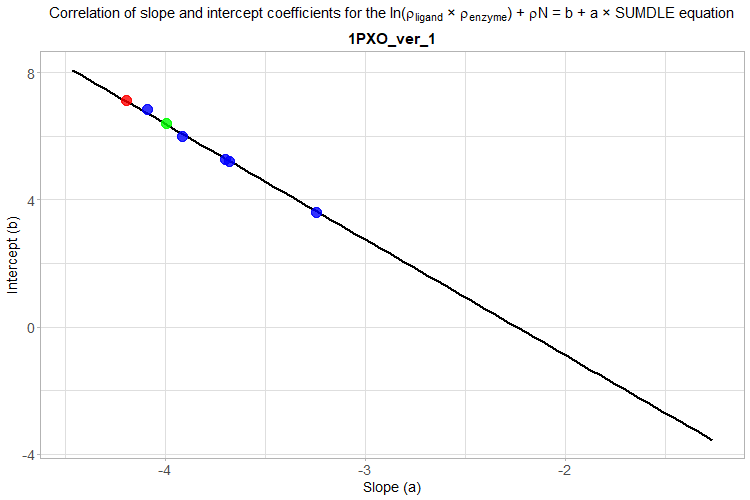

Supplement: Supplementary file 1 — ci0c01382_si_001.zip [file ci0c01382_si_001.zip › Supporting_Information/electron_density_data/CDK2/Coefficient data/1PXO_ver_1.png]

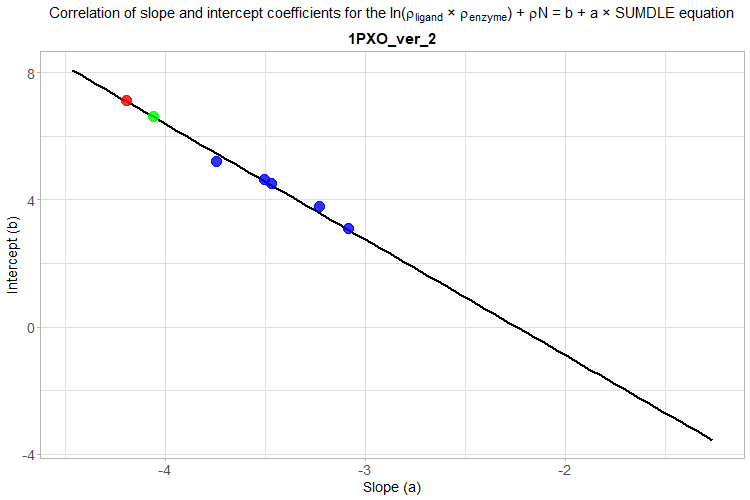

Supplement: Supplementary file 1 — ci0c01382_si_001.zip [file ci0c01382_si_001.zip › Supporting_Information/electron_density_data/CDK2/Coefficient data/1PXO_ver_2.png]

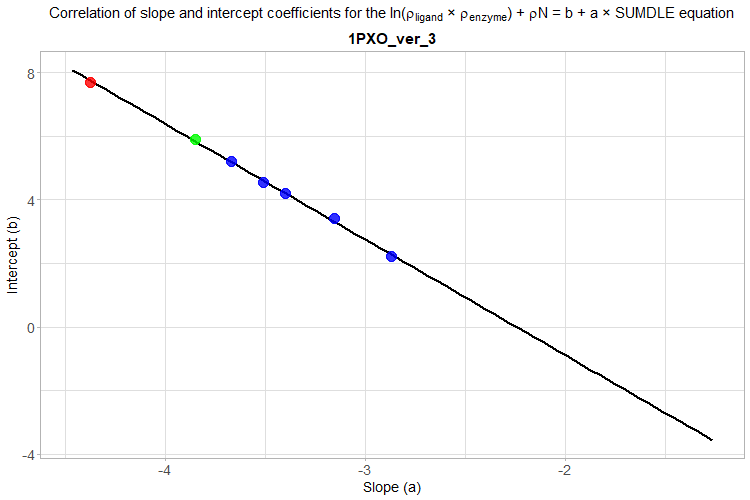

Supplement: Supplementary file 1 — ci0c01382_si_001.zip [file ci0c01382_si_001.zip › Supporting_Information/electron_density_data/CDK2/Coefficient data/1PXO_ver_3.png]

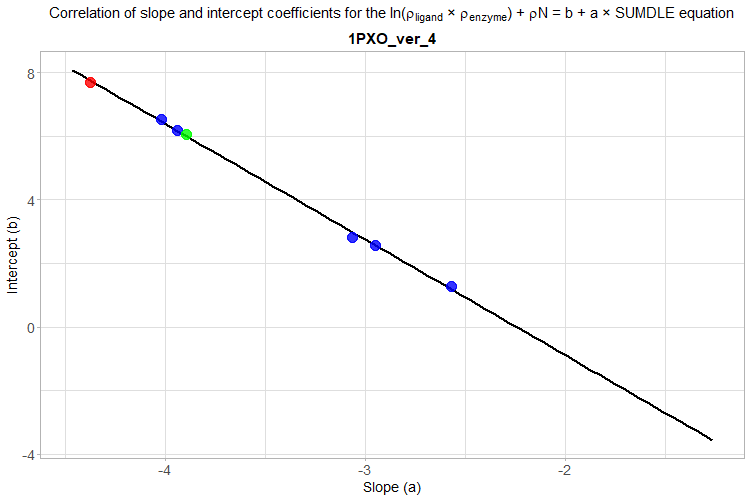

Supplement: Supplementary file 1 — ci0c01382_si_001.zip [file ci0c01382_si_001.zip › Supporting_Information/electron_density_data/CDK2/Coefficient data/1PXO_ver_4.png]

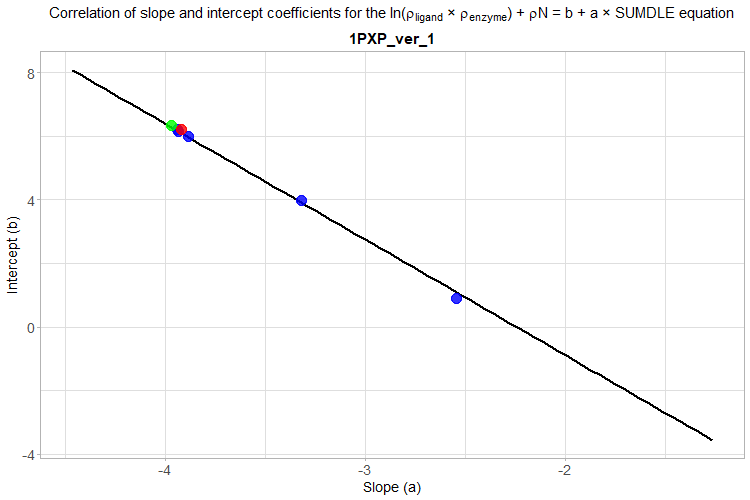

Supplement: Supplementary file 1 — ci0c01382_si_001.zip [file ci0c01382_si_001.zip › Supporting_Information/electron_density_data/CDK2/Coefficient data/1PXP_ver_1.png]

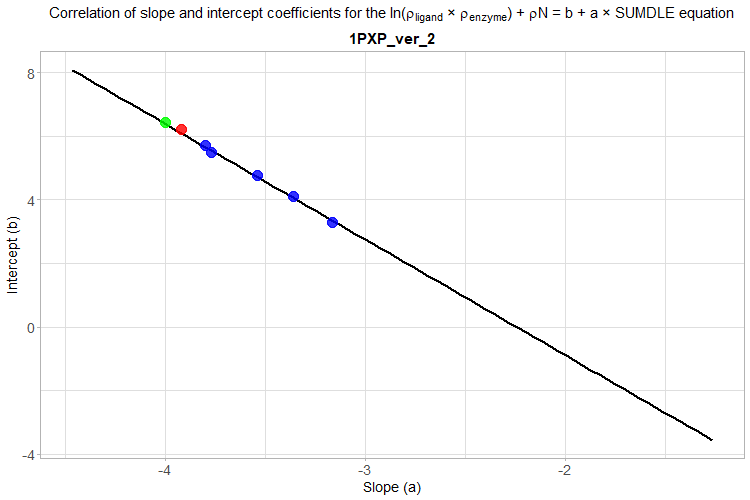

Supplement: Supplementary file 1 — ci0c01382_si_001.zip [file ci0c01382_si_001.zip › Supporting_Information/electron_density_data/CDK2/Coefficient data/1PXP_ver_2.png]

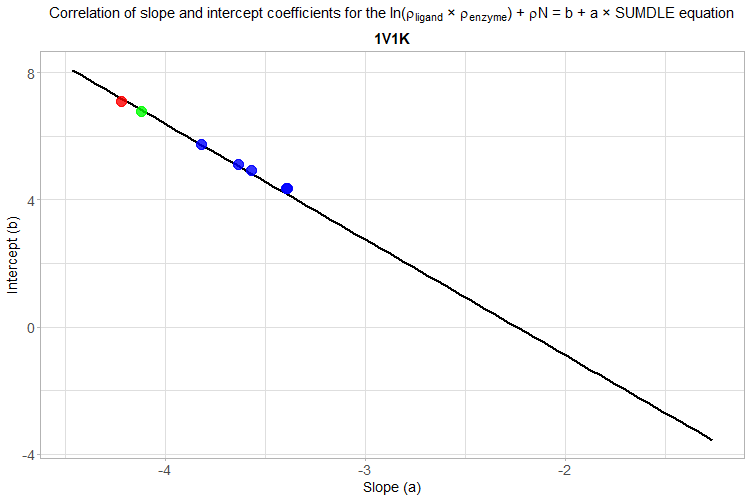

Supplement: Supplementary file 1 — ci0c01382_si_001.zip [file ci0c01382_si_001.zip › Supporting_Information/electron_density_data/CDK2/Coefficient data/1V1K.png]

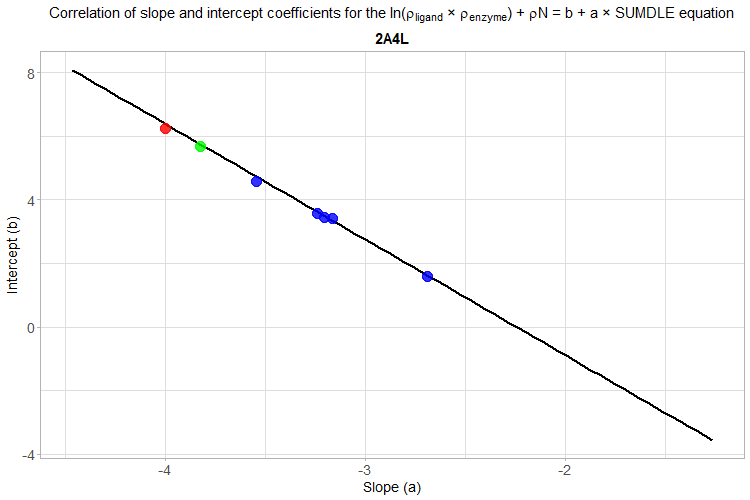

Supplement: Supplementary file 1 — ci0c01382_si_001.zip [file ci0c01382_si_001.zip › Supporting_Information/electron_density_data/CDK2/Coefficient data/2A4L.png]

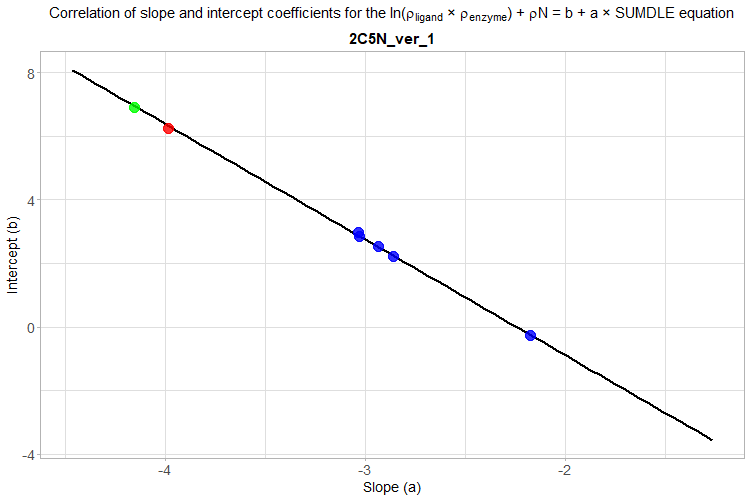

Supplement: Supplementary file 1 — ci0c01382_si_001.zip [file ci0c01382_si_001.zip › Supporting_Information/electron_density_data/CDK2/Coefficient data/2C5N_ver_1.png]

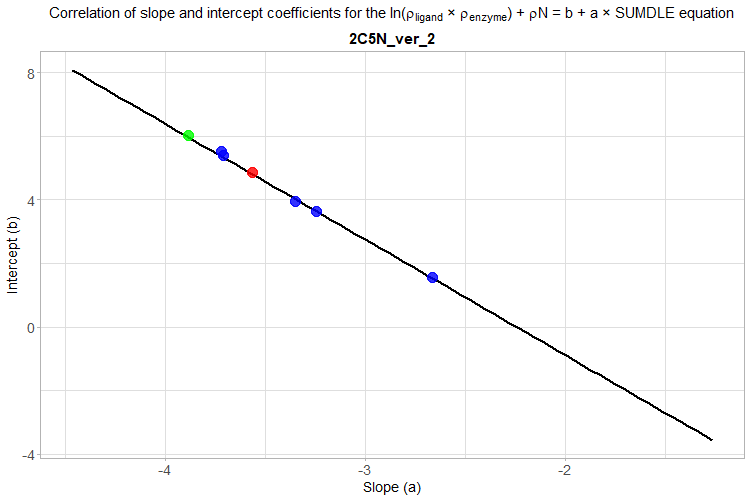

Supplement: Supplementary file 1 — ci0c01382_si_001.zip [file ci0c01382_si_001.zip › Supporting_Information/electron_density_data/CDK2/Coefficient data/2C5N_ver_2.png]

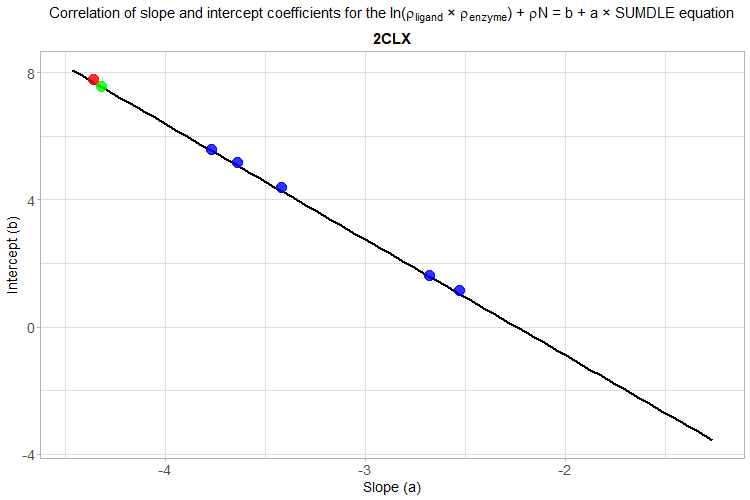

Supplement: Supplementary file 1 — ci0c01382_si_001.zip [file ci0c01382_si_001.zip › Supporting_Information/electron_density_data/CDK2/Coefficient data/2CLX.png]

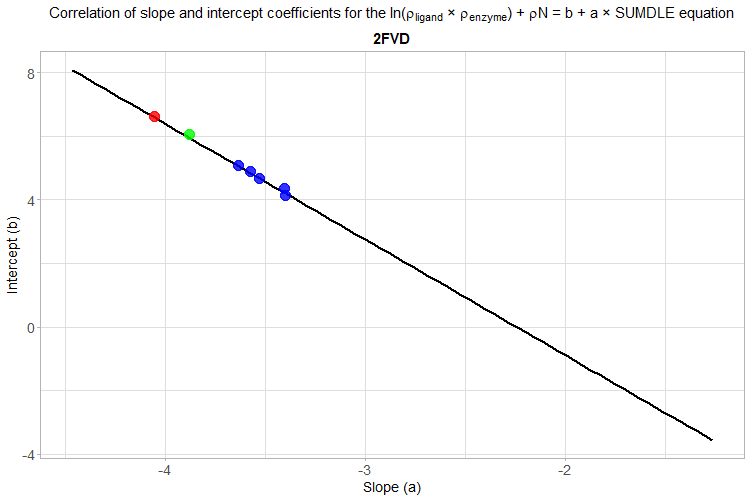

Supplement: Supplementary file 1 — ci0c01382_si_001.zip [file ci0c01382_si_001.zip › Supporting_Information/electron_density_data/CDK2/Coefficient data/2FVD.png]

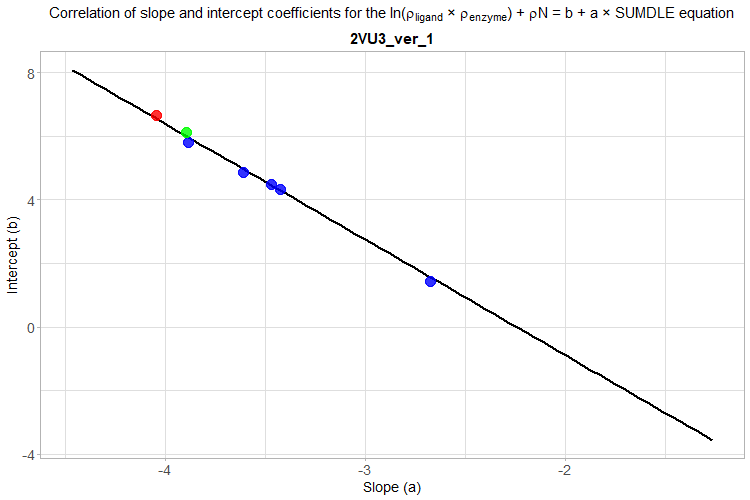

Supplement: Supplementary file 1 — ci0c01382_si_001.zip [file ci0c01382_si_001.zip › Supporting_Information/electron_density_data/CDK2/Coefficient data/2VU3_ver_1.png]

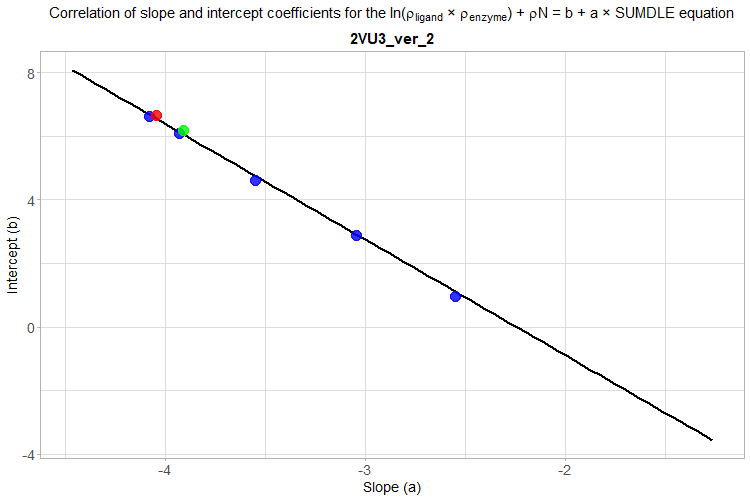

Supplement: Supplementary file 1 — ci0c01382_si_001.zip [file ci0c01382_si_001.zip › Supporting_Information/electron_density_data/CDK2/Coefficient data/2VU3_ver_2.png]

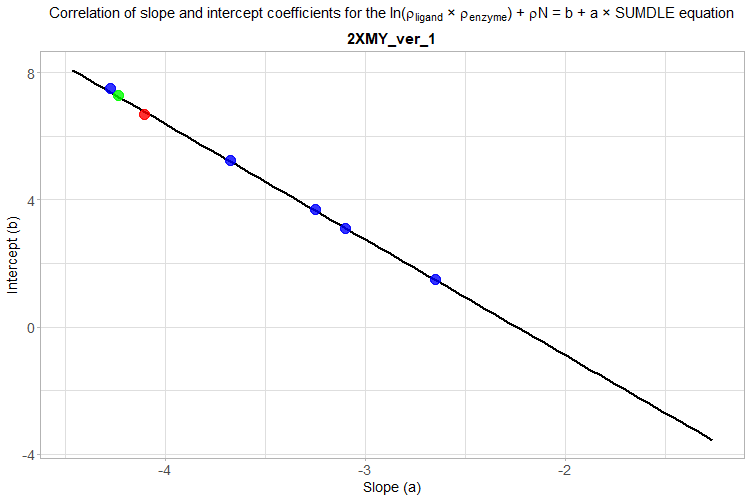

Supplement: Supplementary file 1 — ci0c01382_si_001.zip [file ci0c01382_si_001.zip › Supporting_Information/electron_density_data/CDK2/Coefficient data/2XMY_ver_1.png]

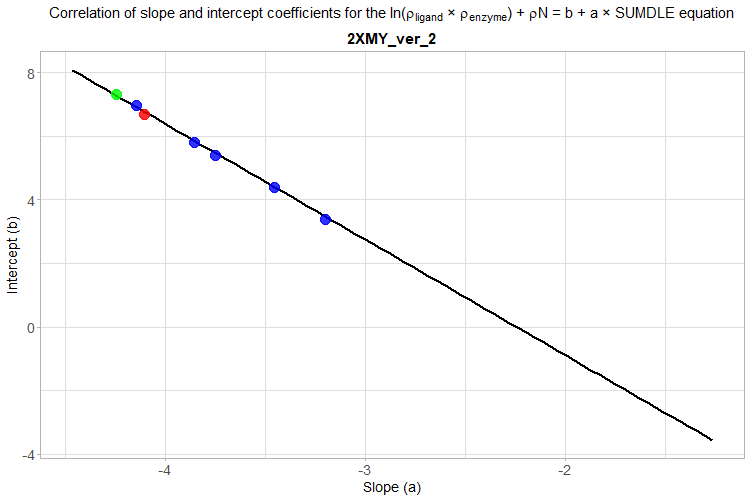

Supplement: Supplementary file 1 — ci0c01382_si_001.zip [file ci0c01382_si_001.zip › Supporting_Information/electron_density_data/CDK2/Coefficient data/2XMY_ver_2.png]

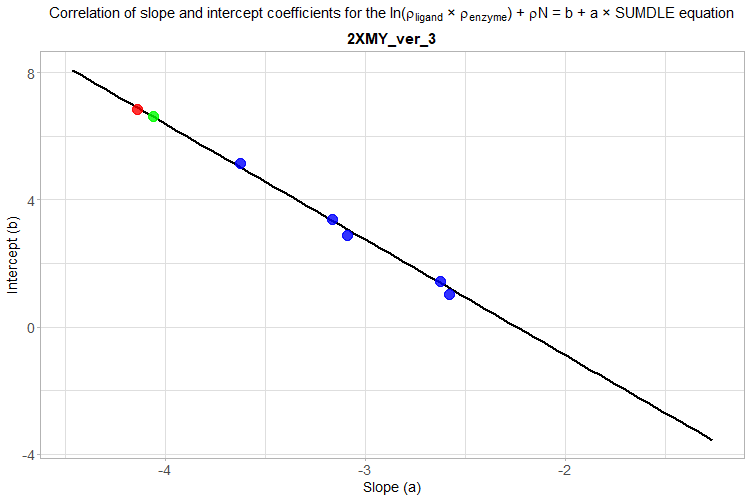

Supplement: Supplementary file 1 — ci0c01382_si_001.zip [file ci0c01382_si_001.zip › Supporting_Information/electron_density_data/CDK2/Coefficient data/2XMY_ver_3.png]

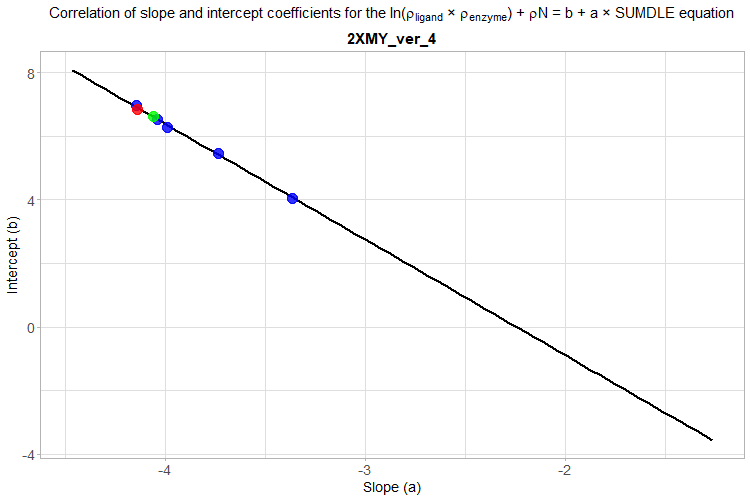

Supplement: Supplementary file 1 — ci0c01382_si_001.zip [file ci0c01382_si_001.zip › Supporting_Information/electron_density_data/CDK2/Coefficient data/2XMY_ver_4.png]

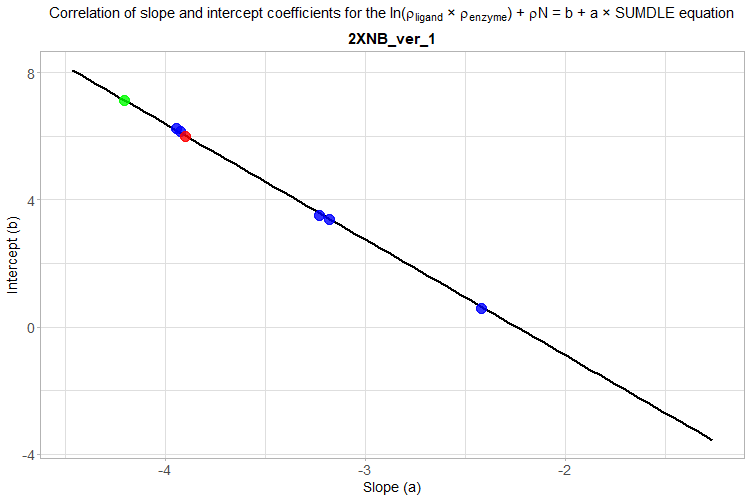

Supplement: Supplementary file 1 — ci0c01382_si_001.zip [file ci0c01382_si_001.zip › Supporting_Information/electron_density_data/CDK2/Coefficient data/2XNB_ver_1.png]

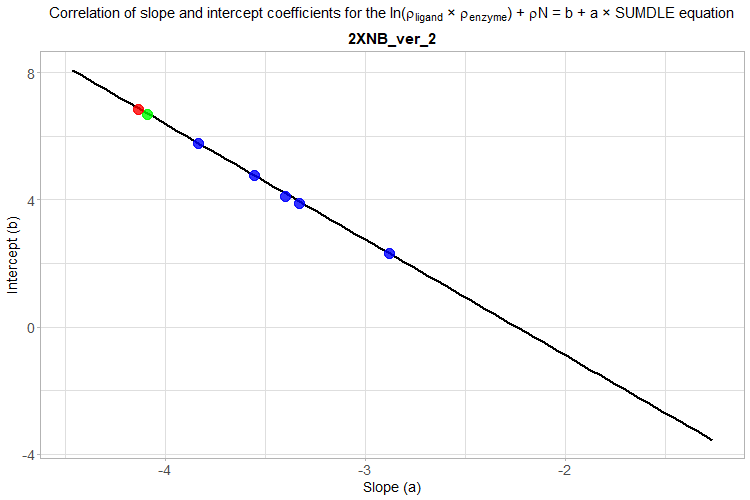

Supplement: Supplementary file 1 — ci0c01382_si_001.zip [file ci0c01382_si_001.zip › Supporting_Information/electron_density_data/CDK2/Coefficient data/2XNB_ver_2.png]

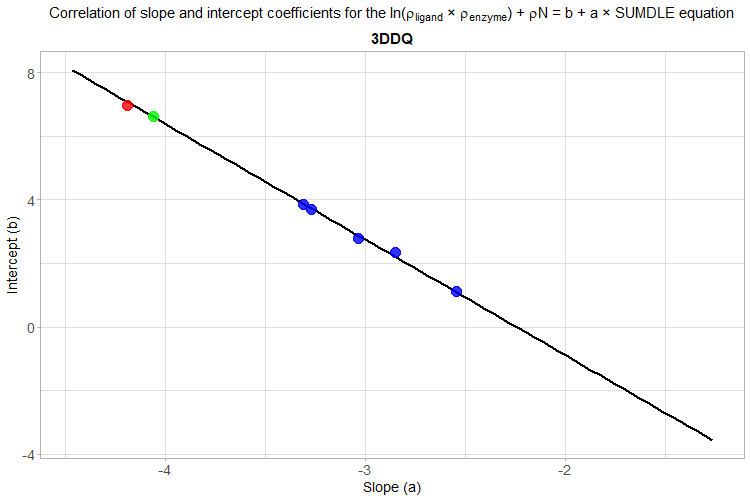

Supplement: Supplementary file 1 — ci0c01382_si_001.zip [file ci0c01382_si_001.zip › Supporting_Information/electron_density_data/CDK2/Coefficient data/3DDQ.png]

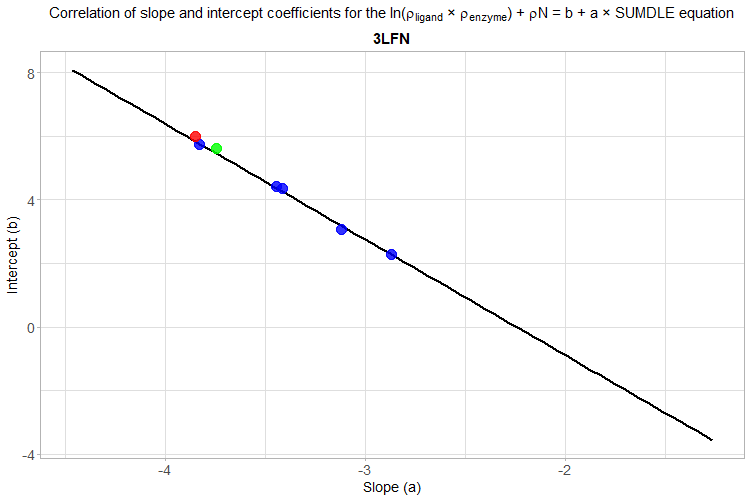

Supplement: Supplementary file 1 — ci0c01382_si_001.zip [file ci0c01382_si_001.zip › Supporting_Information/electron_density_data/CDK2/Coefficient data/3LFN.png]

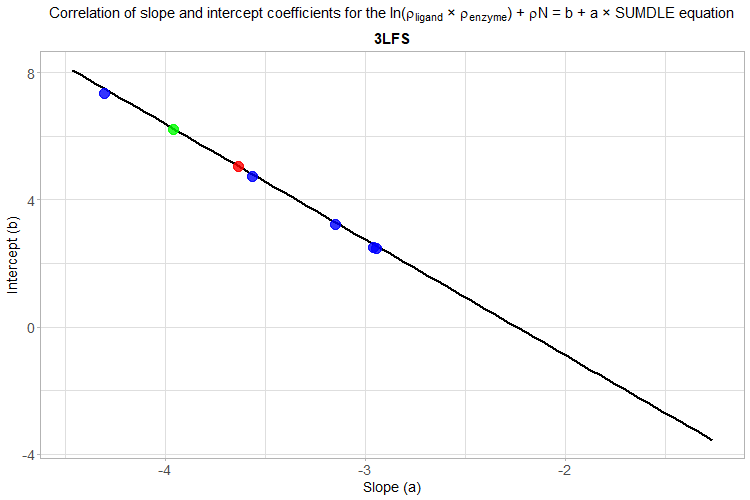

Supplement: Supplementary file 1 — ci0c01382_si_001.zip [file ci0c01382_si_001.zip › Supporting_Information/electron_density_data/CDK2/Coefficient data/3LFS.png]

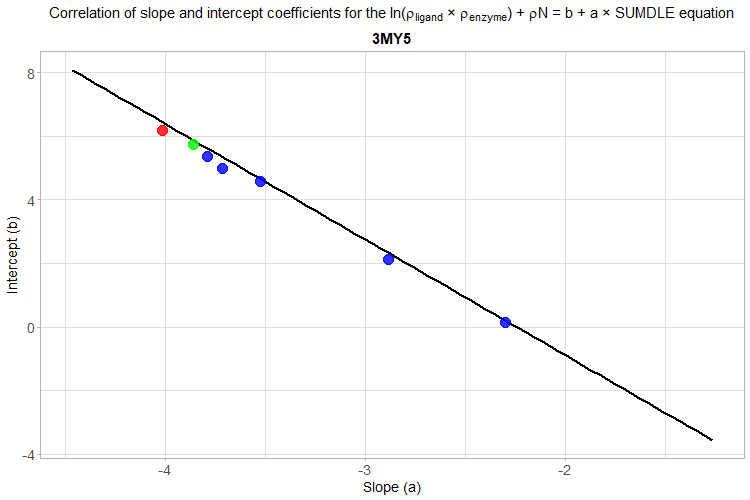

Supplement: Supplementary file 1 — ci0c01382_si_001.zip [file ci0c01382_si_001.zip › Supporting_Information/electron_density_data/CDK2/Coefficient data/3MY5.png]

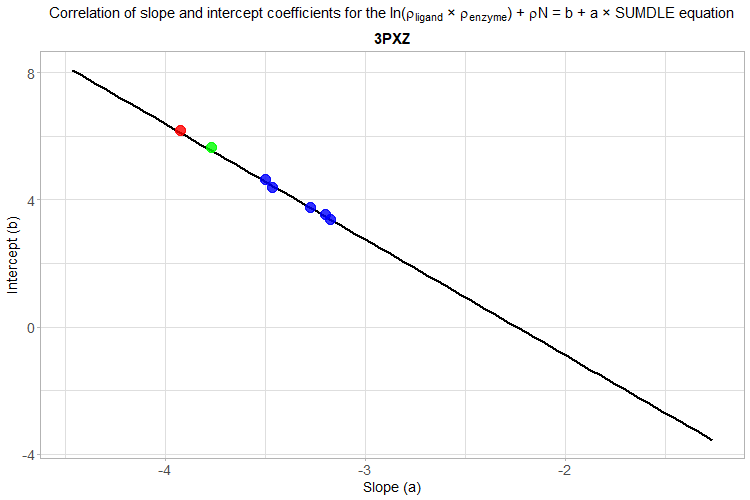

Supplement: Supplementary file 1 — ci0c01382_si_001.zip [file ci0c01382_si_001.zip › Supporting_Information/electron_density_data/CDK2/Coefficient data/3PXZ.png]

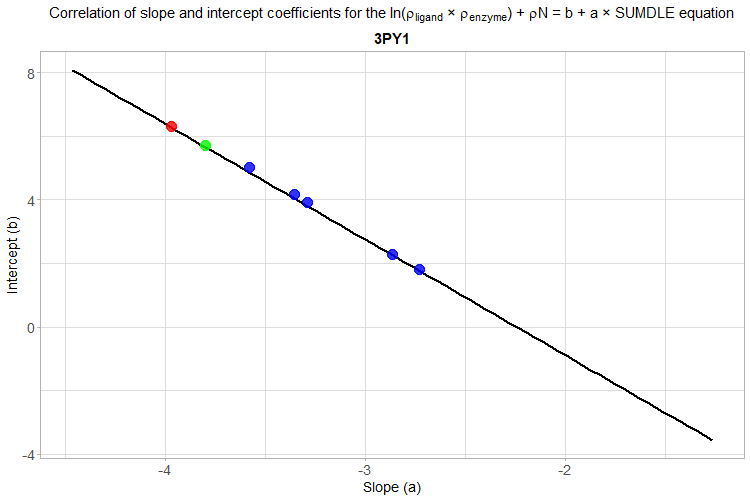

Supplement: Supplementary file 1 — ci0c01382_si_001.zip [file ci0c01382_si_001.zip › Supporting_Information/electron_density_data/CDK2/Coefficient data/3PY1.png]

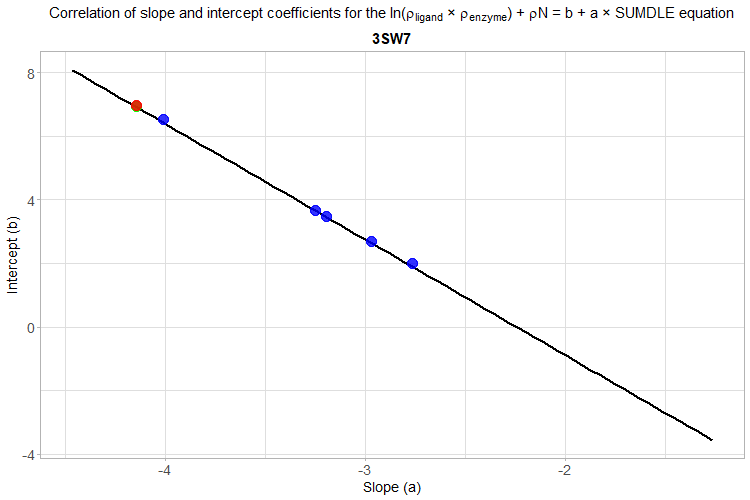

Supplement: Supplementary file 1 — ci0c01382_si_001.zip [file ci0c01382_si_001.zip › Supporting_Information/electron_density_data/CDK2/Coefficient data/3SW7.png]

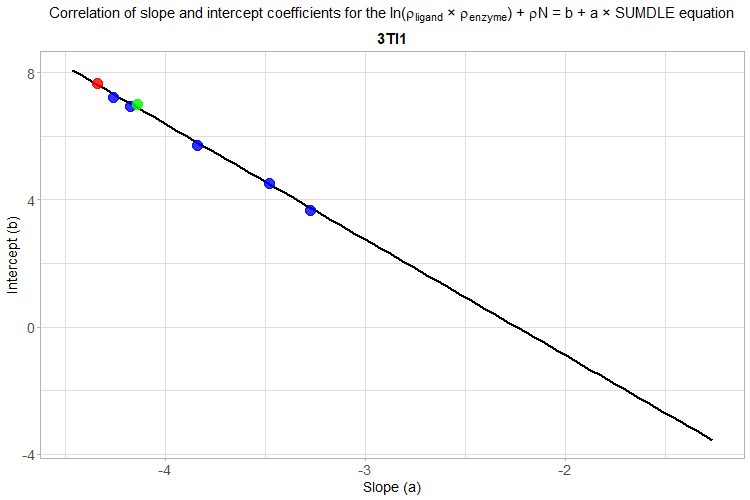

Supplement: Supplementary file 1 — ci0c01382_si_001.zip [file ci0c01382_si_001.zip › Supporting_Information/electron_density_data/CDK2/Coefficient data/3TI1.png]

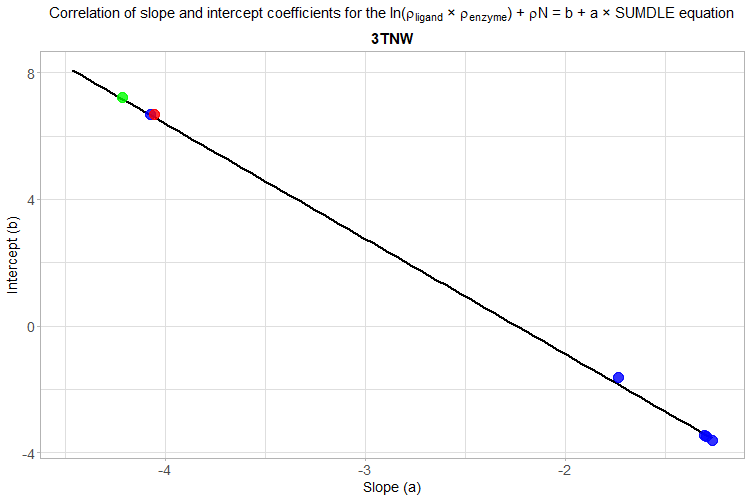

Supplement: Supplementary file 1 — ci0c01382_si_001.zip [file ci0c01382_si_001.zip › Supporting_Information/electron_density_data/CDK2/Coefficient data/3TNW.png]

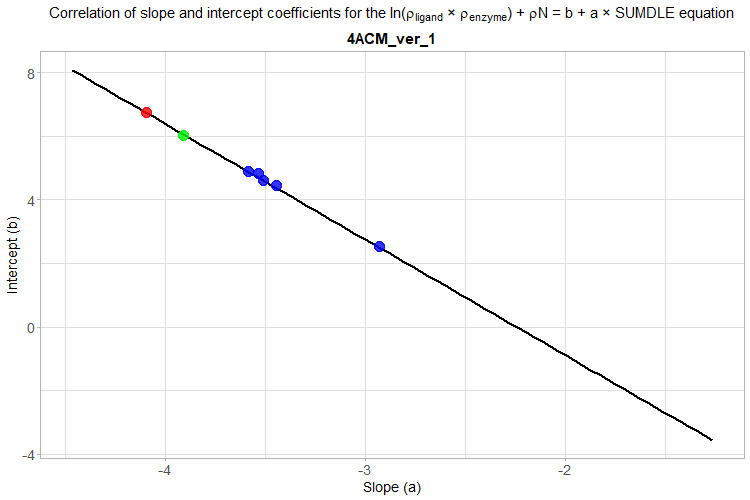

Supplement: Supplementary file 1 — ci0c01382_si_001.zip [file ci0c01382_si_001.zip › Supporting_Information/electron_density_data/CDK2/Coefficient data/4ACM_ver_1.png]

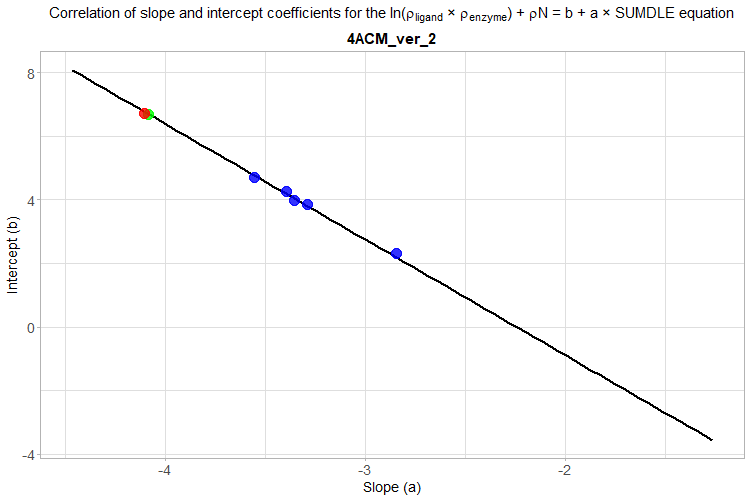

Supplement: Supplementary file 1 — ci0c01382_si_001.zip [file ci0c01382_si_001.zip › Supporting_Information/electron_density_data/CDK2/Coefficient data/4ACM_ver_2.png]

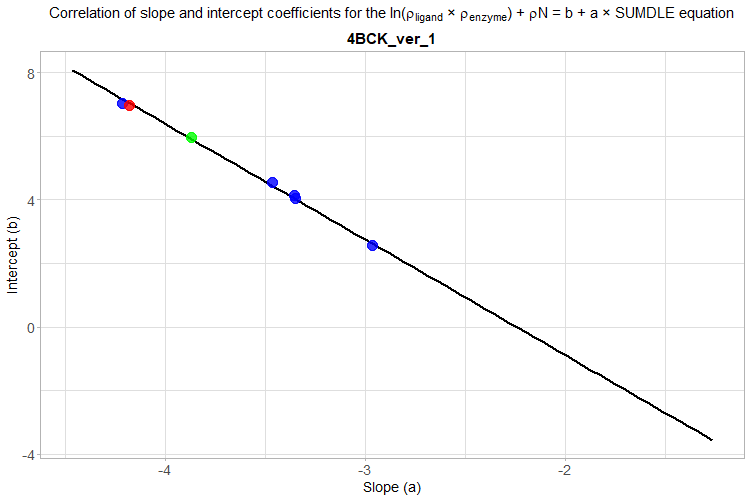

Supplement: Supplementary file 1 — ci0c01382_si_001.zip [file ci0c01382_si_001.zip › Supporting_Information/electron_density_data/CDK2/Coefficient data/4BCK_ver_1.png]

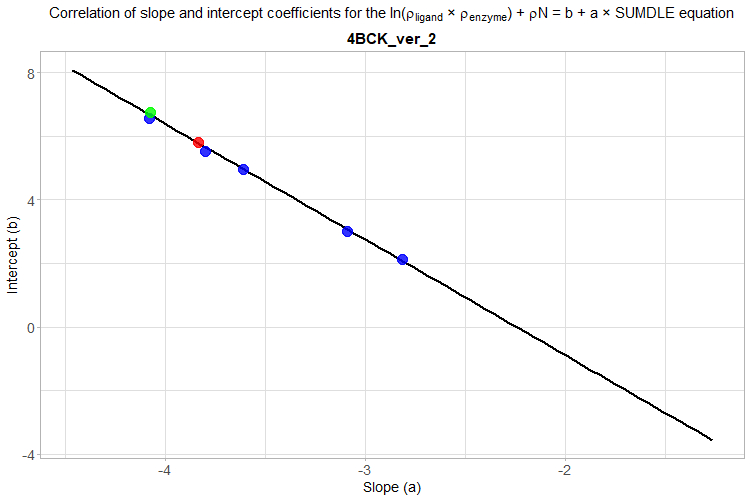

Supplement: Supplementary file 1 — ci0c01382_si_001.zip [file ci0c01382_si_001.zip › Supporting_Information/electron_density_data/CDK2/Coefficient data/4BCK_ver_2.png]

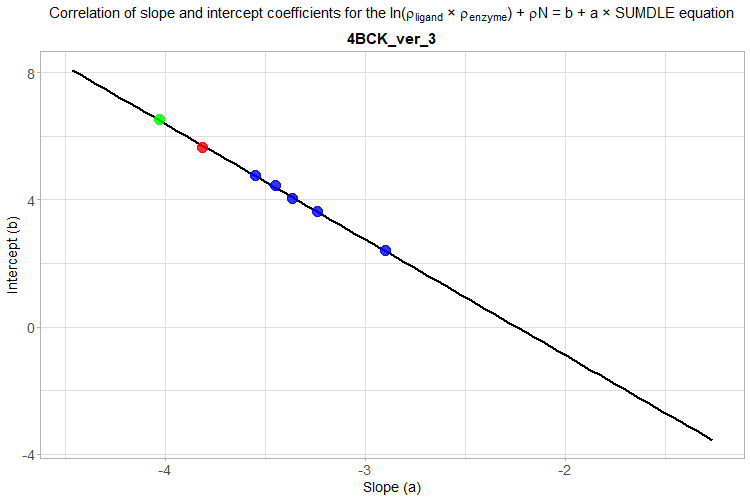

Supplement: Supplementary file 1 — ci0c01382_si_001.zip [file ci0c01382_si_001.zip › Supporting_Information/electron_density_data/CDK2/Coefficient data/4BCK_ver_3.png]

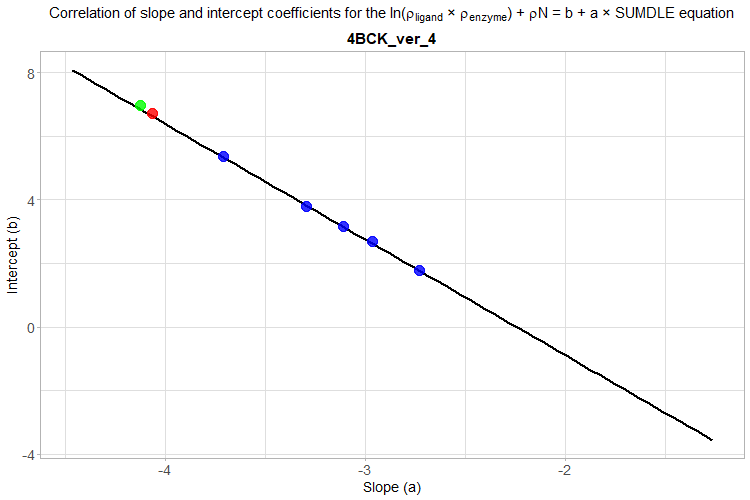

Supplement: Supplementary file 1 — ci0c01382_si_001.zip [file ci0c01382_si_001.zip › Supporting_Information/electron_density_data/CDK2/Coefficient data/4BCK_ver_4.png]

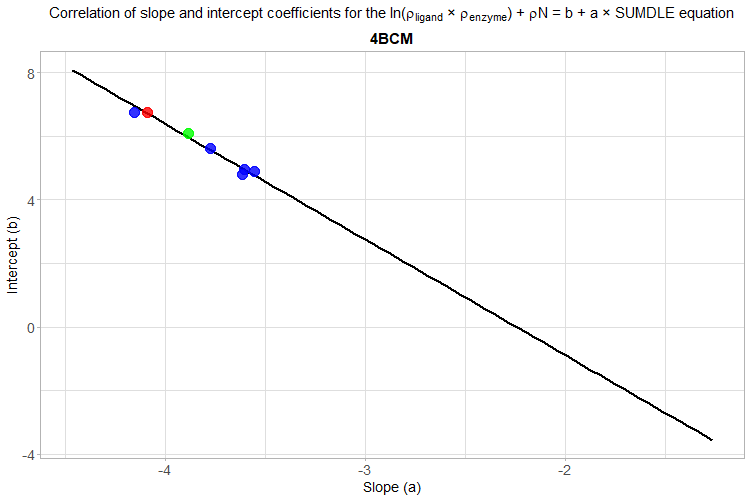

Supplement: Supplementary file 1 — ci0c01382_si_001.zip [file ci0c01382_si_001.zip › Supporting_Information/electron_density_data/CDK2/Coefficient data/4BCM.png]

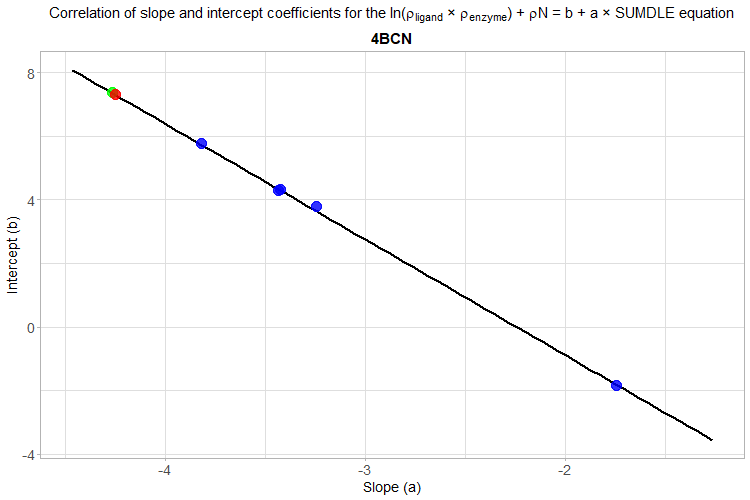

Supplement: Supplementary file 1 — ci0c01382_si_001.zip [file ci0c01382_si_001.zip › Supporting_Information/electron_density_data/CDK2/Coefficient data/4BCN.png]

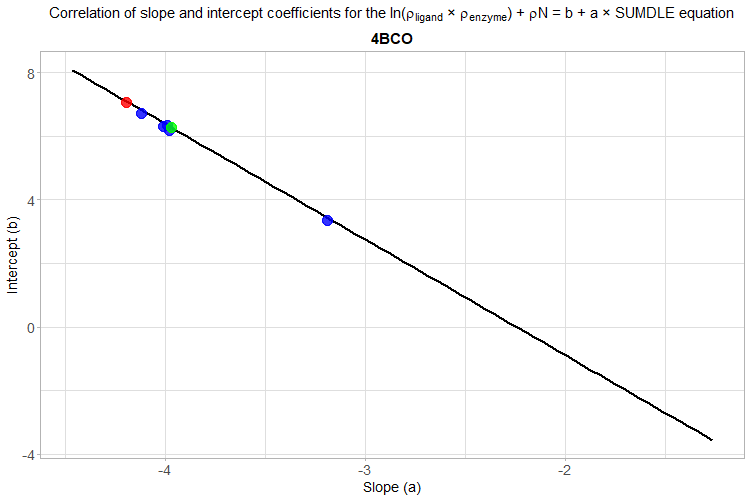

Supplement: Supplementary file 1 — ci0c01382_si_001.zip [file ci0c01382_si_001.zip › Supporting_Information/electron_density_data/CDK2/Coefficient data/4BCO.png]

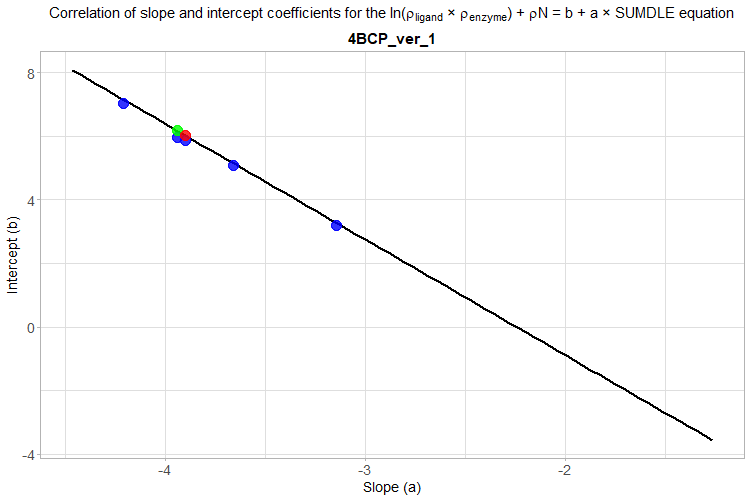

Supplement: Supplementary file 1 — ci0c01382_si_001.zip [file ci0c01382_si_001.zip › Supporting_Information/electron_density_data/CDK2/Coefficient data/4BCP_ver_1.png]

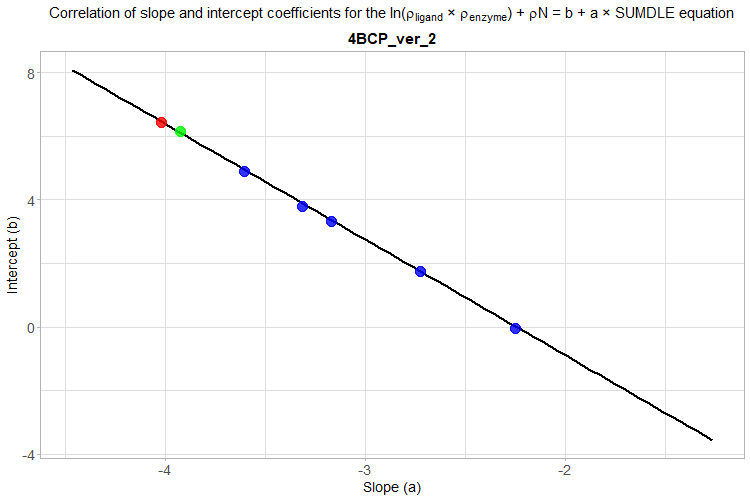

Supplement: Supplementary file 1 — ci0c01382_si_001.zip [file ci0c01382_si_001.zip › Supporting_Information/electron_density_data/CDK2/Coefficient data/4BCP_ver_2.png]

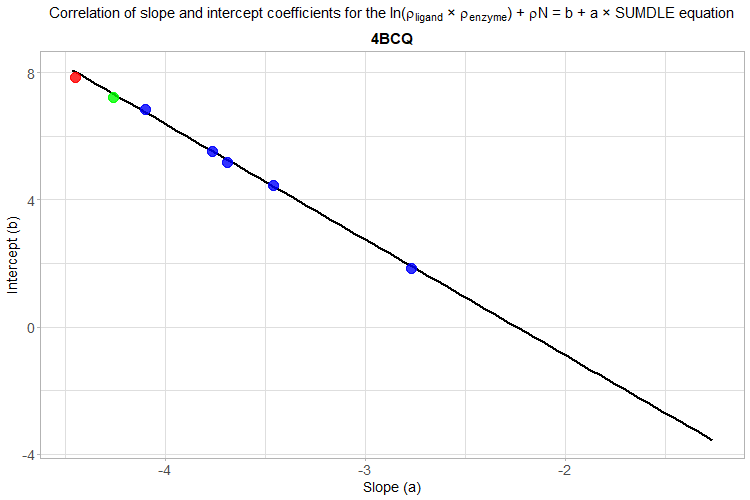

Supplement: Supplementary file 1 — ci0c01382_si_001.zip [file ci0c01382_si_001.zip › Supporting_Information/electron_density_data/CDK2/Coefficient data/4BCQ.png]

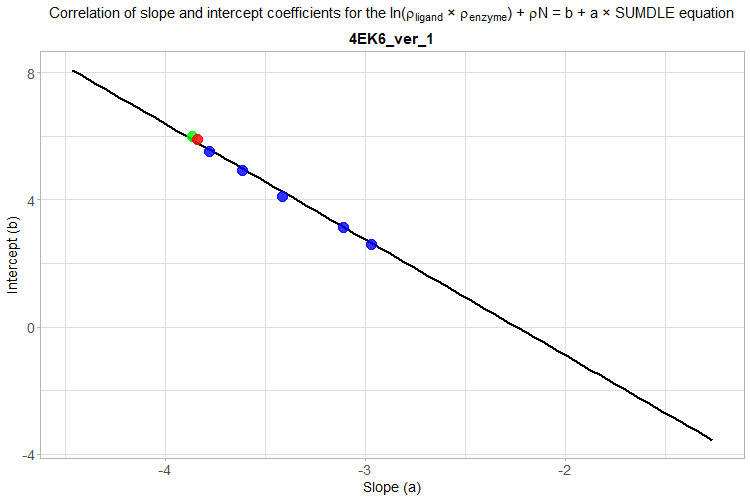

Supplement: Supplementary file 1 — ci0c01382_si_001.zip [file ci0c01382_si_001.zip › Supporting_Information/electron_density_data/CDK2/Coefficient data/4EK6_ver_1.png]

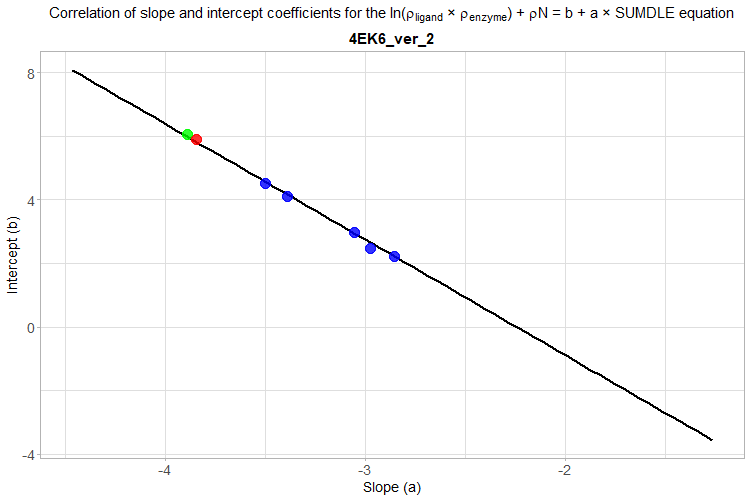

Supplement: Supplementary file 1 — ci0c01382_si_001.zip [file ci0c01382_si_001.zip › Supporting_Information/electron_density_data/CDK2/Coefficient data/4EK6_ver_2.png]

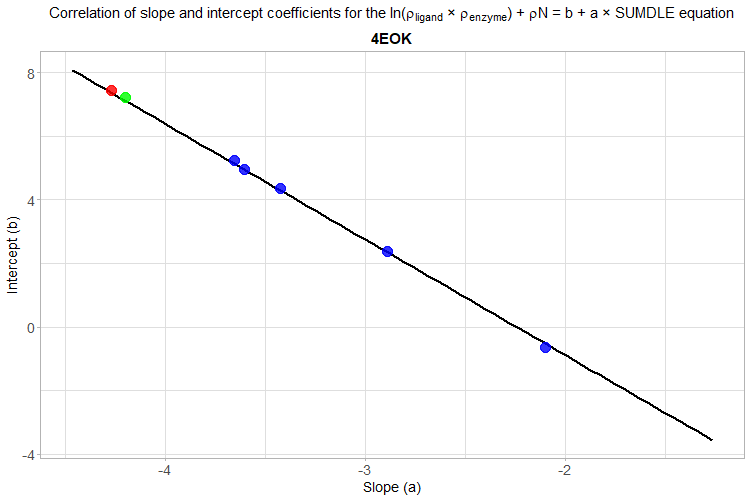

Supplement: Supplementary file 1 — ci0c01382_si_001.zip [file ci0c01382_si_001.zip › Supporting_Information/electron_density_data/CDK2/Coefficient data/4EOK.png]

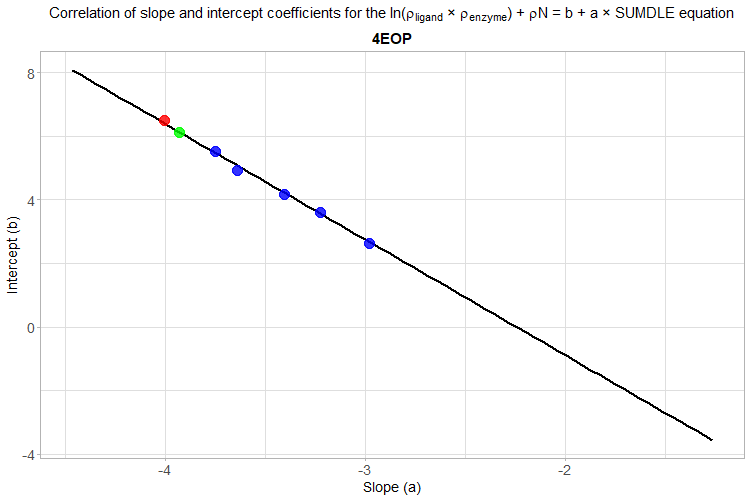

Supplement: Supplementary file 1 — ci0c01382_si_001.zip [file ci0c01382_si_001.zip › Supporting_Information/electron_density_data/CDK2/Coefficient data/4EOP.png]

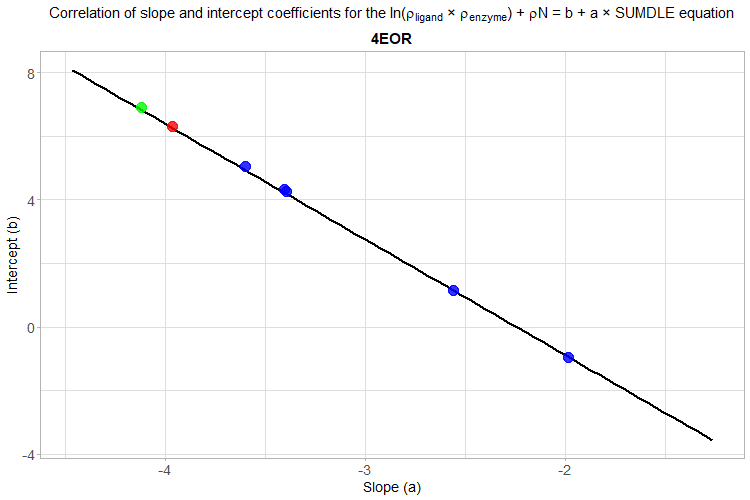

Supplement: Supplementary file 1 — ci0c01382_si_001.zip [file ci0c01382_si_001.zip › Supporting_Information/electron_density_data/CDK2/Coefficient data/4EOR.png]

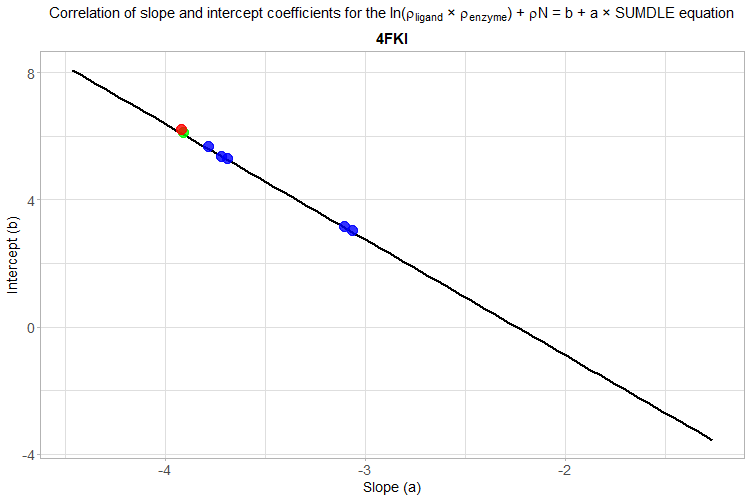

Supplement: Supplementary file 1 — ci0c01382_si_001.zip [file ci0c01382_si_001.zip › Supporting_Information/electron_density_data/CDK2/Coefficient data/4FKI.png]

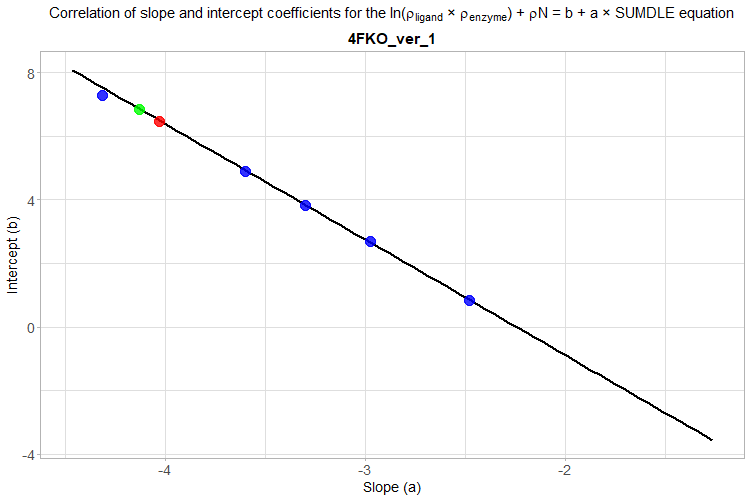

Supplement: Supplementary file 1 — ci0c01382_si_001.zip [file ci0c01382_si_001.zip › Supporting_Information/electron_density_data/CDK2/Coefficient data/4FKO_ver_1.png]

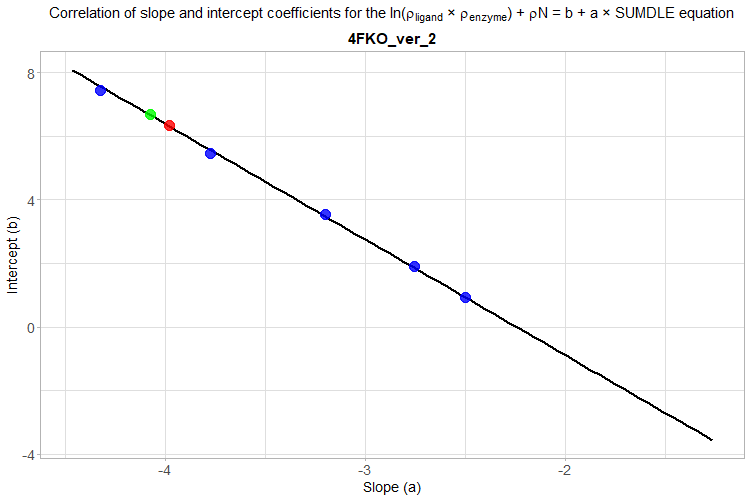

Supplement: Supplementary file 1 — ci0c01382_si_001.zip [file ci0c01382_si_001.zip › Supporting_Information/electron_density_data/CDK2/Coefficient data/4FKO_ver_2.png]

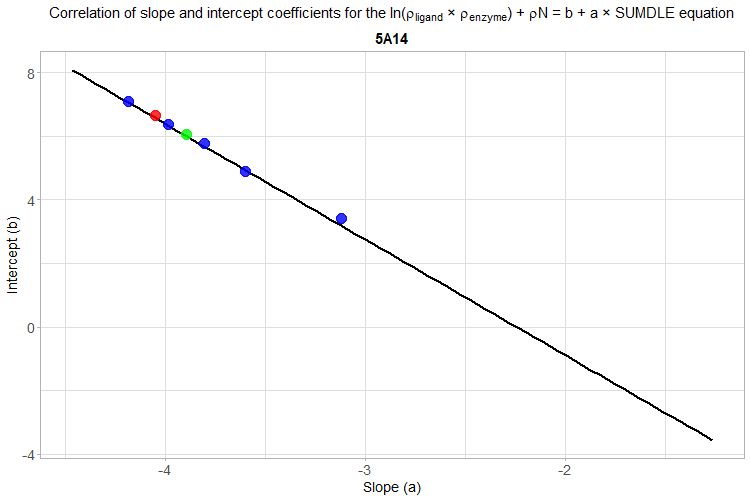

Supplement: Supplementary file 1 — ci0c01382_si_001.zip [file ci0c01382_si_001.zip › Supporting_Information/electron_density_data/CDK2/Coefficient data/5A14.png]

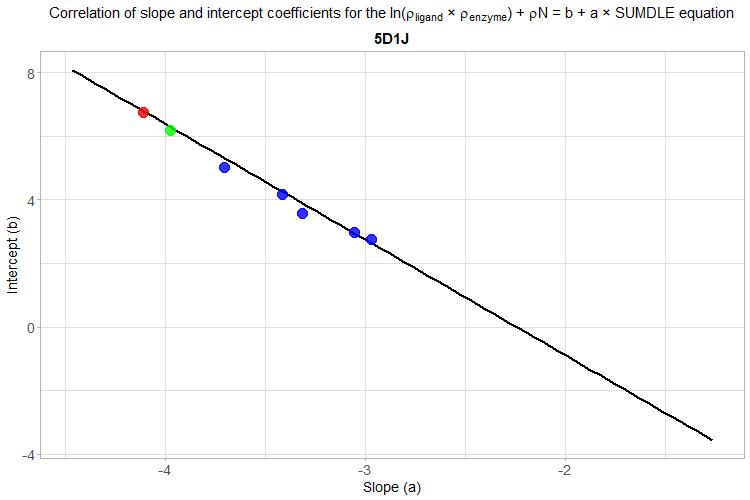

Supplement: Supplementary file 1 — ci0c01382_si_001.zip [file ci0c01382_si_001.zip › Supporting_Information/electron_density_data/CDK2/Coefficient data/5D1J.png]

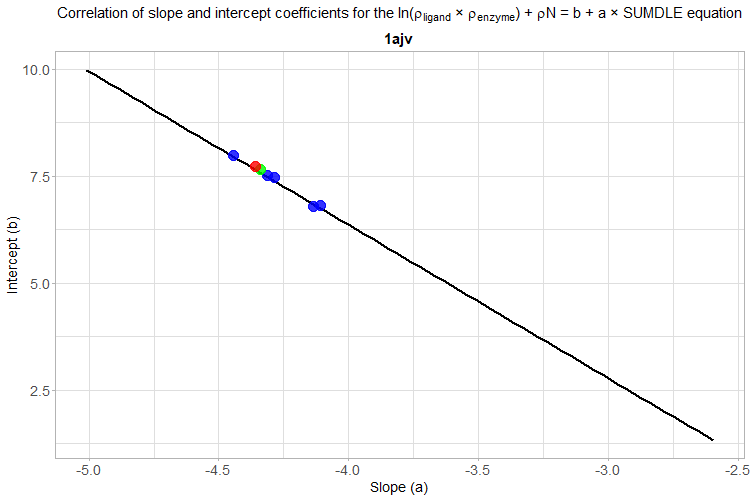

Supplement: Supplementary file 1 — ci0c01382_si_001.zip [file ci0c01382_si_001.zip › Supporting_Information/electron_density_data/HIV_1_protease/Coefficient data/1ajv.png]

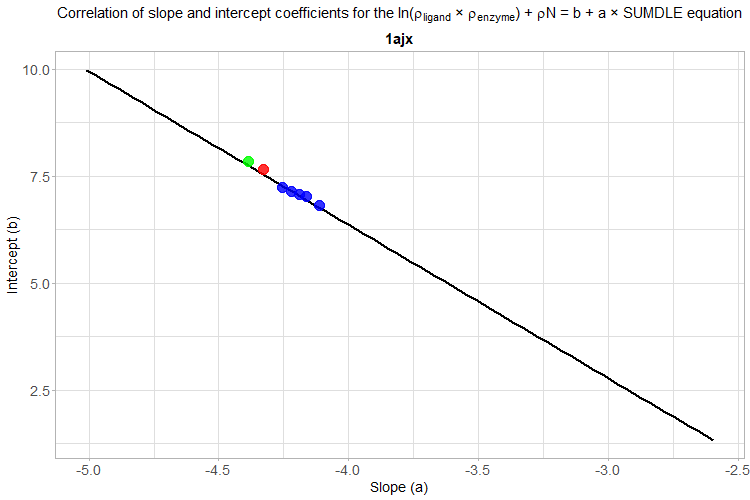

Supplement: Supplementary file 1 — ci0c01382_si_001.zip [file ci0c01382_si_001.zip › Supporting_Information/electron_density_data/HIV_1_protease/Coefficient data/1ajx.png]

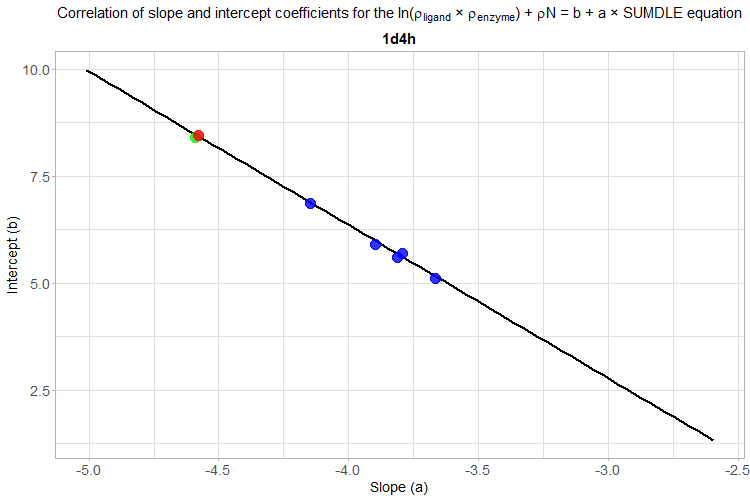

Supplement: Supplementary file 1 — ci0c01382_si_001.zip [file ci0c01382_si_001.zip › Supporting_Information/electron_density_data/HIV_1_protease/Coefficient data/1d4h.png]

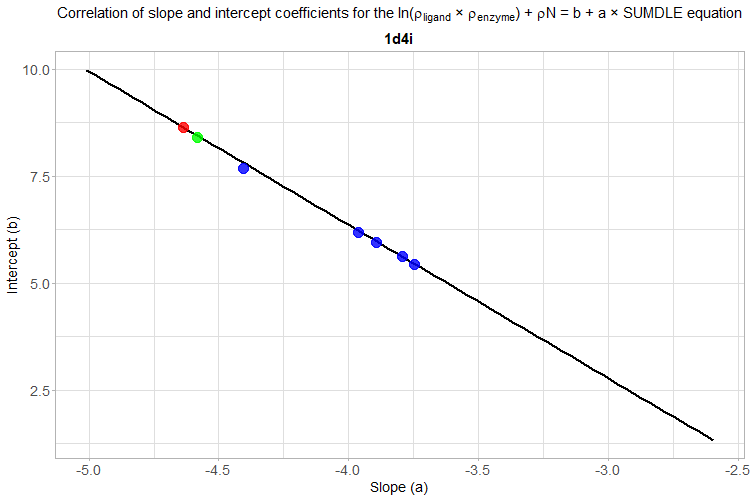

Supplement: Supplementary file 1 — ci0c01382_si_001.zip [file ci0c01382_si_001.zip › Supporting_Information/electron_density_data/HIV_1_protease/Coefficient data/1d4i.png]

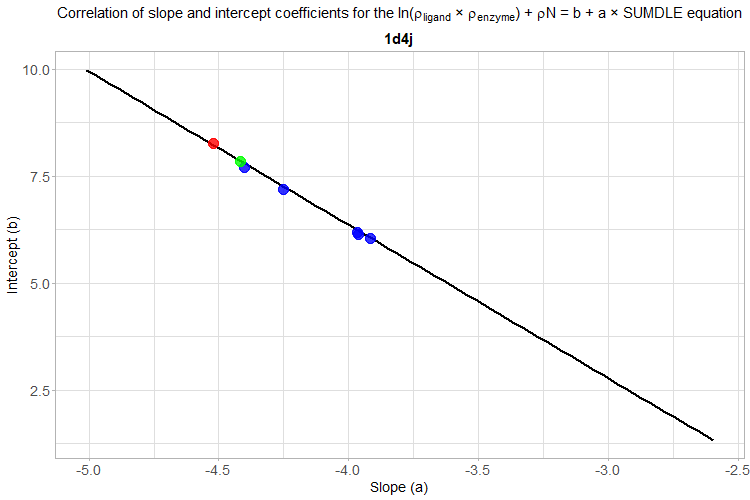

Supplement: Supplementary file 1 — ci0c01382_si_001.zip [file ci0c01382_si_001.zip › Supporting_Information/electron_density_data/HIV_1_protease/Coefficient data/1d4j.png]

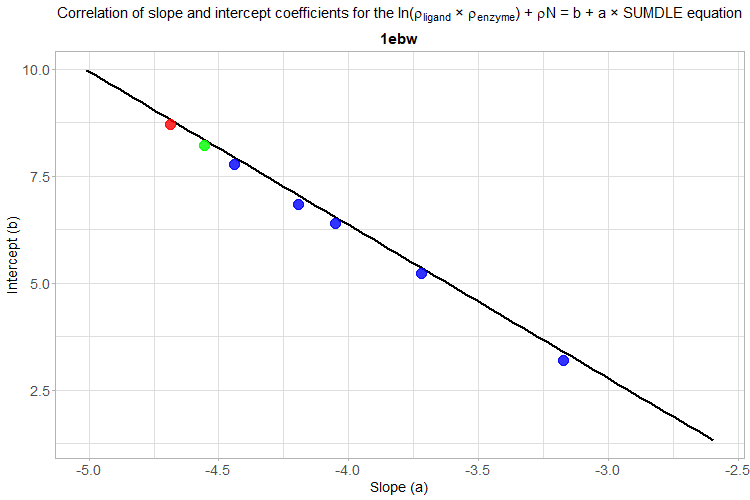

Supplement: Supplementary file 1 — ci0c01382_si_001.zip [file ci0c01382_si_001.zip › Supporting_Information/electron_density_data/HIV_1_protease/Coefficient data/1ebw.png]

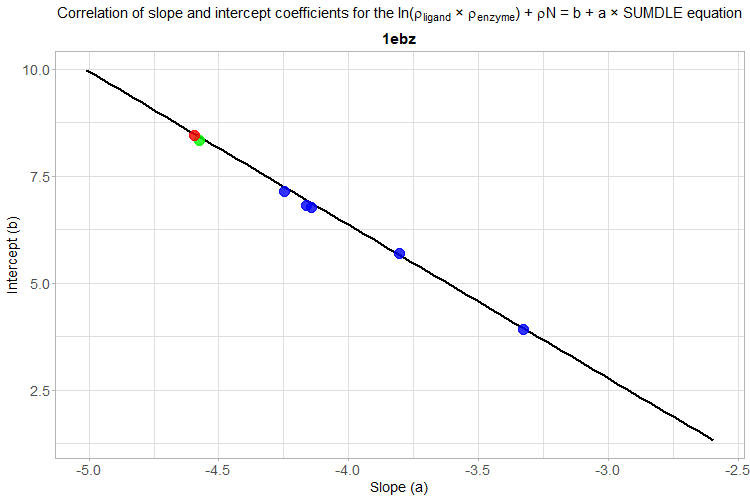

Supplement: Supplementary file 1 — ci0c01382_si_001.zip [file ci0c01382_si_001.zip › Supporting_Information/electron_density_data/HIV_1_protease/Coefficient data/1ebz.png]

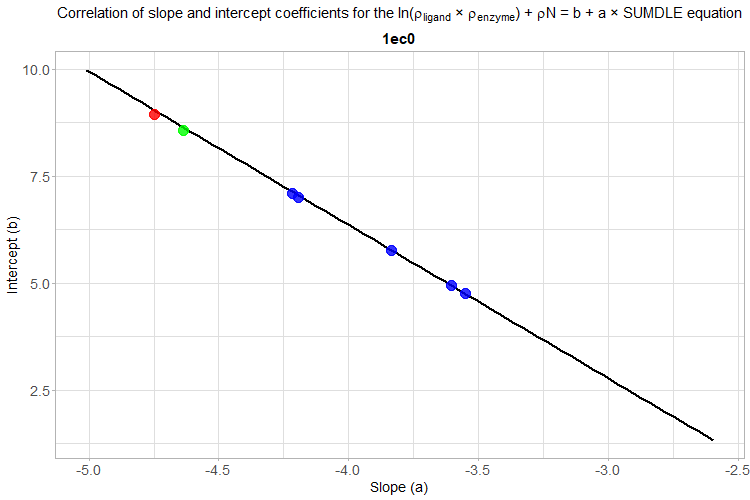

Supplement: Supplementary file 1 — ci0c01382_si_001.zip [file ci0c01382_si_001.zip › Supporting_Information/electron_density_data/HIV_1_protease/Coefficient data/1ec0.png]

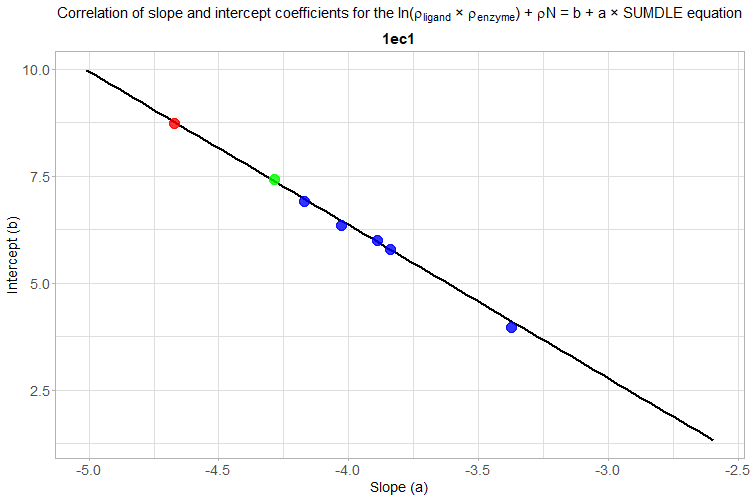

Supplement: Supplementary file 1 — ci0c01382_si_001.zip [file ci0c01382_si_001.zip › Supporting_Information/electron_density_data/HIV_1_protease/Coefficient data/1ec1.png]

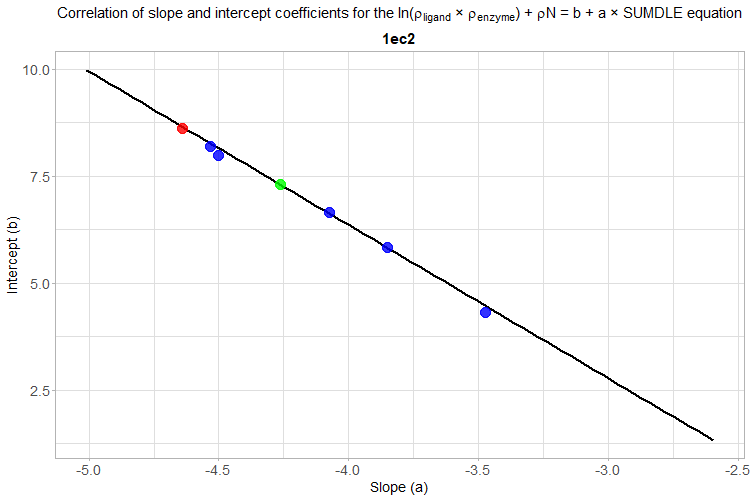

Supplement: Supplementary file 1 — ci0c01382_si_001.zip [file ci0c01382_si_001.zip › Supporting_Information/electron_density_data/HIV_1_protease/Coefficient data/1ec2.png]

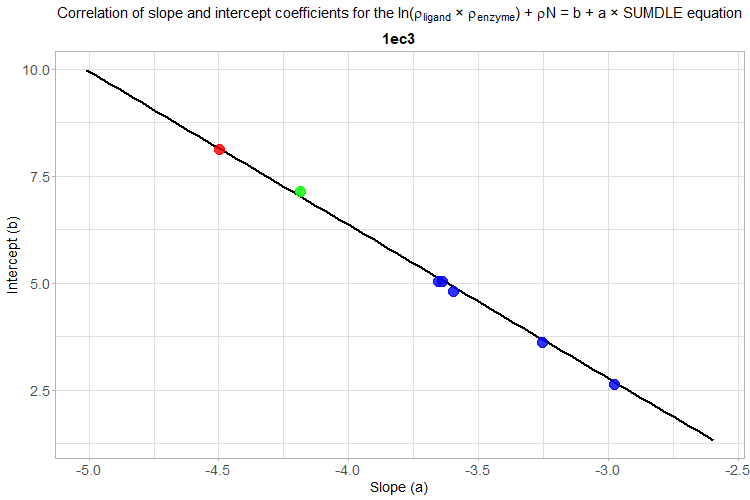

Supplement: Supplementary file 1 — ci0c01382_si_001.zip [file ci0c01382_si_001.zip › Supporting_Information/electron_density_data/HIV_1_protease/Coefficient data/1ec3.png]

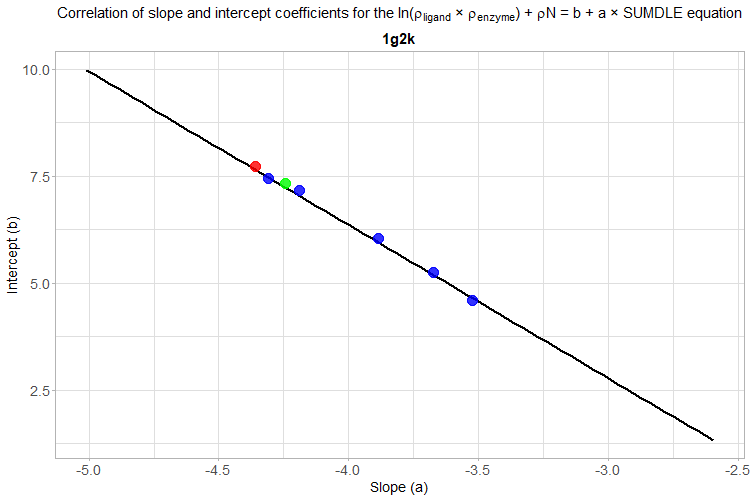

Supplement: Supplementary file 1 — ci0c01382_si_001.zip [file ci0c01382_si_001.zip › Supporting_Information/electron_density_data/HIV_1_protease/Coefficient data/1g2k.png]

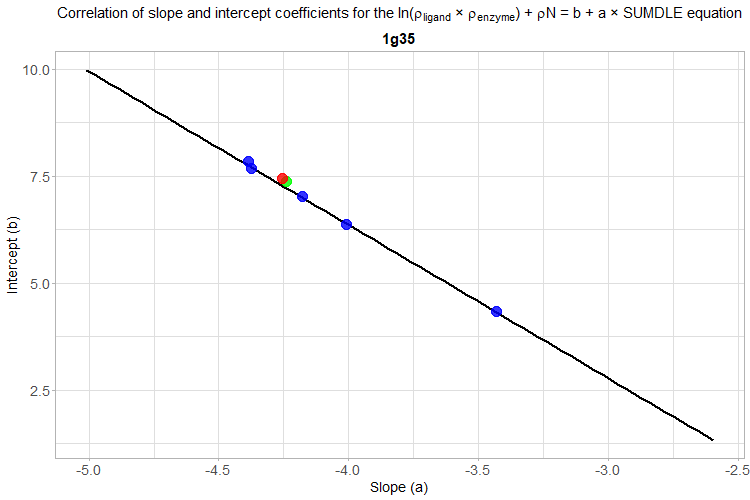

Supplement: Supplementary file 1 — ci0c01382_si_001.zip [file ci0c01382_si_001.zip › Supporting_Information/electron_density_data/HIV_1_protease/Coefficient data/1g35.png]

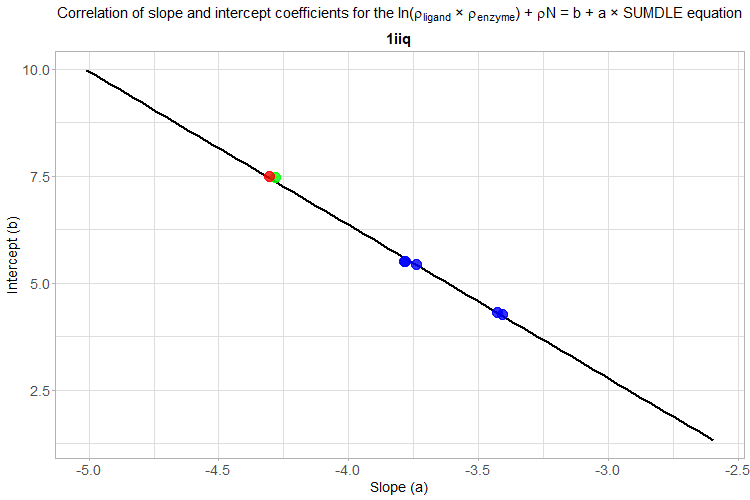

Supplement: Supplementary file 1 — ci0c01382_si_001.zip [file ci0c01382_si_001.zip › Supporting_Information/electron_density_data/HIV_1_protease/Coefficient data/1iiq.png]

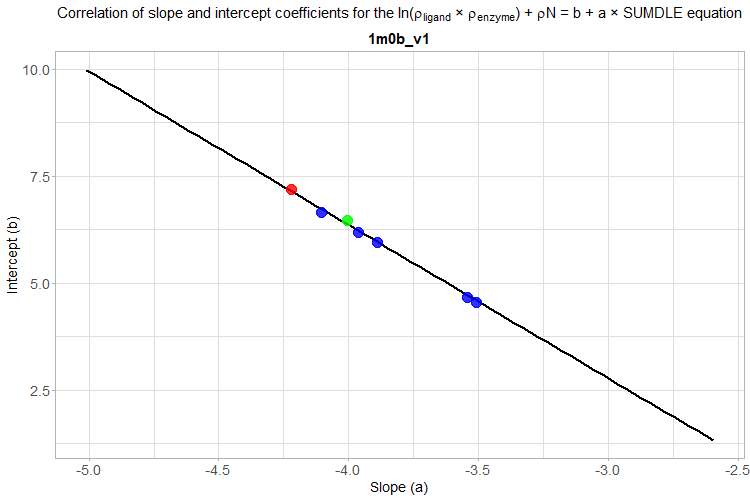

Supplement: Supplementary file 1 — ci0c01382_si_001.zip [file ci0c01382_si_001.zip › Supporting_Information/electron_density_data/HIV_1_protease/Coefficient data/1m0b_v1.png]

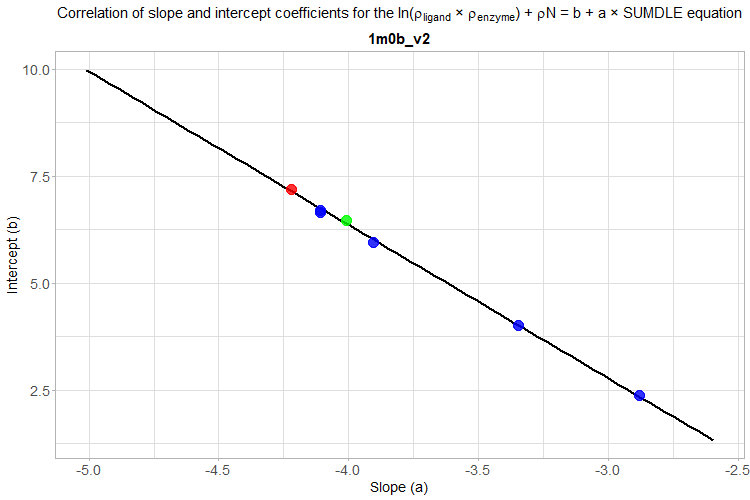

Supplement: Supplementary file 1 — ci0c01382_si_001.zip [file ci0c01382_si_001.zip › Supporting_Information/electron_density_data/HIV_1_protease/Coefficient data/1m0b_v2.png]
